# Supplementary material for: Discovery of fungal onoceroid triterpenoids through domainless enzyme-targeted global genome mining
Source: Nat Commun. 2024 May 21;15:4312. doi: 10.1038/s41467-024-48771-7 (PMC11109268; doi:10.1038/s41467-024-48771-7)
Supplement: Supplementary file 1 — Supplementary Information [file 41467_2024_48771_MOESM1_ESM.pdf]

Table of Contents

Supplementary Tables 1–7 **S2–S8**

Supplementary Fig. 1–79 **S9–S54**

Supplementary Table 1. Detection criteria of core proteins. If more than one protein domain is detected for a single region, only the protein domain with the highest bit score will be used for core enzyme identification.

Core protein		Detection criteria	
**Full name**	**Short name**	**Contains (required bit score)**	**Does not contain (required bit score)**
non-reducing polyketide synthase	NR-PKS	• PKS_KS (50)<br>• PKS_AT (50)<br>• fPKS_PT (30)	• fPKS_KR (30)<br>• AA-adenyl-dom (50)
partially reducing polyketide synthase	PR-PKS	• PKS_KS (50)<br>• PKS_AT (50)<br>• fPKS_TH (30)	• AA-adenyl-dom (50)
highly reducing polyketide synthase	HR-PKS	• PKS_KS (50)<br>• PKS_AT (50)<br>• fPKS_DH (30)	• AA-adenyl-dom (50)
type III polyketide synthase	T3PKS	• Chal_sti_synt_N or Chal_sti_synt_C	–
PKS-like enzyme	PKS-like	• PKS_KS (bit score > 50)<br>• PKS_AT (bit score > 50)	• fPKS_PT (30)<br>• fPKS_TH (30)<br>• fPKS_DH (30)
nonribosomal peptide synthetase	NRPS	• AA-adenyl-dom (50)<br>• NRPS_C (50) or NRPS_CT (50)	• PKS_KS (50)
NRPS-like enzyme	NRPS-like	• AA-adenyl-dom (50)<br>• fPKS_R or Thioesterase or Abhydrolase_3 or ACP_PCP	• NRPS_C (50)<br>• NRPS_CT (50)<br>• PKS_KS (50)
polyketide synthase-nonribosomal peptide synthetase	PKS-NRPS	• PKS_KS (50)<br>• AA-adenyl-dom (50)<br>*PKS_KS appears before AA-adenyl-dom	–
nonribosomal peptide synthetase-polyketide synthase	NRPS-PKS	• PKS_KS (50)<br>• AA-adenyl-dom (50)<br>*PKS_KS appears after AA-adenyl-dom	–
canonical class I terpene cyclase	TC (Class1)	• Terpene_syn_C_2 or Terpene_synth_C	• GGPS
Tri5-like terpene synthase	TC (Tri5)	• TRI5	–
squalene-hopene cyclase/oxidosqualene cyclase	TC (SHC/OSC)	• SQHop_cyclase_N or SQHop_cyclase_C	–
Pyr4-like terpene cyclase	TC (Pyr4)	• Pyr4	–
UbiA-like terpene synthase	TC (UbiA)	• UbiA_TC	–
PbcA-like terpene synthase	TC (PbcA)	• PbcA	• GGPS
AstC-like terpene synthase	TC (AstC)	• AstC (250)	–
ABA3-like terpene synthase	TC (ABA3)	• ABA3	–
AsR6-like terpene synthase	TC (AsR6)	• AsR6	–
bifunctional prenyltransferase/terpene cyclase	chimeric TS	• Terpene_syn_C_2 or Terpene_synth_C or PbcA<br>• GGPS	–
UbiA-like prenyltransferase	PT (UbiA)	• UbiA_PT	–
IPPS-type prenyltransferase	PT (IPPS)	• PaxC	–
DMATS-type prenyltransferase	PT (DMATS)	• Trp_DMAT	–
polyprenyl pyrophosphate synthase	PPPS	• GGPS	• Terpene_syn_C_2 or Terpene_synth_C or PbcA
arginine-containing cyclodipeptide synthase	RCDPS	• AnkA	–
ε-poly-L-lysine synthetase	ePLS	• AA-adenyl-dom (50)<br>• NRPS_term_dom	• PKS_KS (50)
ribosomally synthesized and posttranslationally modified peptide precursor peptide	RiPP PP	See the Methods section for the detection criteria.	

Supplementary Table 2. Annotation of each gene in the *homo*, *fumi*, and *alli* clusters.

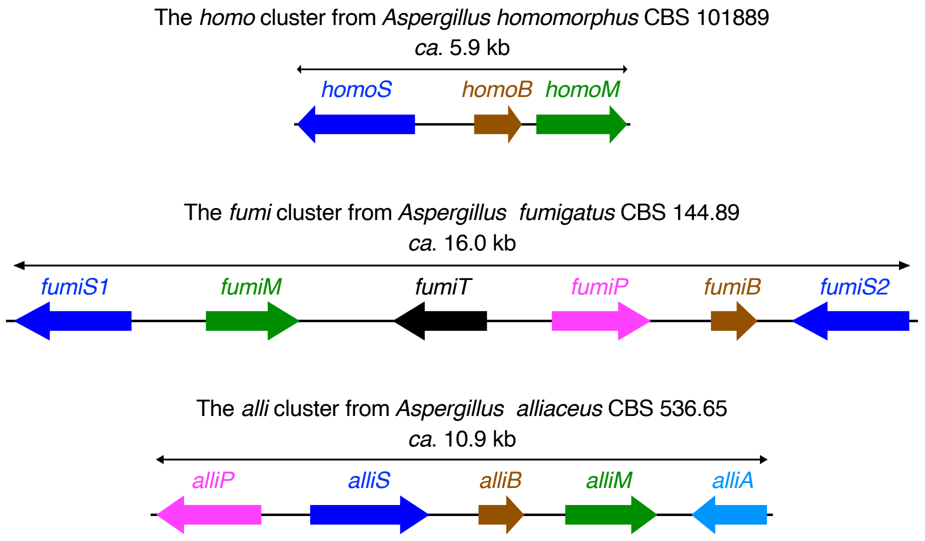

Gene	Sequence ID[a]	Amino acids (base pairs)	Protein homologue (origin)	Similarity/Identity (%)	Proposed function
homoS	XP_025551549.1	704 (2115)	AfumA (*Aspergillus fumigatus*)	61/47	(oxido)squalene cyclase
homoM[b]	XP_025551547.1	469 (1626)	OlcE (*Penicillium canescens*)	65/44	FAD-dependent monooxygenase
homoB	XP_025551548.1	257 (852)	DpmpB (*Macrophomina phaseolina*)	67/50	Pyr4-family terpene cyclase
fumiS1	EDP47976.1	689 (2070)	AfumA (*Aspergillus fumigatus*)	61/46	(oxido)squalene cyclase
fumiS2[c]	–	689 (2070)	AfumA (*Aspergillus fumigatus*)	47/32	(oxido)squalene cyclase
fumiM	EDP47977.1	482 (1643)	OlcE (*Penicillium canescens*)	65/48	FAD-dependent monooxygenase
fumiB	EDP47980.1	245 (806)	PenB (*Penicillium crustosum*)	62/42	Pyr4-family terpene cyclase
fumiP[b]	EDP47979.1	500 (1740)	PrhB (*Penicillium brasilianum*)	69/53	cytochrome P450 monooxygenase
fumiT[b]	EDP47978.1	435 (1653)	–	–	major facilitator superfamily transporter
alliS	XP_031902717.1	700 (2103)	AfumA (*Aspergillus fumigatus*)	61/46	(oxido)squalene cyclase
alliM[b]	XP_031902719.1	485 (1624)	OlcE (*Penicillium canescens*)	68/49	FAD-dependent monooxygenase
alliB[b]	XP_031902718.1	245 (796)	DpmpB (*Macrophomina phaseolina*)	66/53	Pyr4-family terpene cyclase
alliP[b]	XP_031902716.1	535 (1852)	InaC (*Aspergillus flavus*)	54/33	cytochrome P450 monooxygenase
alliA[b]	XP_031902720.1	441 (1326)	AtnC (*Arthrinium* sp.)	54/35	acetyltransferase

[a]Sequence IDs are as designated in the NCBI database. [b]The sequences of these genes were manually revised (see Supplementary Data 8. [c]The gene was manually added (see Supplementary Data 8).

Supplementary Table 3. Annotation of each gene in the *mos* cluster.

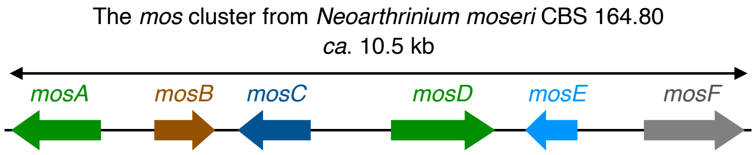

Gene	Sequence ID[a]	Amino acids (base pairs)	Protein homologue (origin)	Similarity/Identity (%)	Proposed function
mosA	XP_049167925.1	420 (1263)	PhnG (*Penicillium herquei*)	48/28	FAD-dependent oxidoreductase
mosB	XP_049167926.1	247 (851)	MacJ (*Penicillium terrestre*)	51/32	Pyr4-family terpene cyclase
mosC	XP_049167927.1	341 (1026)	AscA (*Acremonium egyptiacum*)	51/32	UbiA-like prenyltransferase
mosD	XP_049167928.1	440 (1470)	BisD (*Biscogniauxia* sp. 71-10-1-1)	75/60	FAD-dependent monooxygenase
mosE[b]	XP_049167929.1	217 (729)	-	-	*N*-acetyltransferase
mosF[b]	XP_049167930.1	352 (1422)	Ba17b (*Metarhizium anisopliae*)	43/27	hypothetical protein

[a]Sequence IDs are as designated in the NCBI database. [b]The sequences of these genes were manually revised (see Supplementary Data 8).

Supplementary Table 4. Plasmids constructed in this study and PCR conditions for the amplification of the inserts for the plasmid constructions.

Plasmid	Inserts	Primer 1	Primer 2	PCR Template	Vector
pTAex3-HR-homoS	*homoS*	homoS-F	homoS-R	gDNA	pTAex3-HR digested with *Sma*I
pTAex3-HR-homoM	*homoM*	homoM-F	homoM-R	gDNA	pTAex3-HR digested with *Sma*I
pTAex3-HR-homoB	*homoB*	homoB-F	homoB-R	gDNA	pTAex3-HR digested with *Sma*I
pTAex3-HR-fumiS1	*fumiS1*	fumiS1-F	fumiS1-R	gDNA	pTAex3-HR digested with *Sma*I
pTAex3-HR-fumiS2	*fumiS2*	fumiS2-F	fumiS2-R	gDNA	pTAex3-HR digested with *Sma*I
pTAex3-HR-fumiM	*fumiM*	fumiM-F	fumiM-R	gDNA	pTAex3-HR digested with *Sma*I
pTAex3-HR-fumiB	*fumiB*	fumiB-F	fumiB-R	gDNA	pTAex3-HR digested with *Sma*I
pTAex3-HR-fumiP	*fumiP*	fumiP-F	fumiP-R	gDNA	pTAex3-HR digested with *Sma*I
pTAex3-HR-alliS	*alliS*	alliS-F	alliS-R	gDNA	pTAex3-HR digested with *Sma*I
pTAex3-HR-alliM	*alliM*	alliM-F	alliM-R	gDNA	pTAex3-HR digested with *Sma*I
pTAex3-HR-alliB	*alliB*	alliB-F	alliB-R	gDNA	pTAex3-HR digested with *Sma*I
pTAex3-HR-alliP	*alliP*	alliP-F	alliP-R	gDNA	pTAex3-HR digested with *Sma*I
pTAex3-HR-alliA	*alliA*	alliA-F	alliA-R	gDNA	pTAex3-HR digested with *Sma*I
pTAex3-HR-homoS+homoB+homoM	*PamyB-homoB-TamyB*<br>*PamyB-homoM-TamyB*	InF-pTAex3_SdaI-F<br>InF-Linker-F1	InF-Linker-R1<br>InF-pTAex3_SdaI-R	pTAex3-HR-homoB<br>pTAex3-HR-homoM	pTAex3-HR-homoS digested with *Sda*I
pAdeA-HR-fumiS1+fumiB	*PamyB-fumiS1-TamyB*<br>*PamyB-fumiB-TamyB*	InF-pAdeA_XbaI-F<br>InF-Linker-F1	InF-Linker-R1<br>nF-pAdeA_XbaI-R	pTAex3-HR-fumiS1<br>pTAex3-HR-fumiB	pAdeA-HR digested with *Xba*I
pTAex3-HR-fumiP+fumiM	*PamyB-fumiM-TamyB*	InF-pTAex3_SdaI-F	InF-pTAex3_SdaI-R	pTAex3-HR-fumiM	pTAex3-HR-fumiP digested with *Sda*I
pAdeA-HR-alliS+alliB	*PamyB-alliS-TamyB*<br>*PamyB-alliB-TamyB*	InF-pAdeA_XbaI-F<br>InF-Linker-F1	InF-Linker-R1<br>nF-pAdeA_XbaI-R	pTAex3-HR-alliS<br>pTAex3-HR-alliB	pAdeA-HR digested with *Xba*I
pTAex3-HR-alliM+alliP	*PamyB-alliP-TamyB*	InF-pTAex3_SdaI-F	InF-pTAex3_SdaI-R	pTAex3-HR-alliP	pTAex3-HR-alliM digested with *Sda*I
pTAex3-HR-alliM+alliA	*PamyB-alliA-TamyB*	InF-pTAex3_SdaI-F	InF-pTAex3_SdaI-R	pTAex3-HR-alliA	pTAex3-HR-alliM digested with *Sda*I
pTAex3-HR-alliM+alliA+alliP	*PamyB-alliA-TamyB*<br>*PamyB-alliP-TamyB*	InF-pTAex3_SdaI-F<br>InF-Linker-F1	InF-Linker-R1<br>InF-pTAex3_SdaI-R	pTAex3-HR-alliA<br>pTAex3-HR-alliP	pTAex3-HR-alliM digested with *Sda*I
pTAex3-HR-mosA	*mosA*	mosA-F	mosA-R	gDNA	pTAex3-HR digested with *Sma*I
pPyrG-HR-mosB	*mosB*	mosB-F	mosB-R	gDNA	pPyrG-HR digested with *Sma*I
pTAex3-HR-mosC	*mosC*	mosC-F	mosC-R	gDNA	pTAex3-HR digested with *Sma*I
pTAex3-HR-mosD	*mosD*	mosD-F	mosD-R	gDNA	pTAex3-HR digested with *Sma*I
pTAex3-HR-mosE	*mosE*	mosE-F	mosE-R	gDNA	pTAex3-HR digested with *Sma*I
pTAex3-HR-mosF	*mosF*	mosF-F	mosF-R	gDNA	pTAex3-HR digested with *Sma*I
pPyrG-HR-mosB+mosC+mosD	*PamyB-mosC-TamyB*<br>*PamyB-mosD-TamyB*	InF-pUSA_BamHI-F<br>InF-Linker-F1	InF-Linker-R1<br>InF- pUSA_BamHI-R	pTAex3-HR-mosC<br>pTAex3-HR-mosD	pPyrG-HR-mosB digested with *Bam*HI
pAdeA-HR-mosE+mosF	*PamyB-mosE-TamyB*<br>*PamyB-mosF-TamyB*	InF-pAdeA_XbaI-F<br>InF-Linker-F1	InF-Linker-R1<br>nF-pAdeA_XbaI-R	pTAex3-HR-mosE<br>pTAex3-HR-mosF	pAdeA-HR digested with *Xba*I
pAdeA-HR-mosE+mosF+mosA	*PamyB-mosA-TamyB*	InF-pAdeA_SpeI-F	InF-pAdeA_SpeI-R	pTAex3-HR-mosA	pAdeA-HR-mosE+mosF digested with *Spe*I

Supplementary Table 5. *Aspergillus oryzae* transformants constructed in this study.

Strain	Host strain	Plasmids used for transformation
A. oryzae/*homoS*	*A. oryzae* NSAR1	pTAex3-HR-homoS
A. oryzae/*homoS+M+B*	*A. oryzae* NSAR1	pTAex3-HR-homoS+homoB+homoM
A. oryzae/*fumiS1*	*A. oryzae* NSAR1	pTAex3-HR-fumiS1
A. oryzae/*fumiS2*	*A. oryzae* NSAR1	pTAex3-HR-fumiS2
A. oryzae/*fumiS1+M+B*	*A. oryzae* NSAR1	pTAex3-HR-fumiM, pAdeA-HR-fumiS1+fumiB
A. oryzae/*fumiS1+M+B+P*	*A. oryzae* NSAR1	pTAex3-HR-fumiP+fumiM, pAdeA-HR-fumiS1+fumiB
A. oryzae/*alliS*	*A. oryzae* NSAR1	pTAex3-HR-alliS
A. oryzae/*alliS+M+B*	*A. oryzae* NSAR1	pTAex3-HR-alliB, pAdeA-HR-alliS+alliM
A. oryzae/*alliS+M+B+P*	*A. oryzae* NSAR1	pTAex3-HR-alliM+alliP, pAdeA-HR-alliS+alliB
A. oryzae/*alliS+M+B+A*	*A. oryzae* NSAR1	pTAex3-HR-alliM+alliA, pAdeA-HR-alliS+alliB
A. oryzae/*alliS+M+B+P+A*	*A. oryzae* NSAR1	pTAex3-HR-alliM+alliA+alliP, pAdeA-HR-alliS+alliB
A. oryzae/*mosA+B+C+D+E+F*	*A. oryzae* NSARU1	pPyrG-HR-mosB+mosC+mosD, pAdeA-HR-mosE+mosF+mosA
A. oryzae/*mosB+C+D*	*A. oryzae* NSARU1	pPyrG-HR-mosB+mosC+mosD

Supplementary Table 6. ^{1}H NMR data of the triterpenoids obtained in this study.

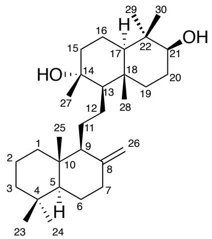
4
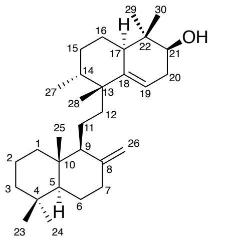
5
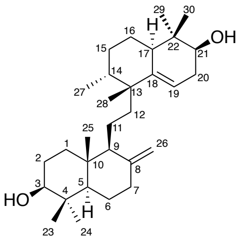
6
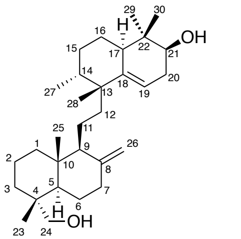
7
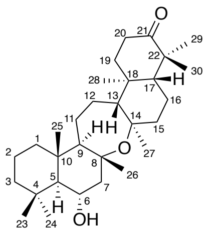
9
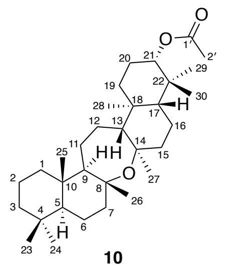
10
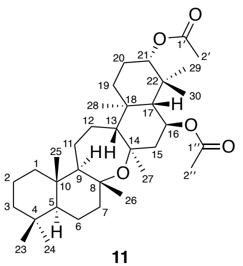
11

	δH, mult. (*J* in Hz)						
position	**4**[a]	**5**[b]	**6**[b]	**7**[b]	**9**[b]	**10**[b]	**11**[a]
1	1.01 (α), m	1.00 (α), td (13.2, 3.9)	1.16 (α), m	1.02 (α), td (13.0, 4.4)	0.93 (α), td (12.5, 3.7)	0.87 (α), m	0.78 (α), m
	1.85 (β), dt (13.0, 4.0)	1.73 (β), m	1.77 (β), m	1.74 (β), m	1.74 (β), m	1.77 (β), m	1.72 (β), m
2	1.48 (α), ddd (14.8, 6.7, 3.4)	1.47 (α), m	1.70 (α), m	1.60 (α), m	1.43 (α), m	1.42 (α), dq (14.2, 3.5)	1.41 (α), m
	1.61 (β), m	1.56 (β), qt (13.7, 3.3)	1.58 (β), m	1.57 (β), m	1.57 (β), m	1.59 (β), m	1.59 (β), qt (13.7, 3.3)
3	1.19 (α), td (13.0, 3.9)	1.16 (α), td (13.5, 4.0)	3.24, dd (11.8, 3.8)	1.42 (α), m	1.19 (α), m	1.11 (α), td (13.4, 4.0)	1.13 (α), m
	1.40 (β), dt (12.8, 3.4)	1.38 (β), m		1.28 (β), m	1.34 (β), m	1.34 (β), dt (13.7, 3.5)	1.36 (β), m
5	1.06, dd (12.8, 2.7)	1.07, dd (12.7, 2.7)	1.06, brd (12.5)	1.41, m	0.97, brd (10.2)	0.87, m	0.81, m
6	1.68 (α), m	1.71 (α), m	1.73 (α), m	1.61 (α), m	3.77, brt (10.6)	1.58 (α), m	1.54 (α), m
	1.35 (β), qd (13.0, 4.4)	1.29 (β), qd (13.0, 4.3)	1.36 (β), qd (12.7, 3.6)	1.31 (β), qd (12.9, 4.3)		1.22 (β), m	1.14 (β), m
7	2.06 (α), td (12.9, 4.9)	1.97 (α), td (12.8, 5.0)	1.97 (α), td (12.8, 4.5)	2.00 (α), td (12.8, 5.1)	1.66 (α), t (11.7)	1.56 (α), m	1.71 (α), m
	2.46 (β), ddd (12.7, 4.0, 2.4)	2.36 (β), ddd (12.8, 4.0, 2.4)	2.38 (β), brd (13.0)	2.35 (β), ddd (12.5, 4.0, 2.4)	2.09 (β), dd (11.8, 3.0)	1.75 (β), m	1.90 (β), m
9	1.60, m	1.49, brd (10.4)	1.46, brd (10.2)	1.56, m	1.43, m	1.38, dd (8.6, 3.5)	1.41, m
11	1.69, m	1.20, m	1.17, m	1.20, m	1.82 (α), m	1.75 (α), m	1.68 (α), m
	1.55, td (12.8, 4.8)	0.95, m	0.97, m	0.96, m	1.22 (β), m	1.22 (β), m	1.18 (β), m
12	1.83, m	1.70, m	1.71, m	1.70, td (13.0, 5.0)	1.29 (α), m	1.22 (α), m	1.07 (α), m
	0.94, m	0.74, td (12.7, 4.0)	0.73, td (11.8, 3.0)	0.74, m	1.75 (β), m	1.75 (β), m	1.54 (β), m
13	0.85, m				1.48, brd (11.0)	1.38, dd (8.6, 3.5)	1.27, brd (10.6)
14		1.27, m	1.27, m	1.27, m			
15	1.21 (α), ddd (14.3, 10.6, 3.7)	1.38 (α), m	1.38 (α), m	1.38 (α), qd (13.0, 3.2)	1.80 (α), m	1.75 (α), m	2.32 (α), dd (11.9, 3.6)
	1.67 (β), m	1.44 (β), m	1.46 (β), m	1.46 (β), m	1.60 (β), m	1.56 (β), m	1.90 (β), m
16	1.45 (α), m	1.80 (α), m	1.79 (α), m	1.80 (α), m	1.37 (α), m	1.29 (α), m	5.32, td (11.4, 3.4)
	1.09 (β), qd (13.4, 3.0)	1.26 (β), m	1.26 (β), m	1.25 (β), m	1.53 (β), m	1.58 (β), m	
17	0.70, dd (12.2, 2.1)	1.78, m	1.77, m	1.78, m	1.51, dd (12.6, 1.9)	0.95, dd (12.6, 1.9)	1.25, d (11.4)
19	0.97 (α), m	5.33, t (3.6)	5.32, brs	5.33, t (3.7)	1.99 (α), dt (12.8, 6.0)	1.80 (α), dt (13.2, 3.5)	1.46 (α), m
	1.61 (β), m				1.53 (β), m	1.11 (β), td (13.4, 4.0)	0.76 (β), m
20	1.45, m	2.33 (α), m	2.33 (α), m	2.33 (α), m	2.47, m	1.62 (α), td (13.1, 3.9)	1.53 (α), m
		2.09 (β), m	2.09 (β), m	2.09 (β), m		1.67 (β), m	1.73 (β), m
21	3.04, dd (10.3, 5.9)	3.44, dd (7.2, 5.1)	3.44, brt (5.5)	3.44, dd (7.2, 5.0)		4.46, dd (11.8, 4.8)	4.65, dd (11.9, 4.5)
23	0.84, s	0.78, s	0.76, s	0.74, s	0.98, s	0.77, s	0.80, s
24	0.90, s	0.86, s	0.98, s	3.40, d (10.9)	1.15, s	0.84, s	0.86, s
				3.10, d (10.9)			
25	0.84, s	0.61, s	0.62, s	0.66, s	0.78, s	0.73, s	0.75, s
26	5.16, brs	4.76, brs	4.78, brs	4.77, brs	1.32, s	1.25, s	1.33, s
	5.09, brd (0.74)	4.44, brs	4.46, brs	4.45, brs			
27	1.02, s	0.83, d (6.7)	0.83, d (7.3)	0.83, d (7.0)	1.29, s	1.26, s	1.46, s
28	0.65, s	1.01, s	1.00, s	1.01, s	0.83, s	0.77, s	0.69, s
29	0.99, s	0.95, s	0.95, s	0.95, s	1.00, s	0.81, s	1.03, s
30	0.73, s	0.84, s	0.84, s	0.84, s	1.08, s	0.84, s	1.19, s
2′						2.04, s	1.77, s
2′′							1.70, s

[a]600 MHz in C_6D_6, [b]600 MHz in $CDCl_3$.

Supplementary Table 7. ^{13}C NMR data of the triterpenoids obtained in this study.

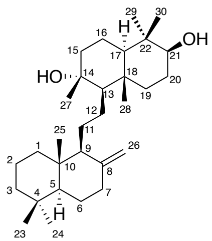
4

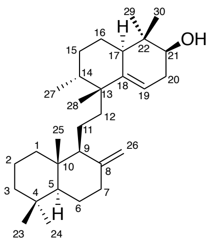
5

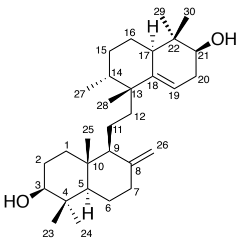
6

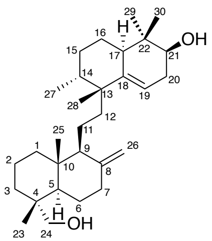
7

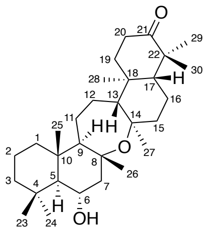
9

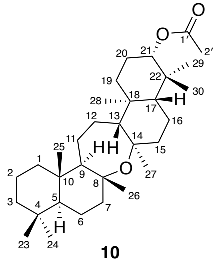
10

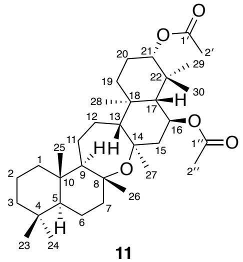
11

	δ_C, type						
position	**4**[a]	**5**[b]	**6**[b]	**7**[b]	**9**[b]	**10**[b]	**11**[a]
1	39.4, CH_2	39.0, CH_2	37.0, CH_2	38.5, CH_2	40.4, CH_2	40.3, CH_2	40.5, CH_2
2	19.9, CH_2	19.4, CH_2	28.0, CH_2	18.7, CH_2	18.6, CH_2	18.9, CH_2	19.3, CH_2
3	42.6, CH_2	42.2, CH_2	78.9, CH	35.5, CH_2	43.3, CH_2	42.0, CH_2	42.3, CH_2
4	33.8, C	33.6, C	39.1, C	38.0, C	33.8, C	33.4, C	33.6, C
5	55.8, CH	55.6, CH	54.7, CH	48.6, CH	61.0, CH	56.2, CH	56.5, CH
6	24.8, CH_2	24.5, CH_2	24.0, CH_2	24.3, CH_2	69.4, CH	20.7, CH_2	21.2, CH_2
7	38.9, CH_2	38.5, CH_2	38.3, CH_2	38.2, CH_2	56.2, CH_2	45.2, CH_2	45.3, CH_2
8	148.6, C	149.6, C	148.9, C	149.3, C	79.4, C	80.1, C	80.6, C
9	58.6, CH	58.1, CH	57.7, CH	58.0, CH	60.0, CH	60.7, CH	60.3, CH
10	39.9, C	40.0, C	39.7, C	39.9, C	39.4, C	38.8, C	39.4, C
11	28.2, CH_2	17.4, CH_2	17.6, CH_2	17.5, CH_2	24.8, CH_2	24.8[c], CH_2	24.9, CH_2
12	25.7, CH_2	30.2, CH_2	30.1, CH_2	30.2, CH_2	25.5, CH_2	25.0[c], CH_2	25.1, CH_2
13	62.4, CH	42.7, C	42.6, C	42.7, C	59.6, CH	60.4, CH	60.5, CH
14	73.7, C	43.1, CH	43.1, CH	43.1, CH	79.7, C	79.6, C	79.2, C
15	45.0, CH_2	30.9, CH_2	30.8, CH_2	30.8, CH_2	44.4, CH_2	45.1, CH_2	52.7, CH_2
16	20.6, CH_2	28.7, CH_2	28.7, CH_2	28.7, CH_2	21.7, CH_2	20.3, CH_2	70.2, CH
17	55.2, CH	44.3, CH	44.3, CH	44.3, CH	54.4, CH	55.1, CH	58.2, CH
18	38.9, C	144.8, C	144.7, C	144.8, C	38.4, C	38.4, C	39.2, C
19	38.0, CH_2	114.5, CH	114.6, CH	114.5, CH	39.2, CH_2	38.2, CH_2	38.1, CH_2
20	27.7, CH_2	31.4, CH_2	31.4, CH_2	31.4, CH_2	33.9, CH_2	23.8, CH_2	23.7, CH_2
21	78.5, CH	75.0, CH	75.0, CH	75.0, CH	217.8, C	80.8, CH	80.0, CH
22	39.0, C	36.8, C	36.8, C	36.8, C	47.2, C	37.8, C	38.1, C
23	22.0, CH_3	21.7, CH_3	15.3, CH_3	17.6, CH_3	22.2, CH_3	21.5, CH_3	21.7, CH_3
24	33.9, CH_3	33.6, CH_3	28.3, CH_3	72.1, CH_2	36.4, CH_3	33.4, CH_3	33.6, CH_3
25	15.0, CH_3	14.5, CH_3	14.5, CH_3	15.0, CH_3	17.3, CH_3	15.8, CH_3	16.1, CH_3
26	107.8, CH_2	105.8, CH_2	106.2, CH_2	106.0, CH_2	26.7, CH_3	25.2, CH_3	25.8, CH_3
27	24.2, CH_3	16.5, CH_3	16.5, CH_3	16.5, CH_3	24.7, CH_3	25.2, CH_3	26.4, CH_3
28	15.7, CH_3	22.7, CH_3	22.7, CH_3	22.7, CH_3	15.7, CH_3	15.9, CH_3	16.9, CH_3
29	28.4, CH_3	26.3, CH_3	26.3, CH_3	26.3, CH_3	20.9, CH_3	16.4, CH_3	17.2, CH_3
30	15.6, CH_3	18.2, CH_3	18.2, CH_3	18.2, CH_3	27.1, CH_3	28.1, CH_3	30.5, CH_3
1′						171.0, C	170.1, C
2′						21.3, CH_3	20.9, CH_3
1′′							169.3, C
2′′							21.5, CH_3

[a]150 MHz in C_6D_6, [b]150 MHz in $CDCl_3$, [c]These signals are interchangeable.

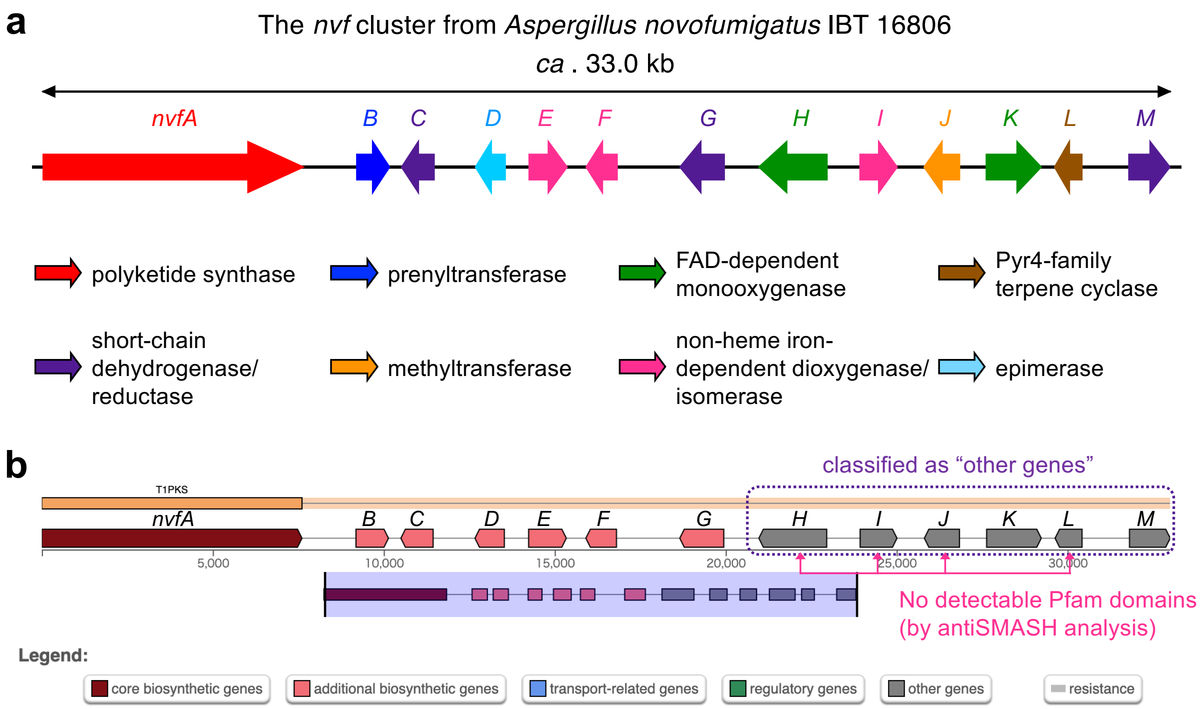

Supplementary Fig. 1. Biosynthetic gene cluster of novofumigatonin (the *nvf* cluster). **a** Schematic representation of the *nvf* cluster. **b** Analysis result of the *nvf* cluster by antiSMASH (version 7.0.0). Six out of 13 genes were classified as other genes (*nvfH–M*), four of which (*nvfH*, *-I*, *-J*, and *-L*) lack a detectable Pfam domain according to the antiSMASH analysis.

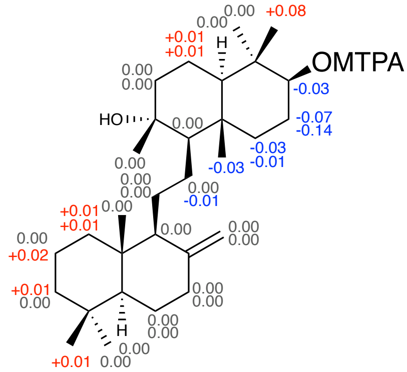

Supplementary Fig. 2. $\Delta\delta^{SR}$ values in ppm for the MTPA esters of homomonoceroid A (**4**).

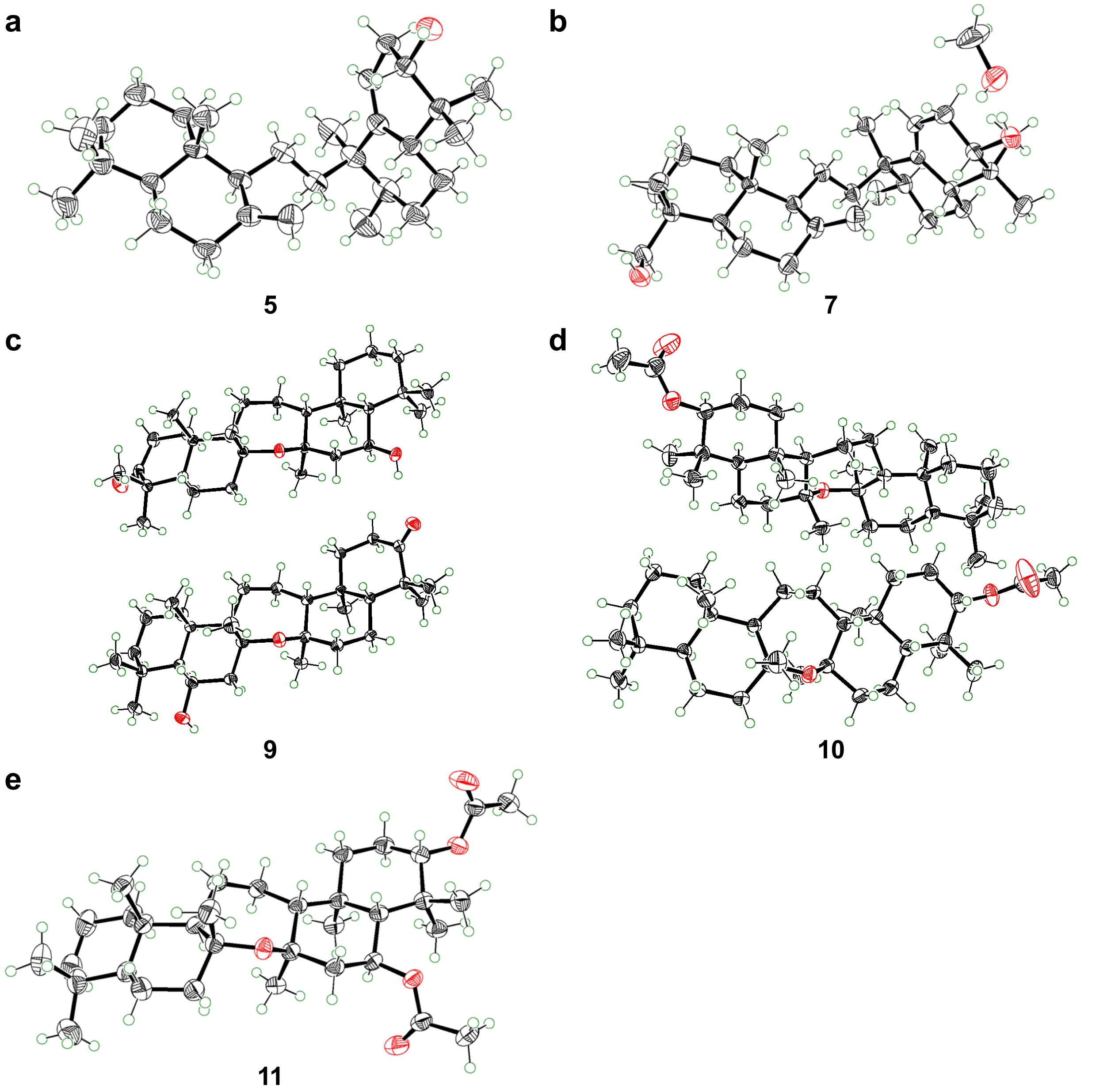

Supplementary Fig. 3. X-ray crystallographic analyses of metabolites obtained in this study. **a**–**e** X-ray crystal structures of compounds **5** (**a**), **7** (**b**), **9** (**c**), **10** (**d**), and **11** (**e**) (with 50% probability of thermal ellipsoid).

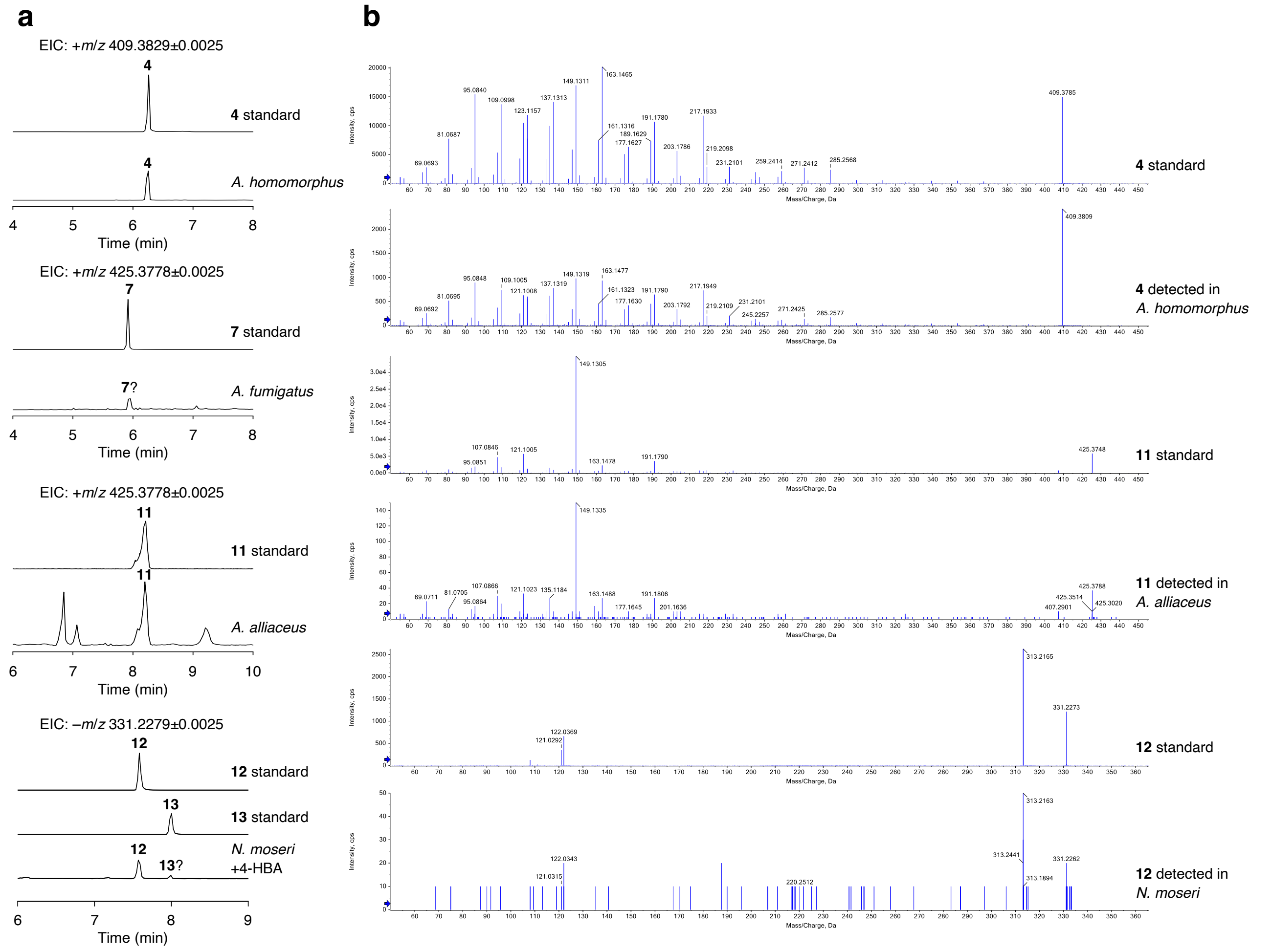

Supplementary Fig. 4. Metabolite analysis of *A. homomorphus* CBS 101889, *A. fumigatus* CBS 144.89, *A. alliaceus* CBS 536.65, and *N. moseri* CBS 164.80. **a** LC–MS profiles of the metabolites from the four fungal strains and the compound standards. **b** MS/MS spectra of **4**, **11**, and **12** detected in the *A. homomorphus*, *A. alliaceus*, and *N. moseri*, respectively, and their standards. The MS/MS spectra of **7** and **13** could not be obtained due to their low productivity. *A. homomorphus* CBS 101889, *A. alliaceus* CBS 536.65, and *N. moseri* CBS 164.80 were cultivated in YES, DPY, MEB (supplemented with 4-HBA) liquid media, respectively, whereas *A. fumigatus* CBS 144.89 was cultivated on PDA plate.

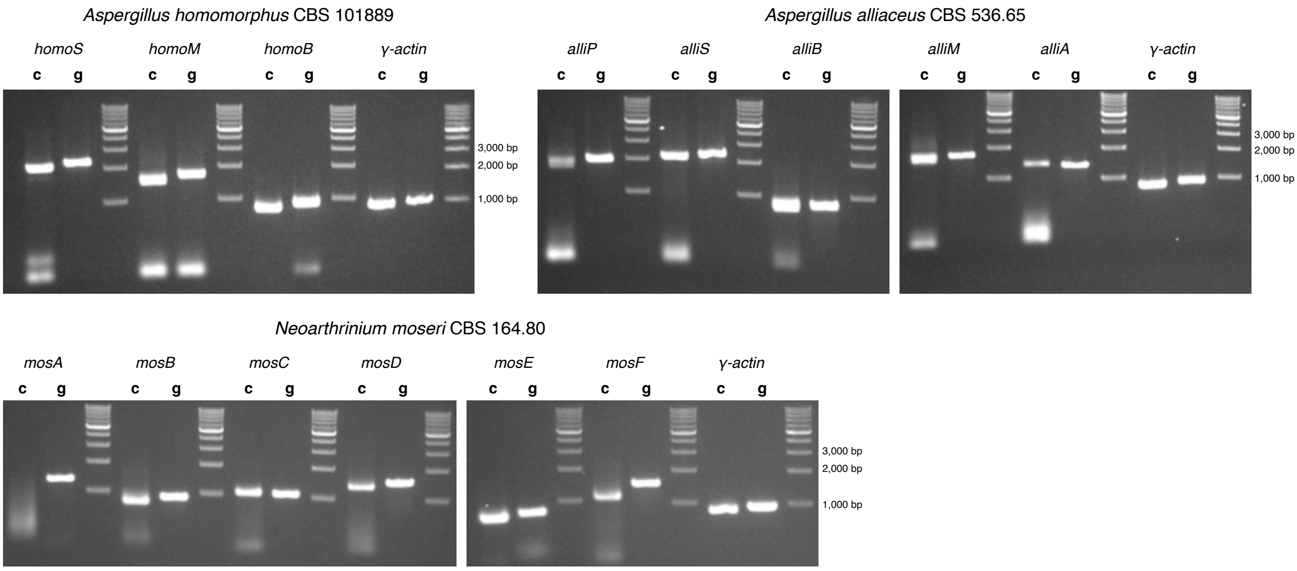

Supplementary Fig. 5. Gene expression analysis of *A. homomorphus* CBS 101889, *A. alliaceus* CBS 536.65, and *N. moseri* CBS 164.80. **c**: complementary DNA (cDNA); **g**: genomic DNA (gDNA). γ-actin was used as a positive control. Source data are provided as a Source Data file.

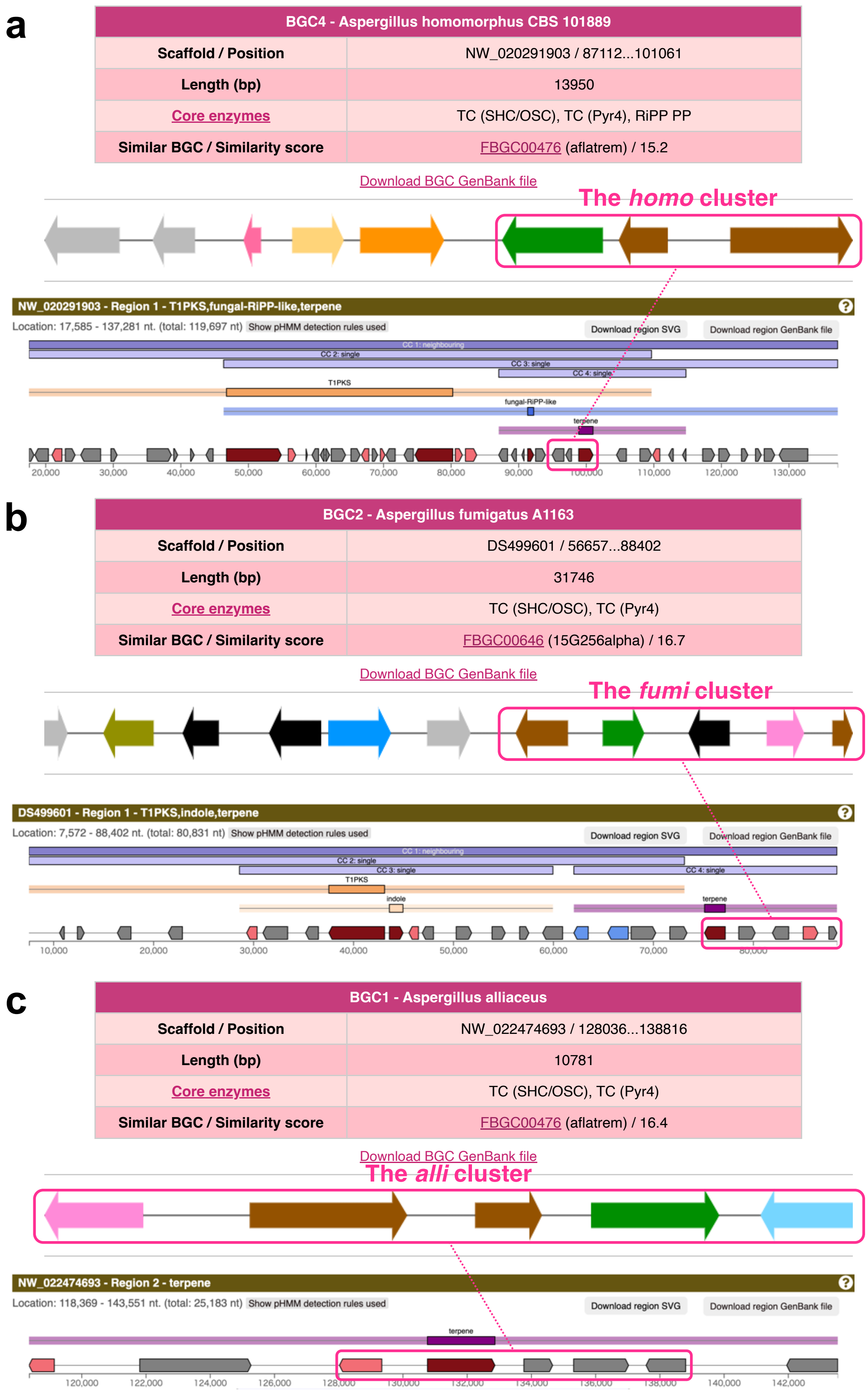

Supplementary Fig. 6. Comparison of BGC extractions by FunBGCeX and antiSMASH. **a** The *homo* cluster from *Aspergillus homomorphus* CBS 101889. **b** The *fumi* cluster from *Aspergillus fumigatus* A1163. **c** The *alli* cluster from *Aspergillus alliaceus* CBS 536.65.

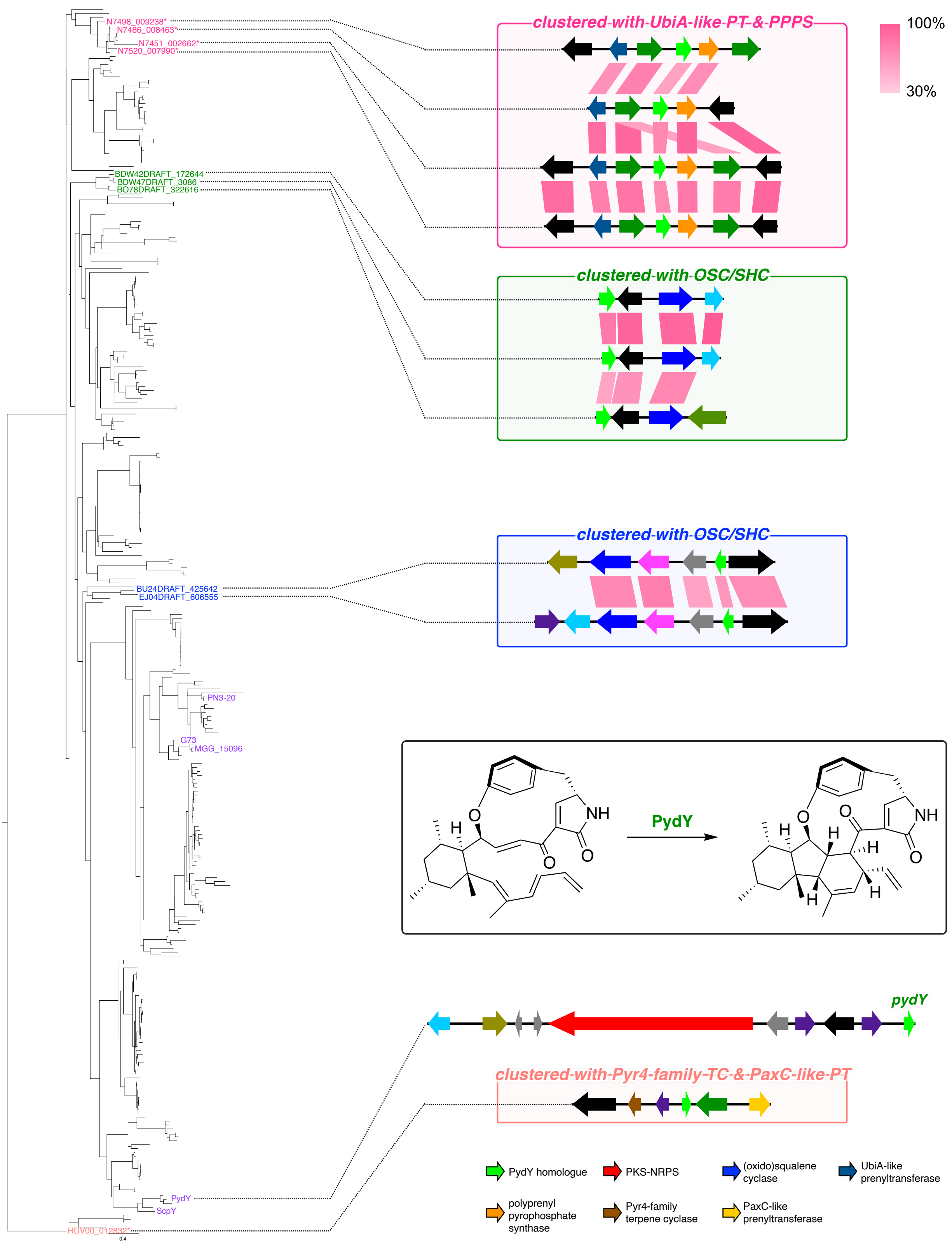

Supplementary Fig. 7. Phylogenetic analysis of PydY homologues, along with their associated BGCs, as well as the reaction catalyzed by PydY. Asterisks indicate proteins not detected by antiSMASH analysis. PT: prenyltransferase; PPPS: polyprenyl pyrophosphate synthase; TC: terpene cyclase.

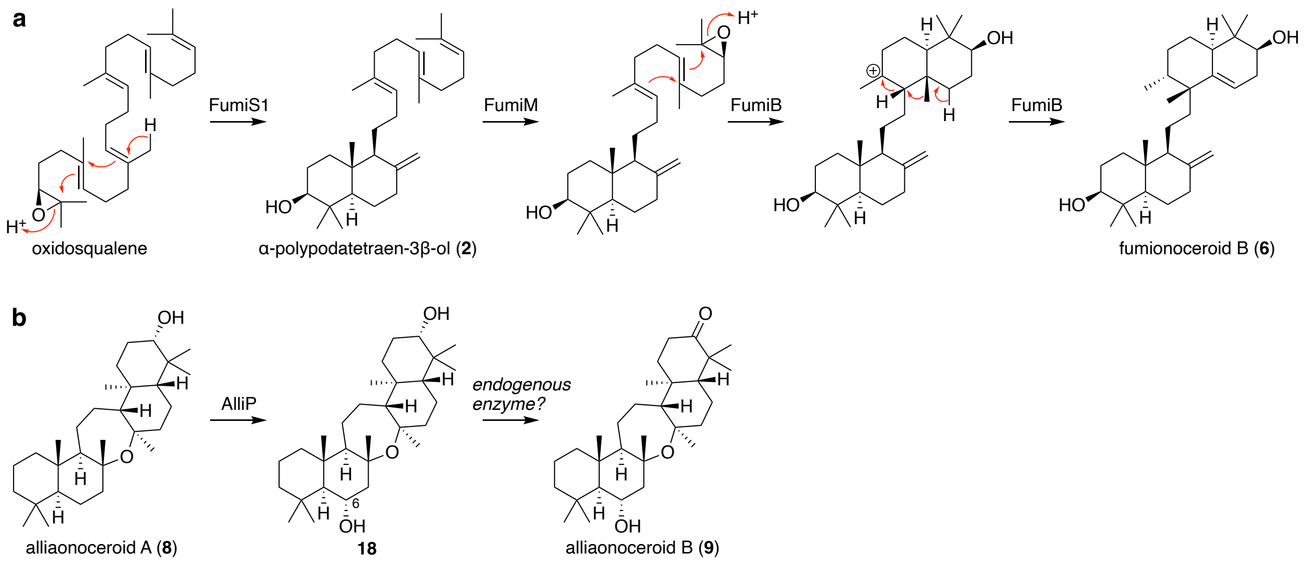

Supplementary Fig. 8. Biosynthesis of fumionoceroid B (**6**) and alliaonoceroid B (**9**). **a**,**b** Predicted biosynthetic pathways leading to **6** (**a**) and **9** (**b**). For the biosynthesis of **9**, the alcohol dehydrogenation by a putative endogenous enzyme might precede the AlliP-catalyzed hydroxylation.

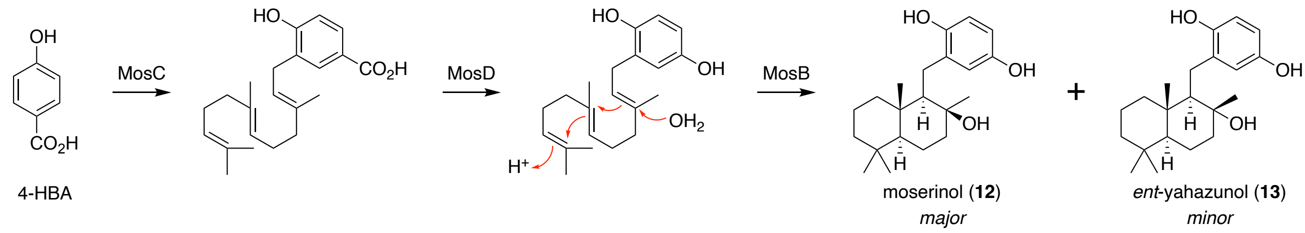

Supplementary Fig. 9. Predicted biosynthetic pathway leading to moserinol (**12**) and (B) *ent*-yahazunol (**13**).

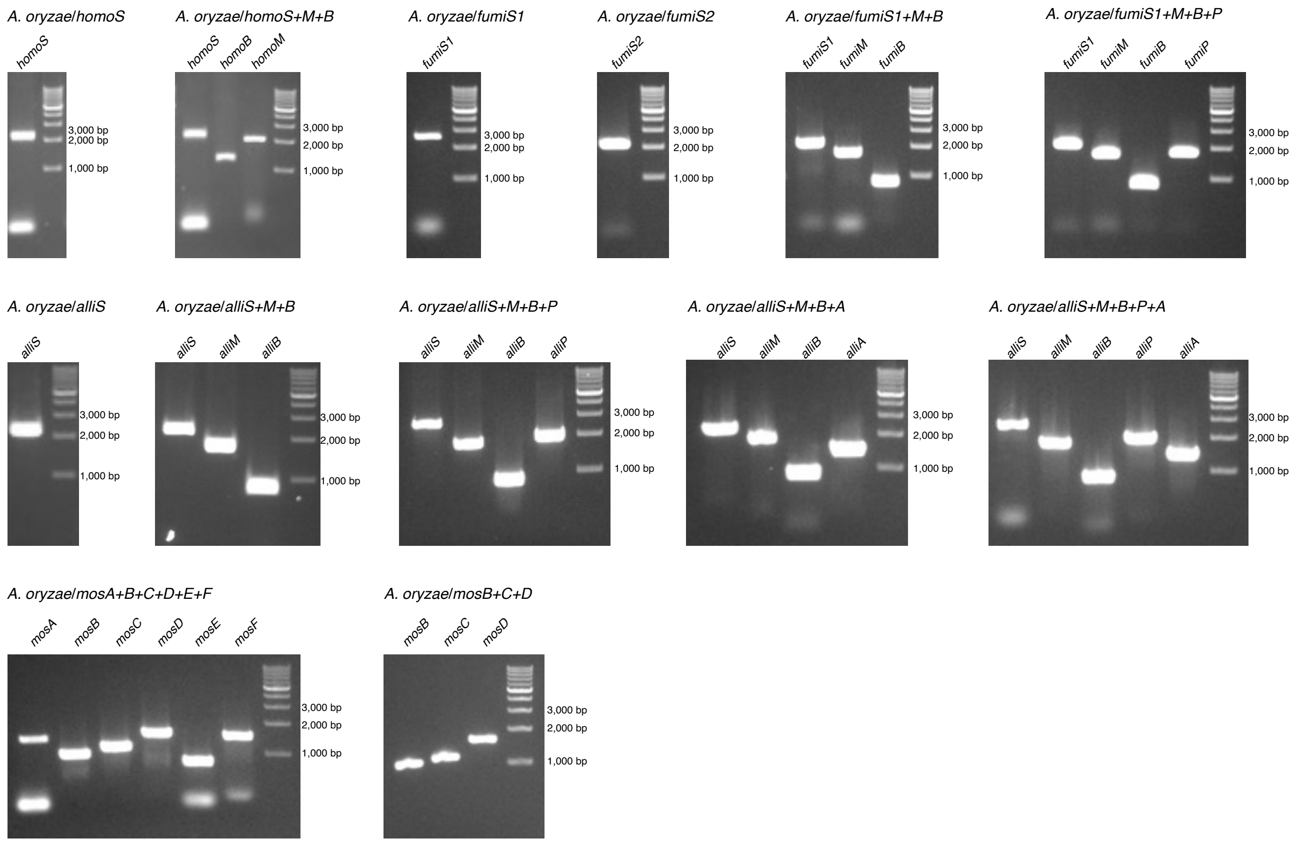

Supplementary Fig. 10. Result of diagnostic PCR for the confirmation of the gene introductions to each *A. oryzae* transformant. Source data are provided as a Source Data file.

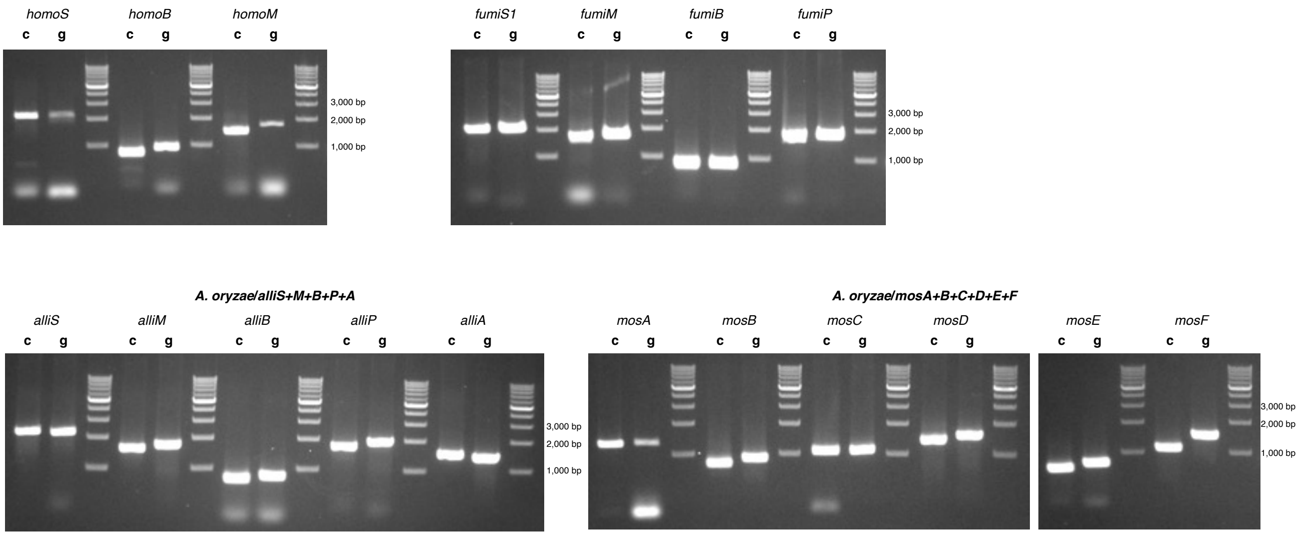

Supplementary Fig. 11. Result of diagnostic PCR for the confirmation of the expression of the biosynthetic genes in selected *A. oryzae* transformants. **c**: complementary DNA (cDNA); **g**: genomic DNA (gDNA). Source data are provided as a Source Data file.

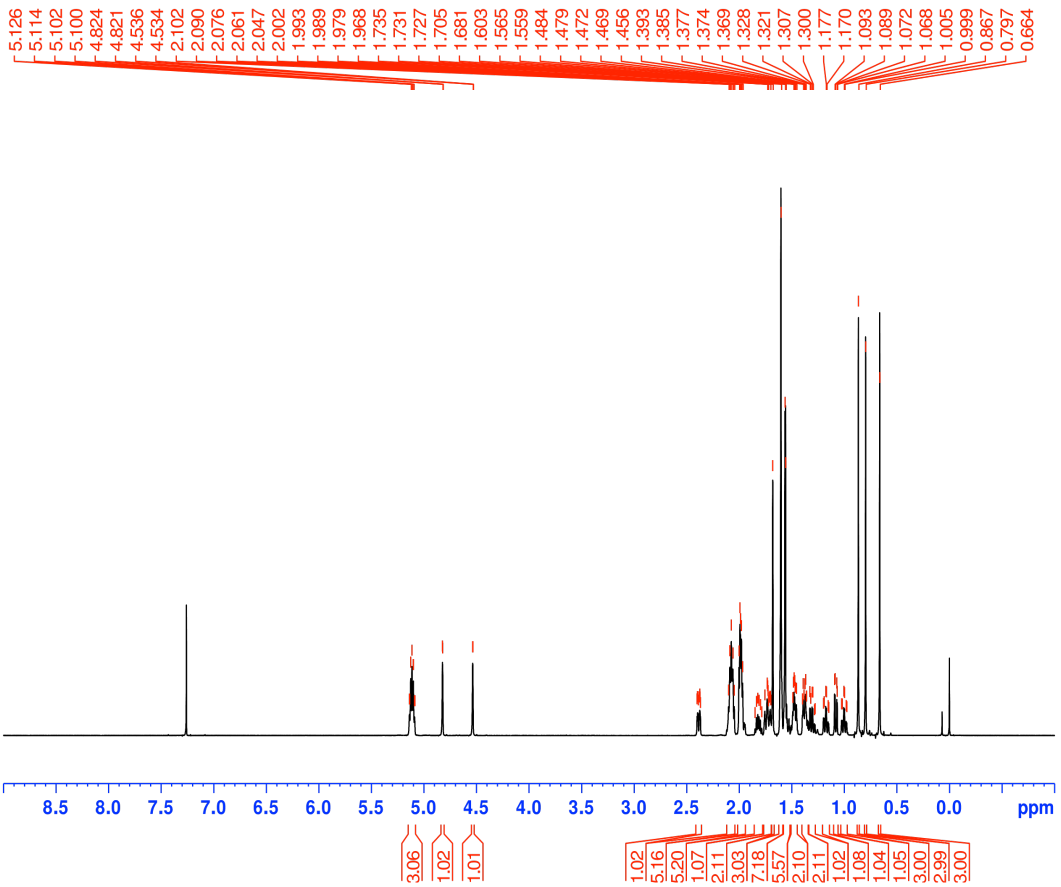

Supplementary Fig. 12. ^{1}H NMR spectrum of **1** in $CDCl_3$ at 600 MHz.

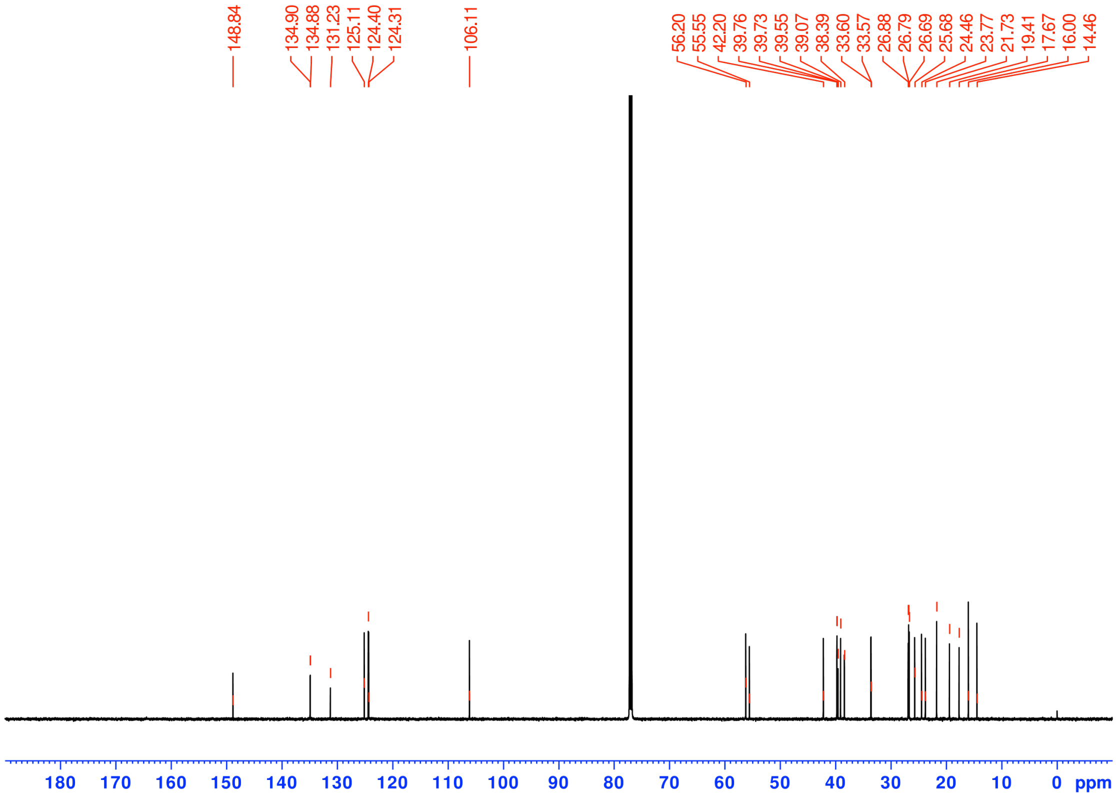

Supplementary Fig. 13. ^{13}C NMR spectrum of **1** in $CDCl_3$ at 150 MHz.

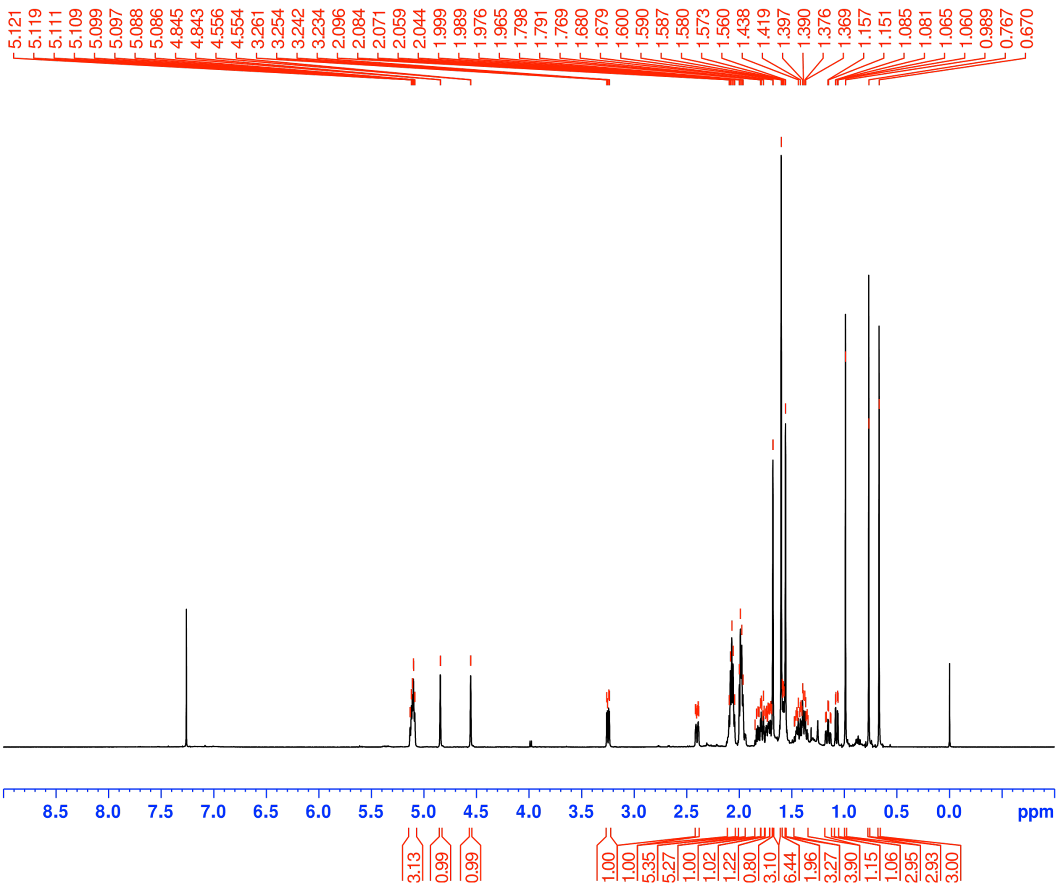

Supplementary Fig. 14. ^{1}H NMR spectrum of **2** in $CDCl_3$ at 600 MHz.

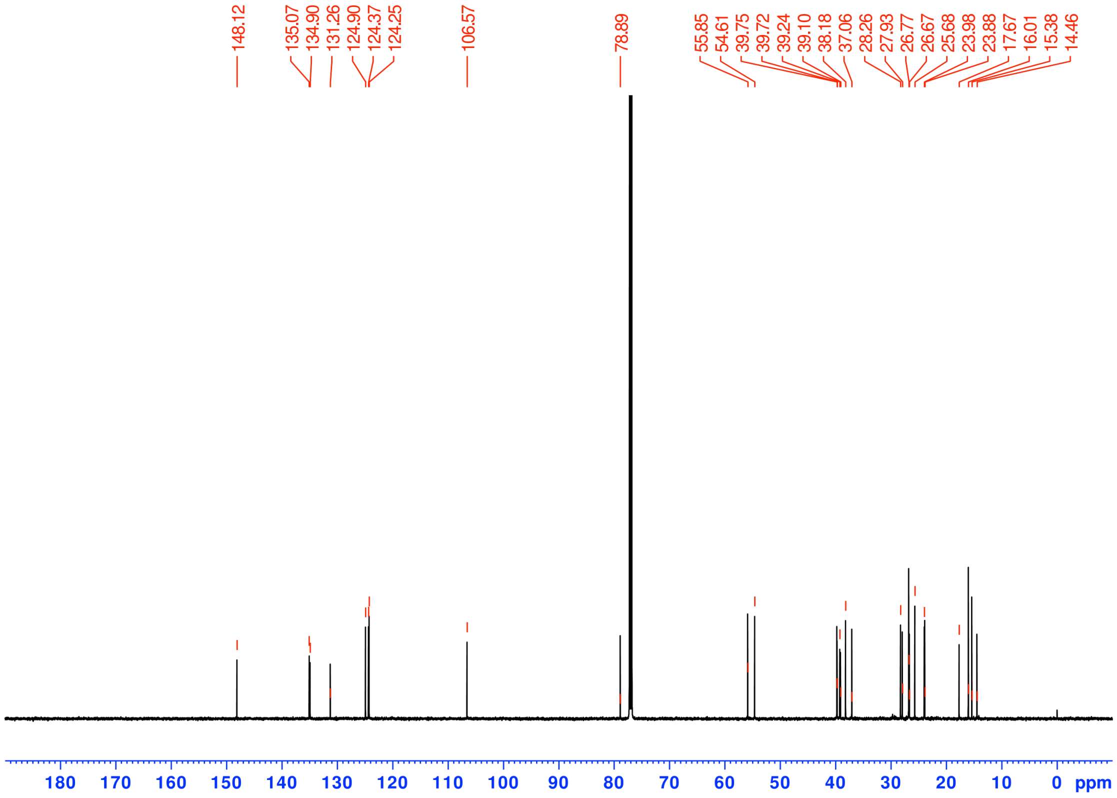

Supplementary Fig. 15. ^{13}C NMR spectrum of **2** in $CDCl_3$ at 150 MHz.

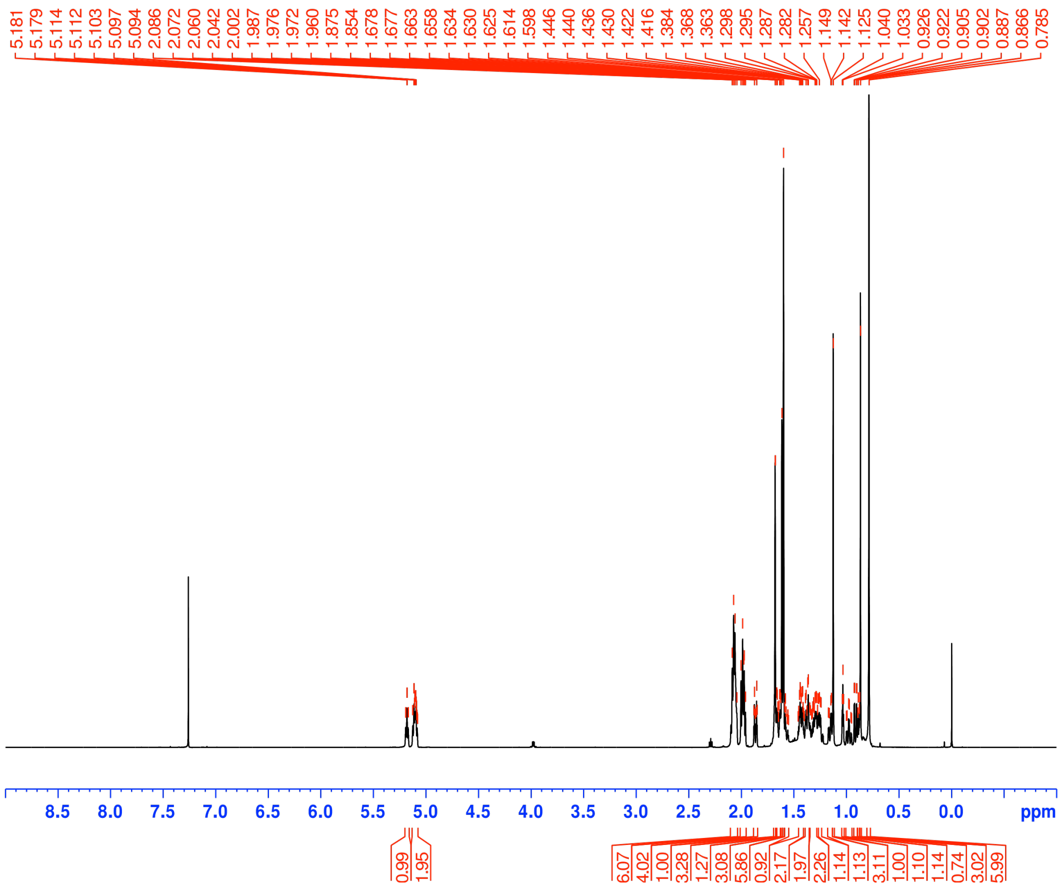

Supplementary Fig. 16. ^{1}H NMR spectrum of **3** in $CDCl_3$ at 600 MHz.

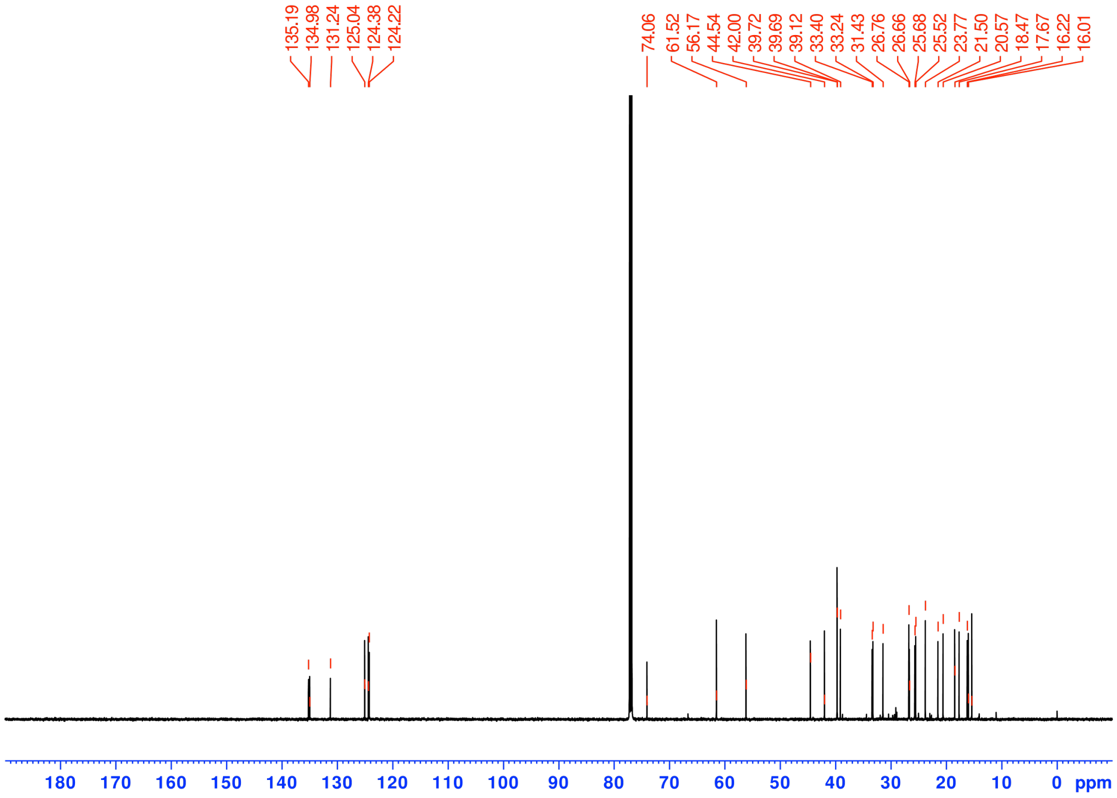

Supplementary Fig. 17. ^{13}C NMR spectrum of **3** in $CDCl_3$ at 150 MHz.

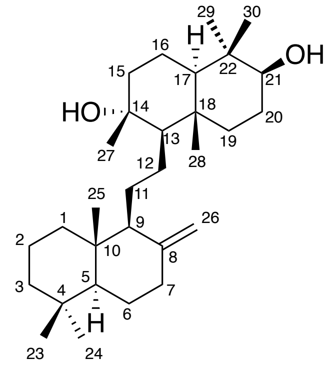

4

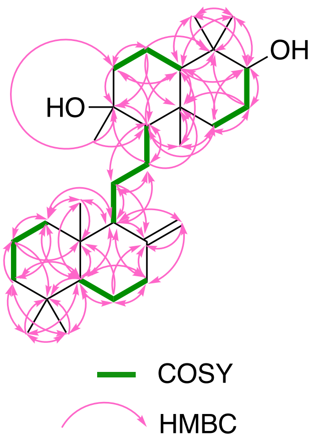

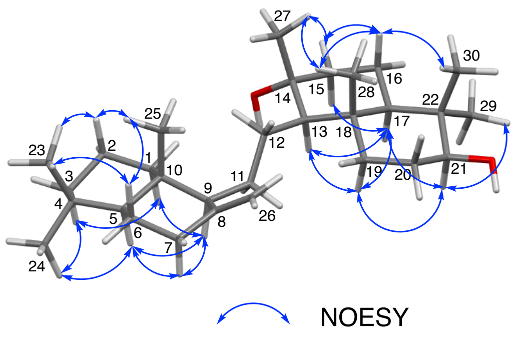

position	δC, type	δH, mult. (*J* in Hz)
1	39.4, CH_2	1.01 (α), m
		1.85 (β), dt (13.0, 4.0)
2	19.9, CH_2	1.48 (α), ddd (14.8, 6.7, 3.4)
		1.61 (β), m
3	42.6, CH_2	1.19 (α), td (13.0, 3.9)
		1.40 (β), dt (12.8, 3.4)
4	33.8, C	
5	55.8, CH	1.06, dd (12.8, 2.7)
6	24.8, CH_2	1.68 (α), m
		1.35 (β), qd (13.0, 4.4)
7	38.9, CH_2	2.06 (α), td (12.9, 4.9)
		2.46 (β), ddd (12.7, 4.0, 2.4)
8	148.6, C	
9	58.6, CH	1.60, m
10	39.9, C	
11	28.2, CH_2	1.69, m
		1.55, td (12.8, 4.8)
12	25.7, CH_2	1.83, m
		0.94, m
13	62.4, CH	0.85, m
14	73.7, C	
15	45.0, CH_2	1.21 (α), ddd (14.3, 10.6, 3.7)
		1.67 (β), m
16	20.6, CH_2	1.45 (α), m
		1.09 (β), qd (13.4, 3.0)
17	55.2, CH	0.70, dd (12.2, 2.1)
18	38.9, C	
19	38.0, CH_2	0.97 (α), m
		1.61 (β), m
20	27.7, CH_2	1.45, m
21	78.5, CH	3.04, dd (10.3, 5.9)
22	39.0, C	
23	22.0, CH_3	0.84, s
24	33.9, CH_3	0.90, s
25	15.0, CH_3	0.84, s
26	107.8, CH_2	5.16, brs
		5.09, brd (0.74)
27	24.2, CH_3	1.02, s
28	15.7, CH_3	0.65, s
29	28.4, CH_3	0.99, s
30	15.6, CH_3	0.73, s

^{1}H NMR: 600 MHz, ^{13}C NMR: 150 MHz (in C_6D_6)

Supplementary Fig. 18. NMR data of homomonoceroid A (**4**).

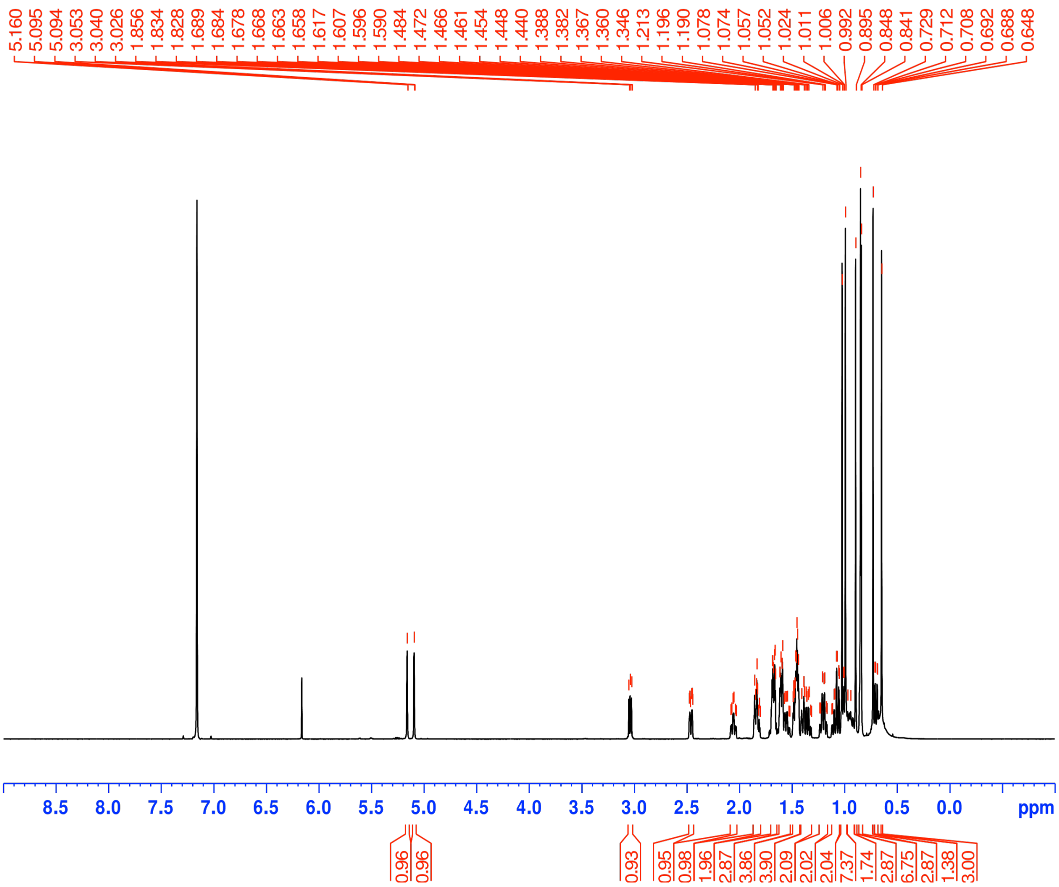

Supplementary Fig. 19. ^{1}H NMR spectrum of **4** in C_6D_6 at 600 MHz.

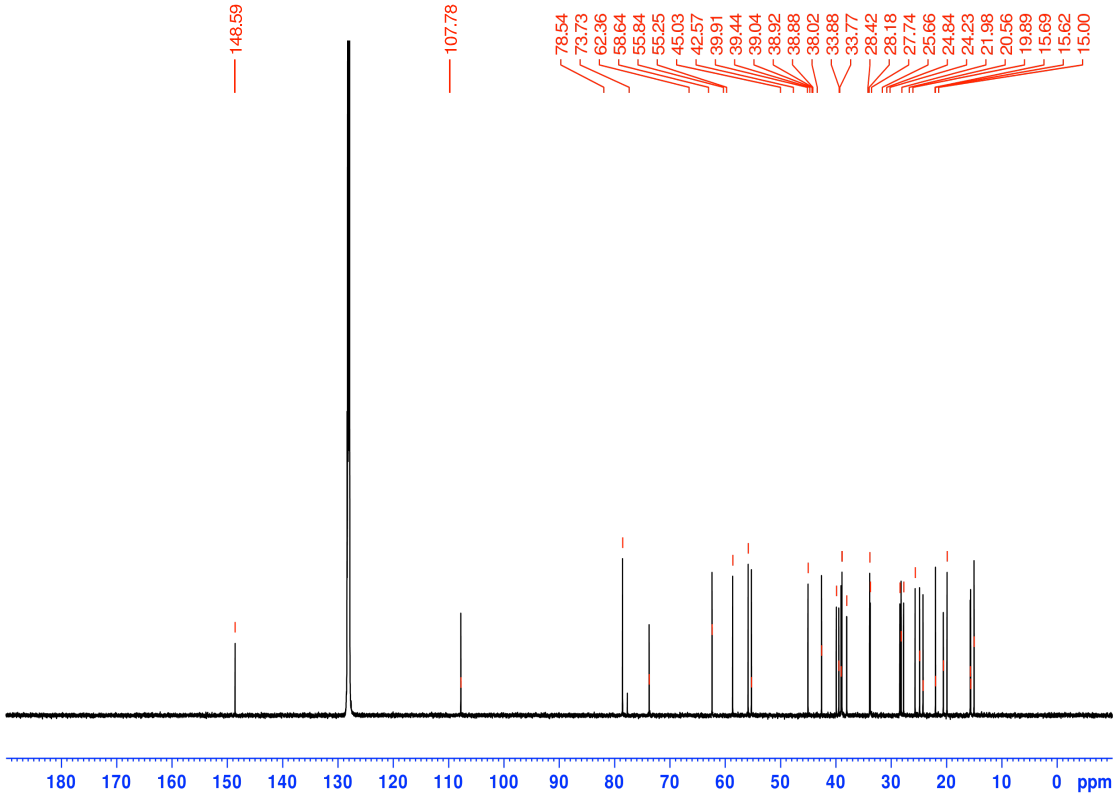

Supplementary Fig. 20. ^{13}C NMR spectrum of **4** in C_6D_6 at 150 MHz.

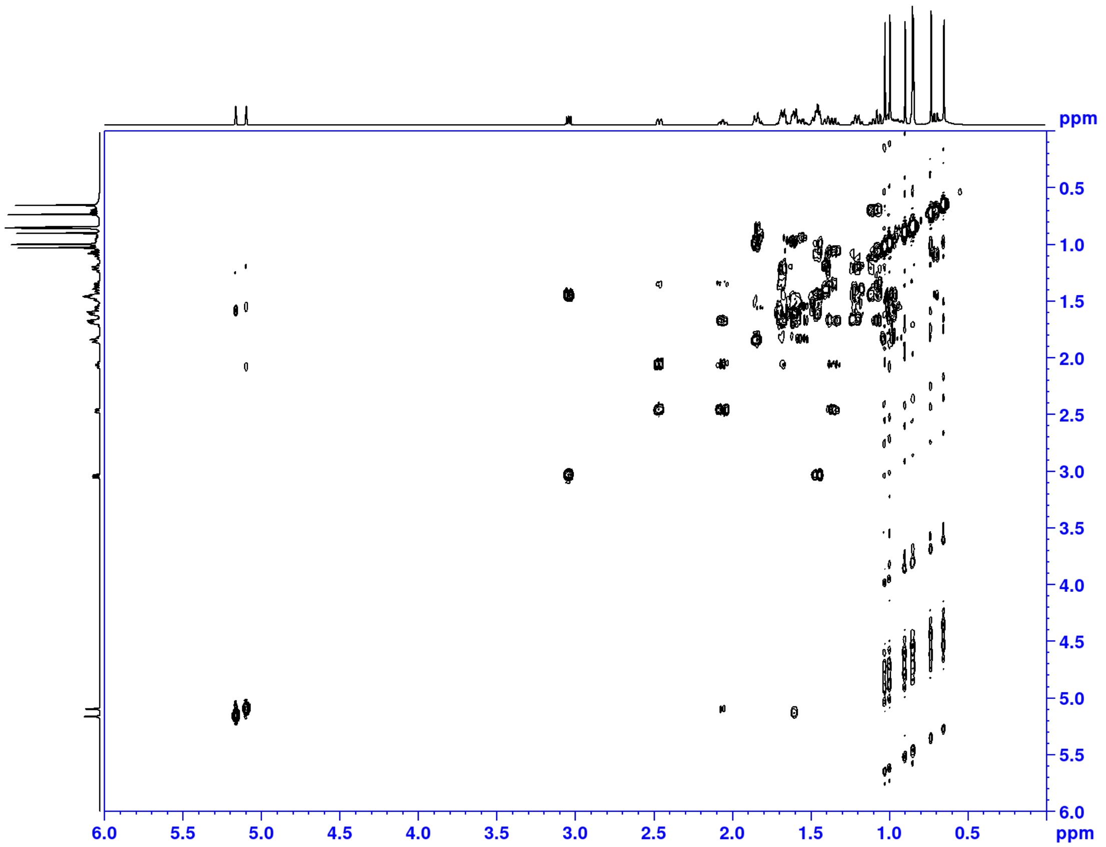

Supplementary Fig. 21. ^{1}H-^{1}H COSY spectrum of **4** in C_6D_6.

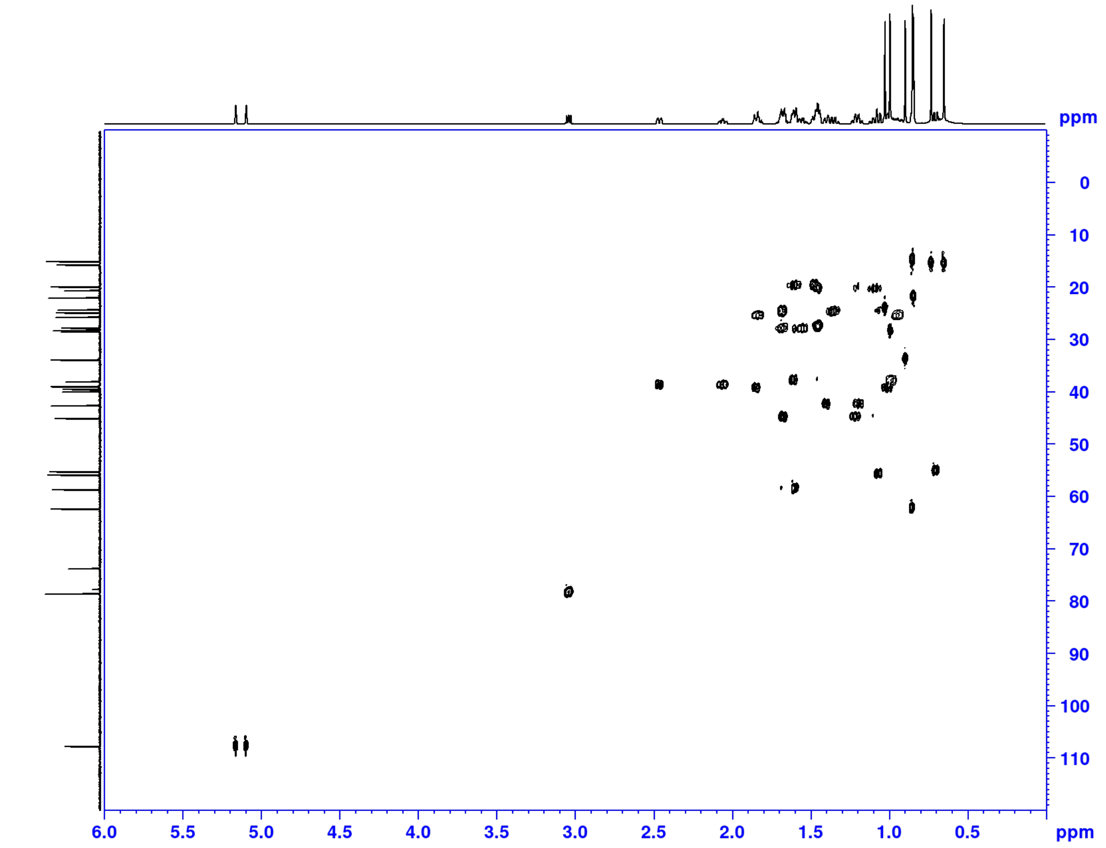

Supplementary Fig. 22. HSQC spectrum of **4** in C_6D_6.

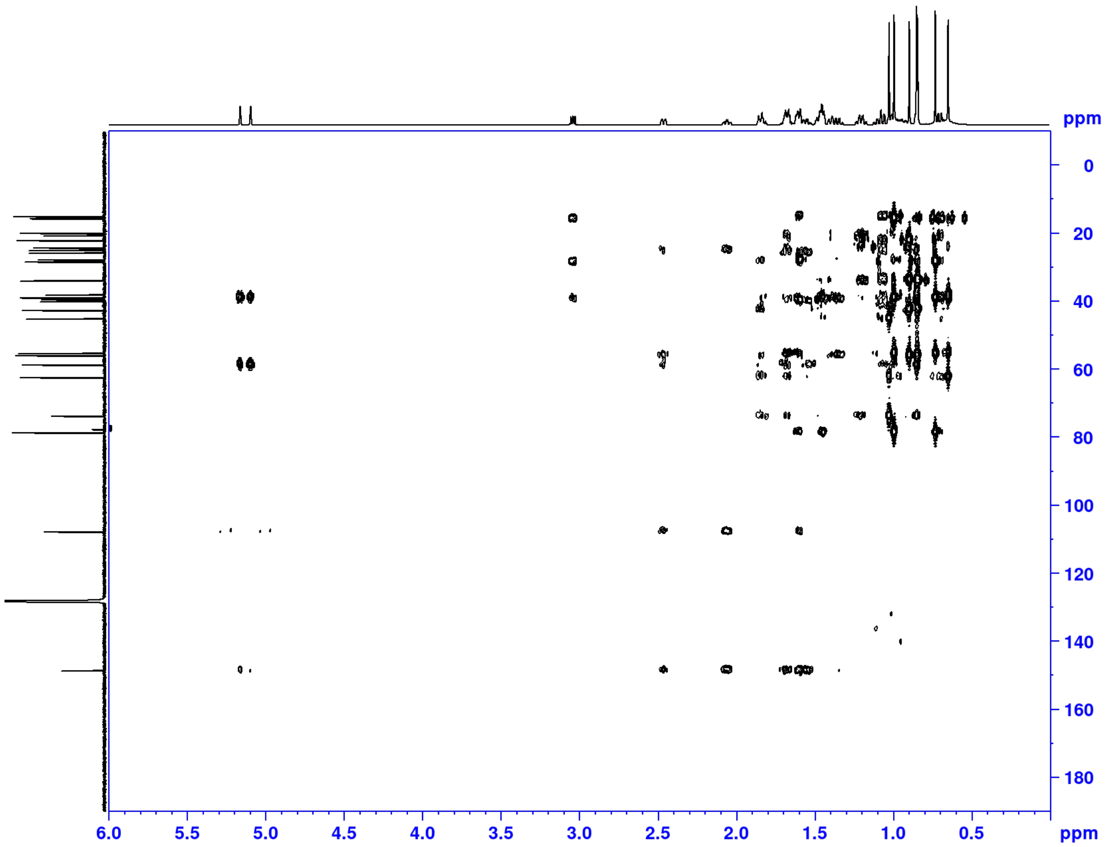

Supplementary Fig. 23. HMBC spectrum of **4** in C_6D_6.

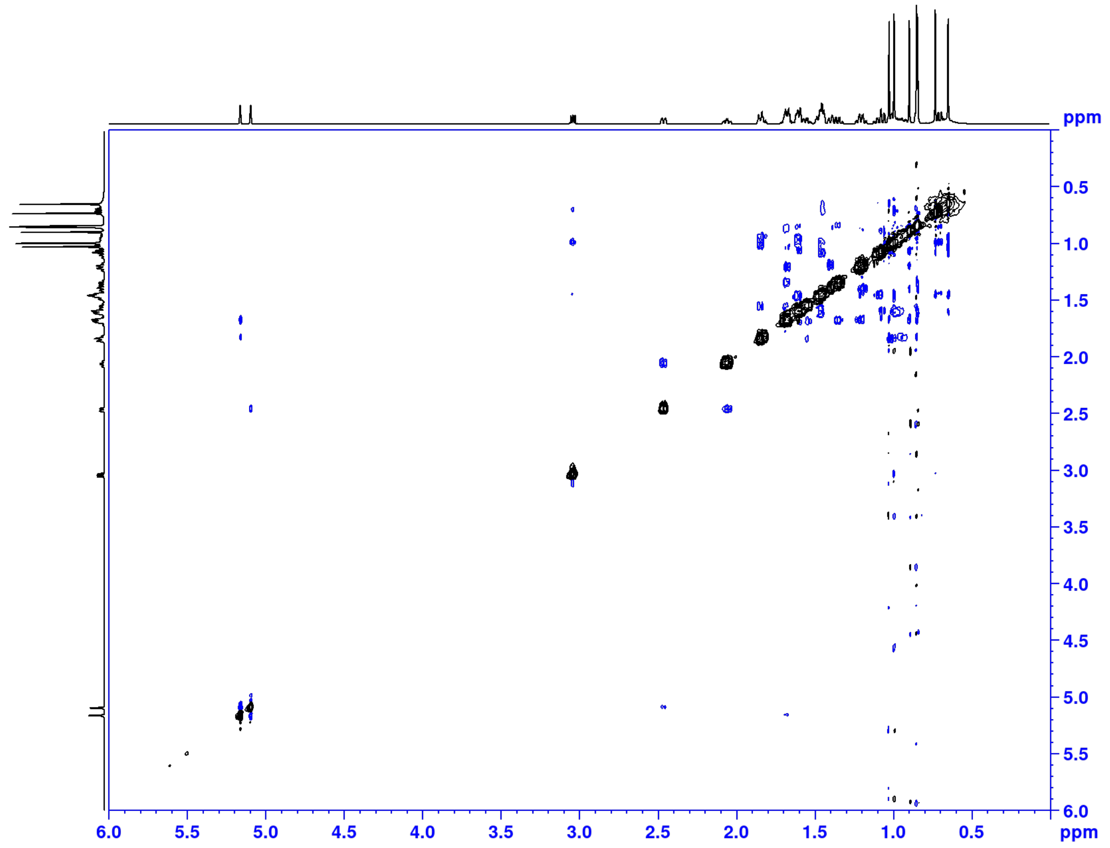

Supplementary Fig. 24. NOESY spectrum of **4** in C_6D_6.

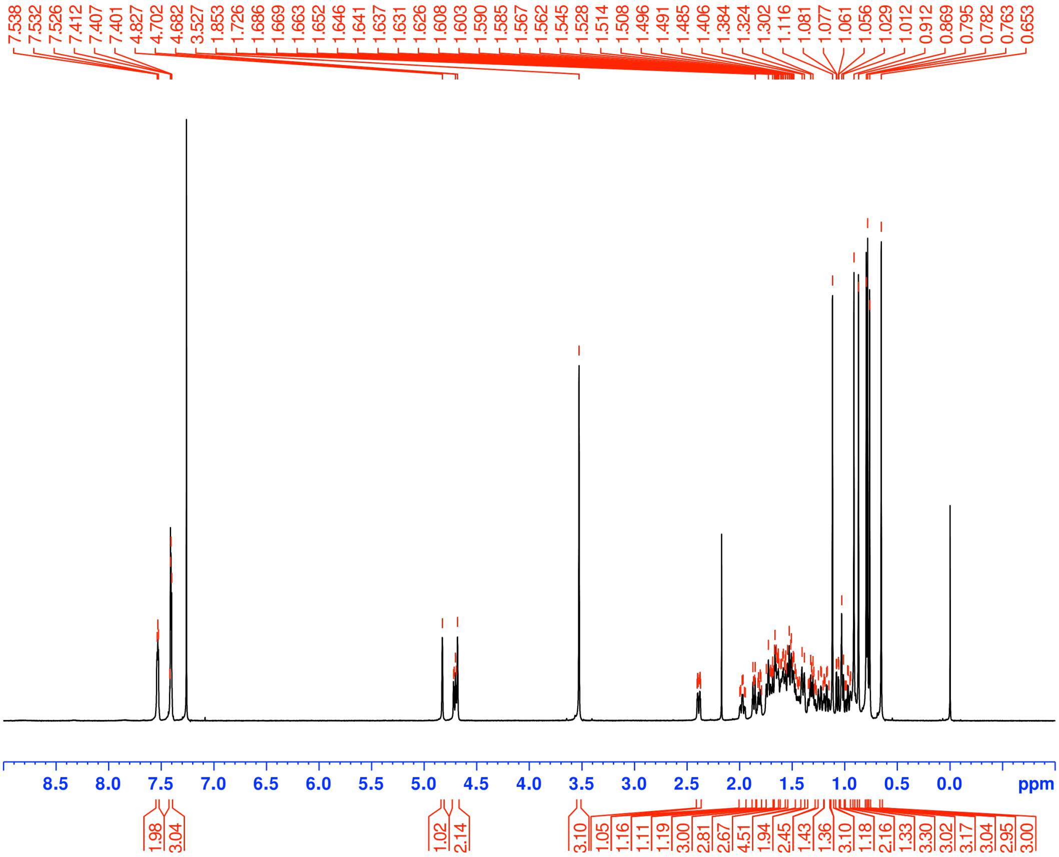

Supplementary Fig. 25. ^{1}H NMR spectrum of (*S*)-MTPA ester of **4** in $CDCl_3$ at 600 MHz.

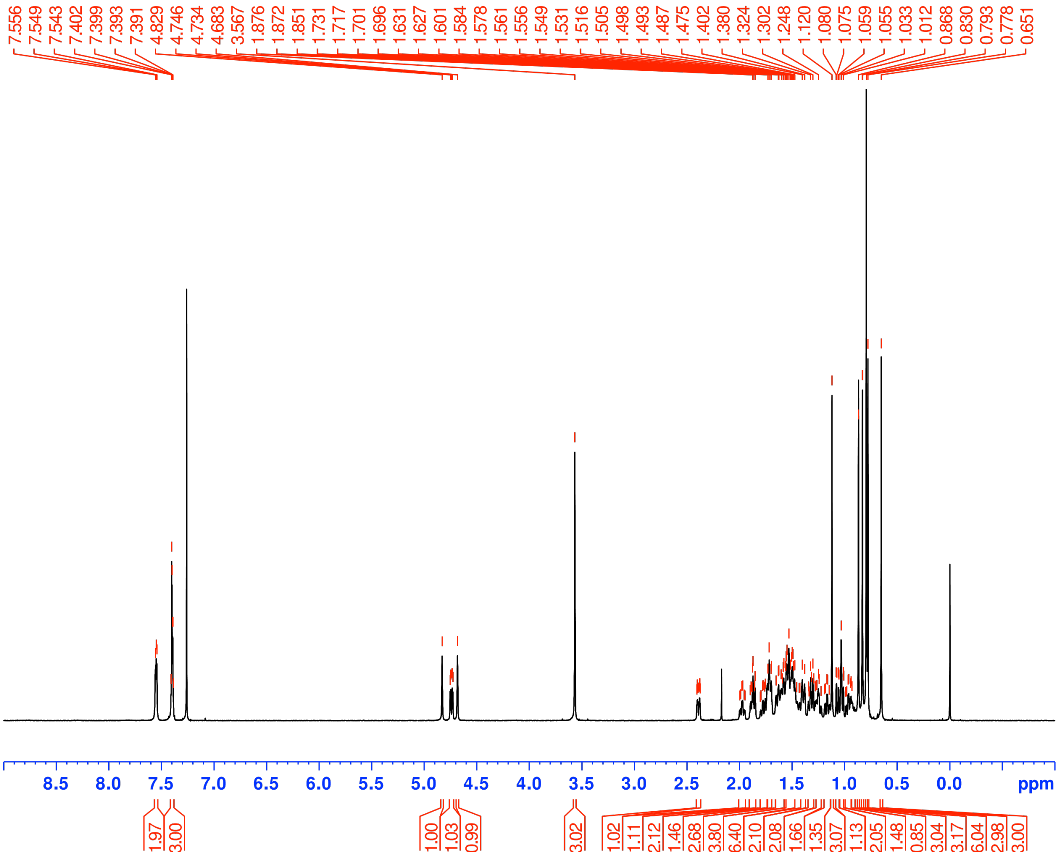

Supplementary Fig. 26. ^{1}H NMR spectrum of (*R*)-MTPA ester of **4** in $CDCl_3$ at 600 MHz.

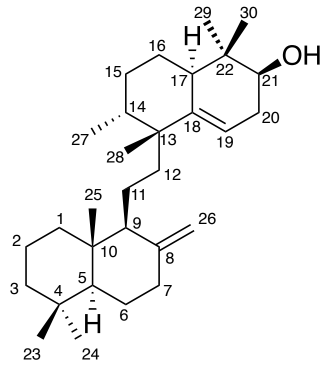

5

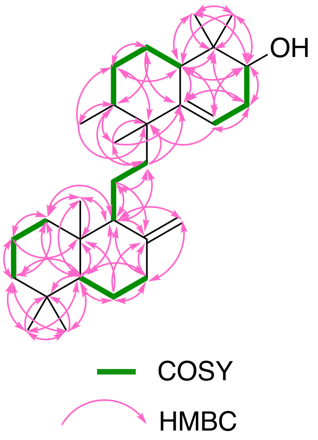

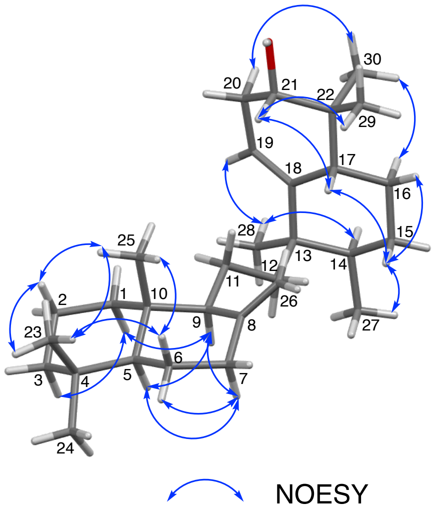

position	δC, type	δH, mult. (*J* in Hz)
1	39.0, CH_2	1.00 (α), td (13.2, 3.9)
		1.73 (β), m
2	19.4, CH_2	1.47 (α), m
		1.56 (β), qt (13.7, 3.3)
3	42.2, CH_2	1.16 (α), td (13.5, 4.0)
		1.38 (β), m
4	33.6, C	
5	55.6, CH	1.07, dd (12.7, 2.7)
6	24.5, CH_2	1.71 (α), m
		1.29 (β), qd (13.0, 4.3)
7	38.5, CH_2	1.97 (α), td (12.8, 5.0)
		2.36 (β), ddd (12.8, 4.0, 2.4)
8	149.6, C	
9	58.1, CH	1.49, brd (10.4)
10	40.0, C	
11	17.4, CH_2	1.20, m
		0.95, m
12	30.2, CH_2	1.70, m
		0.74, td (12.7, 4.0)
13	42.7, C	
14	43.1, CH	1.27, m
15	30.9, CH_2	1.38 (α), m
		1.44 (β), m
16	28.7, CH_2	1.80 (α), m
		1.26 (β), m
17	44.3, CH	1.78, m
18	144.8, C	
19	114.5, CH	5.33, t (3.6)
20	31.4, CH_2	2.33 (α), m
		2.09 (β), m
21	75.0, CH	3.44, dd (7.2, 5.1)
22	36.8, C	
23	21.7, CH_3	0.78, s
24	33.6, CH_3	0.86, s
25	14.5, CH_3	0.61, s
26	105.8, CH_2	4.76, brs
		4.44, brs
27	16.5, CH_3	0.83, d (6.7)
28	22.7, CH_3	1.01, s
29	26.3, CH_3	0.95, s
30	18.2, CH_3	0.84, s

^{1}H NMR: 600 MHz, ^{13}C NMR: 150 MHz (in $CDCl_3$)

Supplementary Fig. 27. NMR data of fumionoceroid A (**5**).

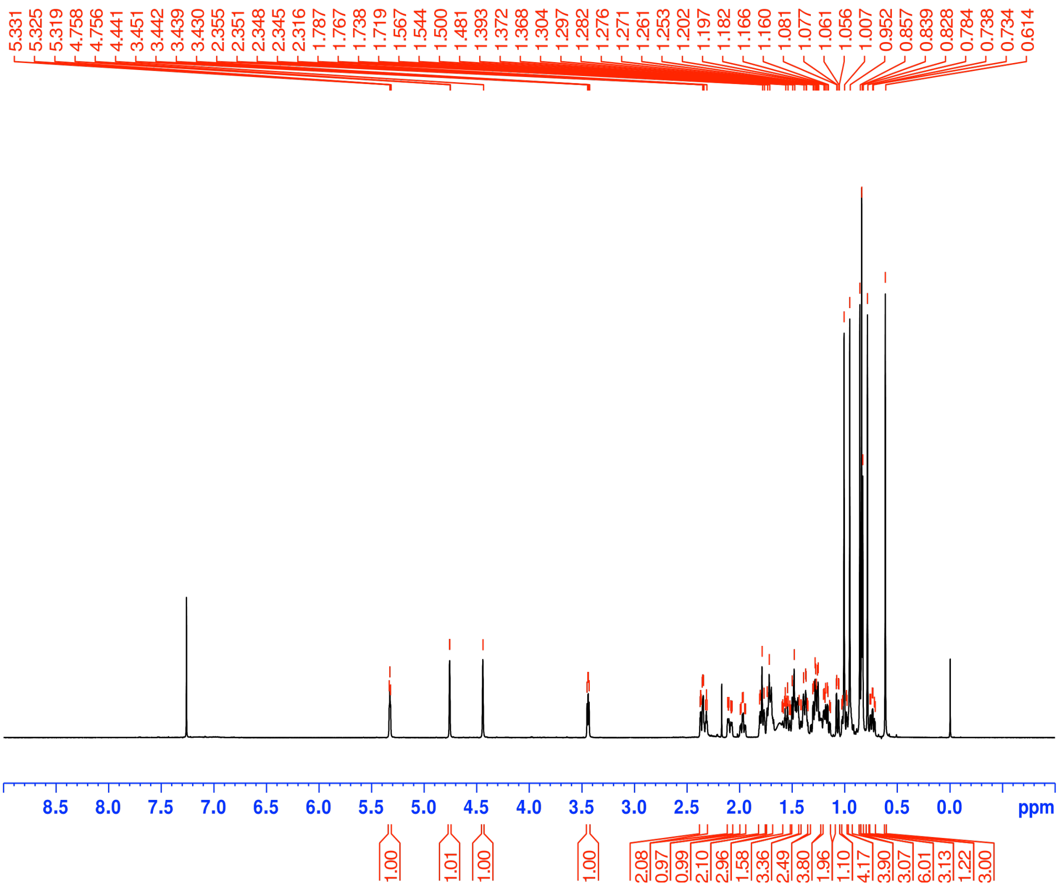

Supplementary Fig. 28. ^{1}H NMR spectrum of **5** in $CDCl_3$ at 600 MHz.

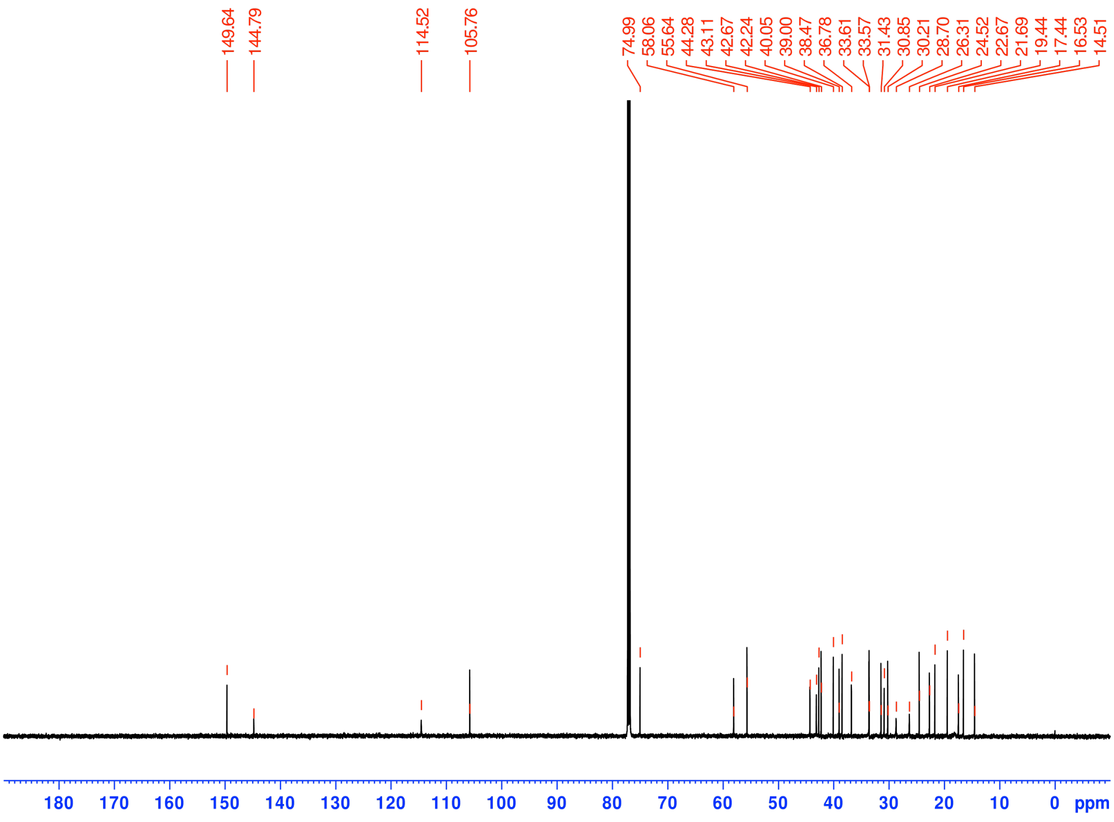

Supplementary Fig. 29. ^{13}C NMR spectrum of **5** in $CDCl_3$ at 150 MHz.

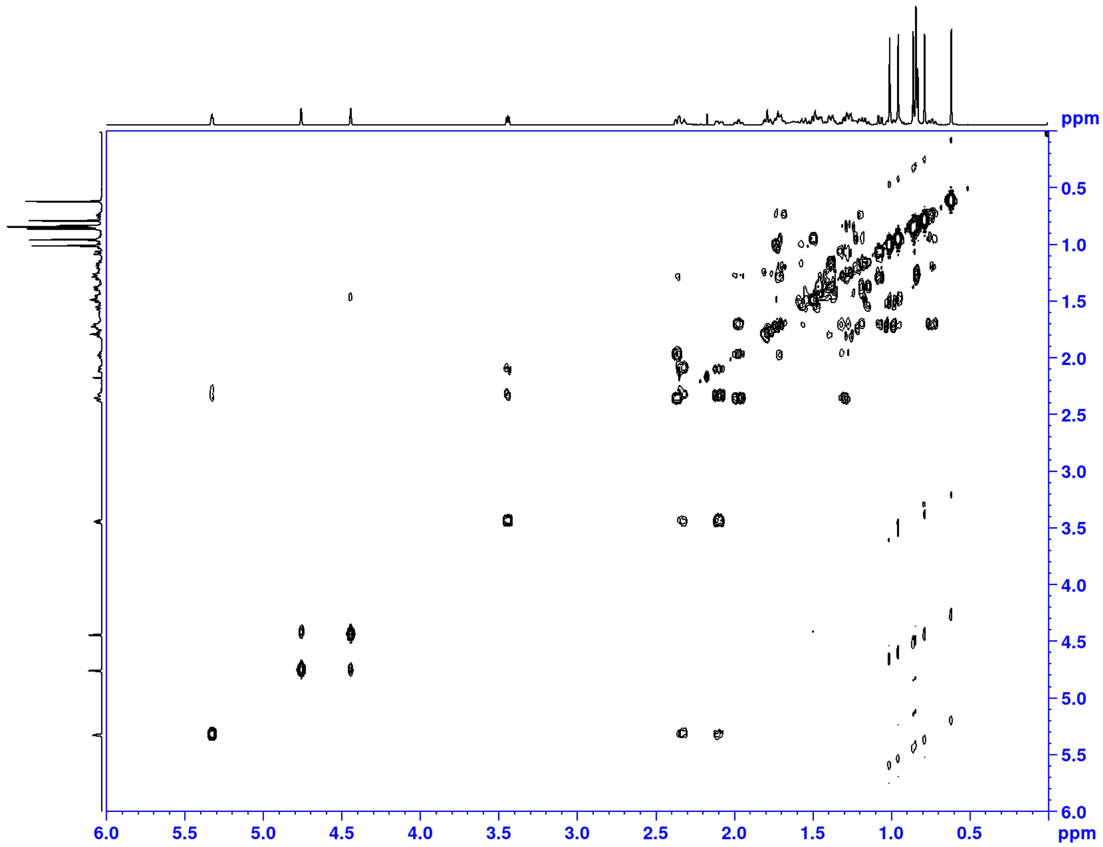

Supplementary Fig. 30. ^{1}H-^{1}H COSY spectrum of **5** in $CDCl_3$.

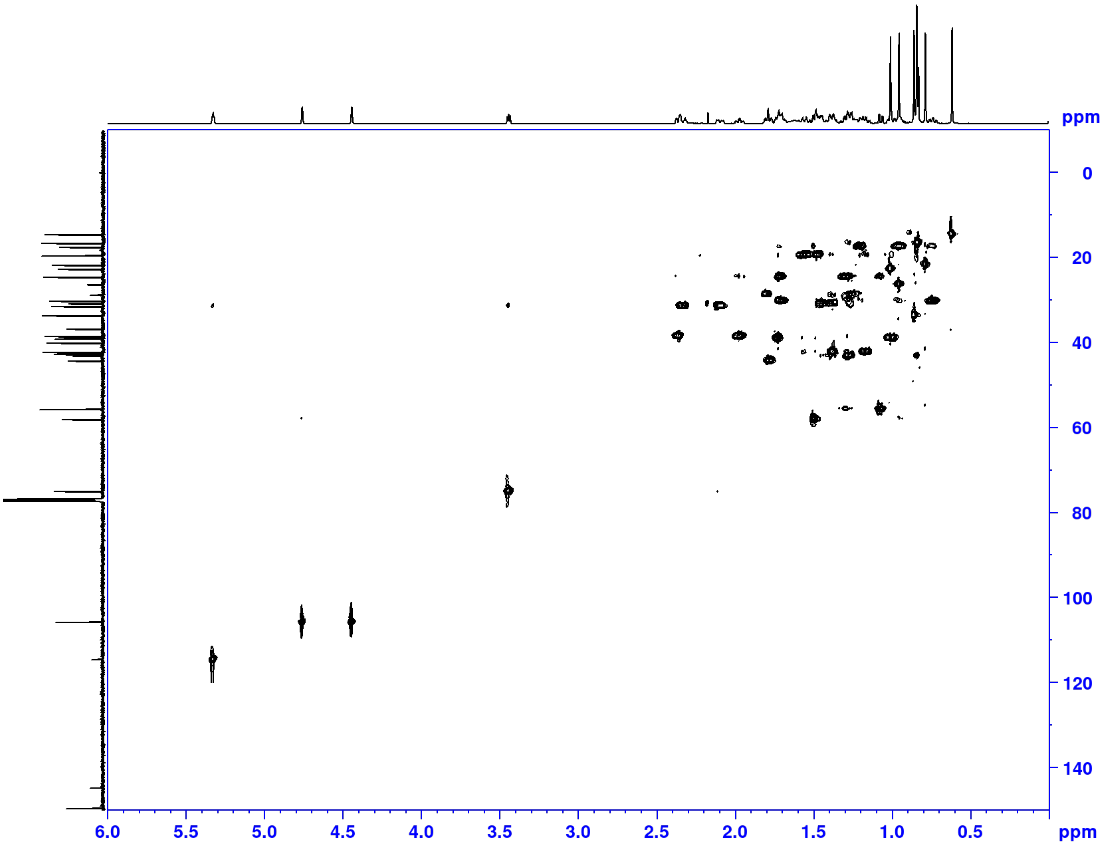

Supplementary Fig. 31. HSQC spectrum of **5** in $CDCl_3$.

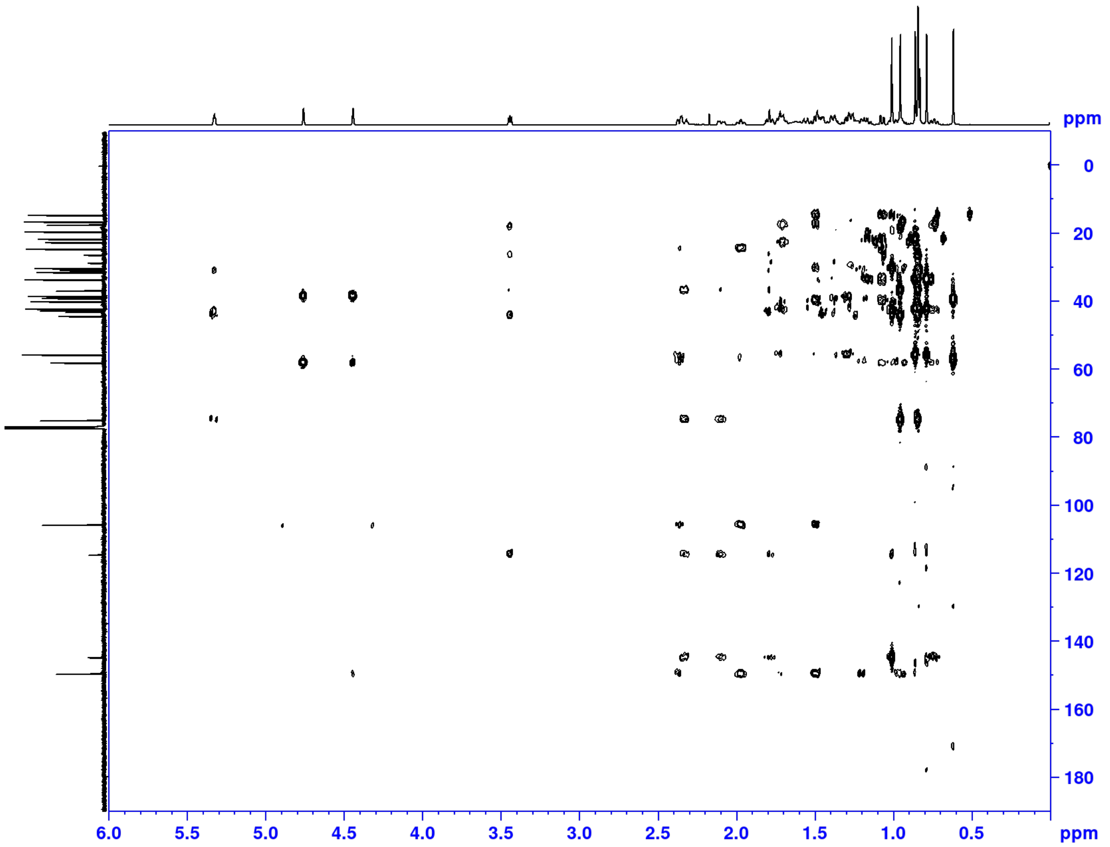

Supplementary Fig. 32. HMBC spectrum of **5** in $CDCl_3$.

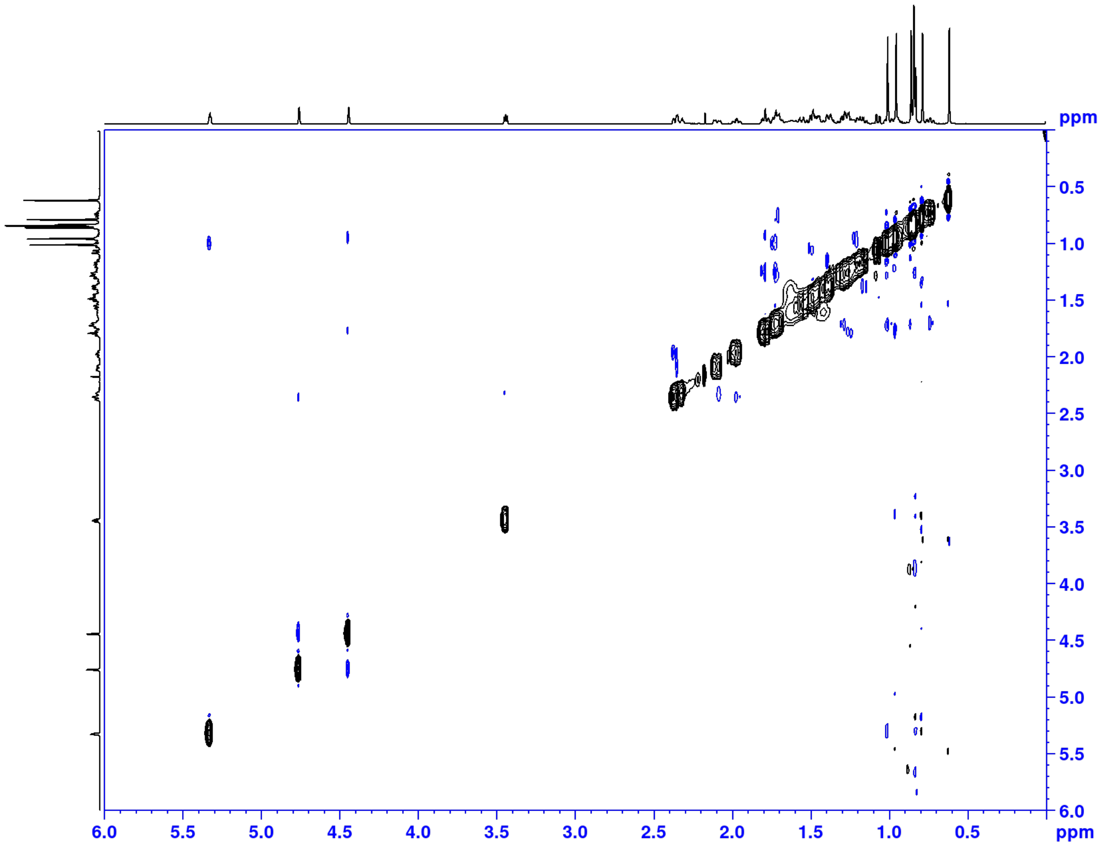

Supplementary Fig. 33. NOESY spectrum of **5** in $CDCl_3$.

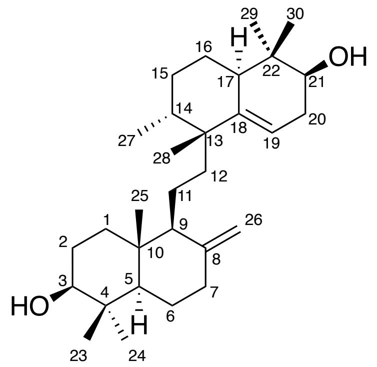

6

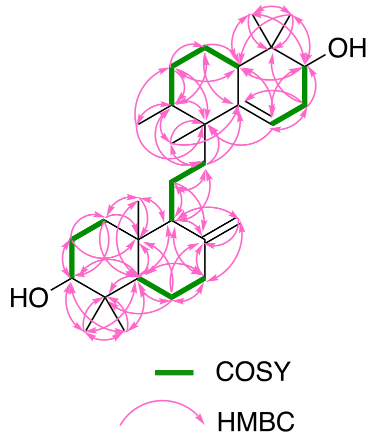

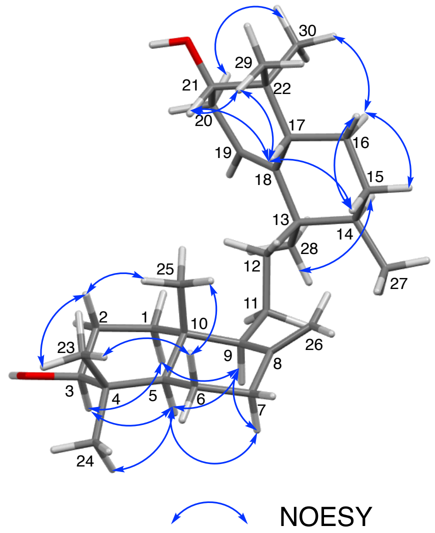

position	δ_C, type	δ_H, mult. (*J* in Hz)
1	37.0, CH_2	1.16 (α), m
		1.77 (β), m
2	28.0, CH_2	1.70 (α), m
		1.58 (β), m
3	78.9, CH	3.24, dd (11.8, 3.8)
4	39.1, C	
5	54.7, CH	1.06, brd (12.5)
6	24.0, CH_2	1.73 (α), m
		1.36 (β), qd (12.7, 3.6)
7	38.3, CH_2	1.97 (α), td (12.8, 4.5)
		2.38 (β), brd (13.0)
8	148.9, C	
9	57.7, CH	1.46, brd (10.2)
10	39.7, C	
11	17.6, CH_2	1.17, m
		0.97, m
12	30.1, CH_2	1.71, m
		0.73, td (11.8, 3.0)
13	42.6, C	
14	43.1, CH	1.27, m
15	30.8, CH_2	1.38 (α), m
		1.46 (β), m
16	28.7, CH_2	1.79 (α), m
		1.26 (β), m
17	44.3, CH	1.77, m
18	144.7, C	
19	114.6, CH	5.32, brs
20	31.4, CH_2	2.33 (α), m
		2.09 (β), m
21	75.0, CH	3.44, brt (5.5)
22	36.8, C	
23	15.3, CH_3	0.76, s
24	28.3, CH_3	0.98, s
25	14.5, CH_3	0.62, s
26	106.2, CH_2	4.78, brs
		4.46, brs
27	16.5, CH_3	0.83, d (7.3)
28	22.7, CH_3	1.00, s
29	26.3, CH_3	0.95, s
30	18.2, CH_3	0.84, s

^1H NMR: 600 MHz, ^{13}C NMR: 150 MHz (in $CDCl_3$)

Supplementary Fig. 34. NMR data of fumionoceroid B (**6**).

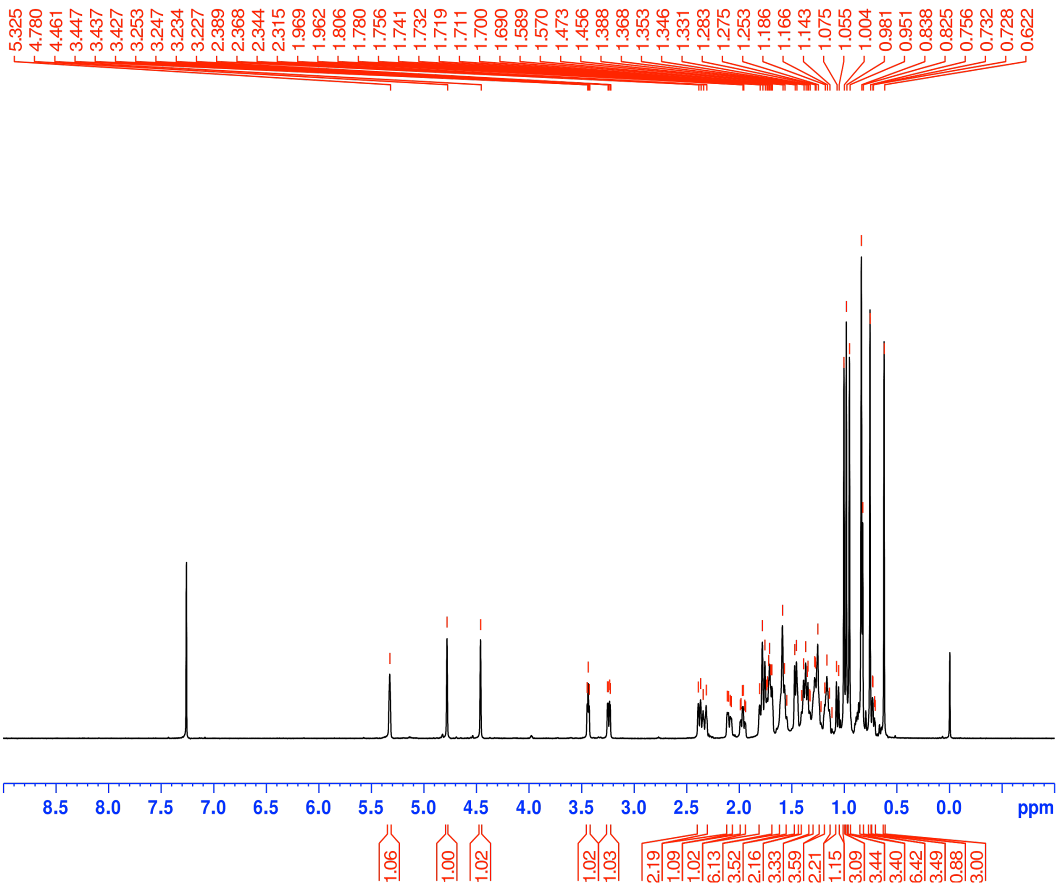

Supplementary Fig. 35. ^{1}H NMR spectrum of **6** in $CDCl_3$ at 600 MHz.

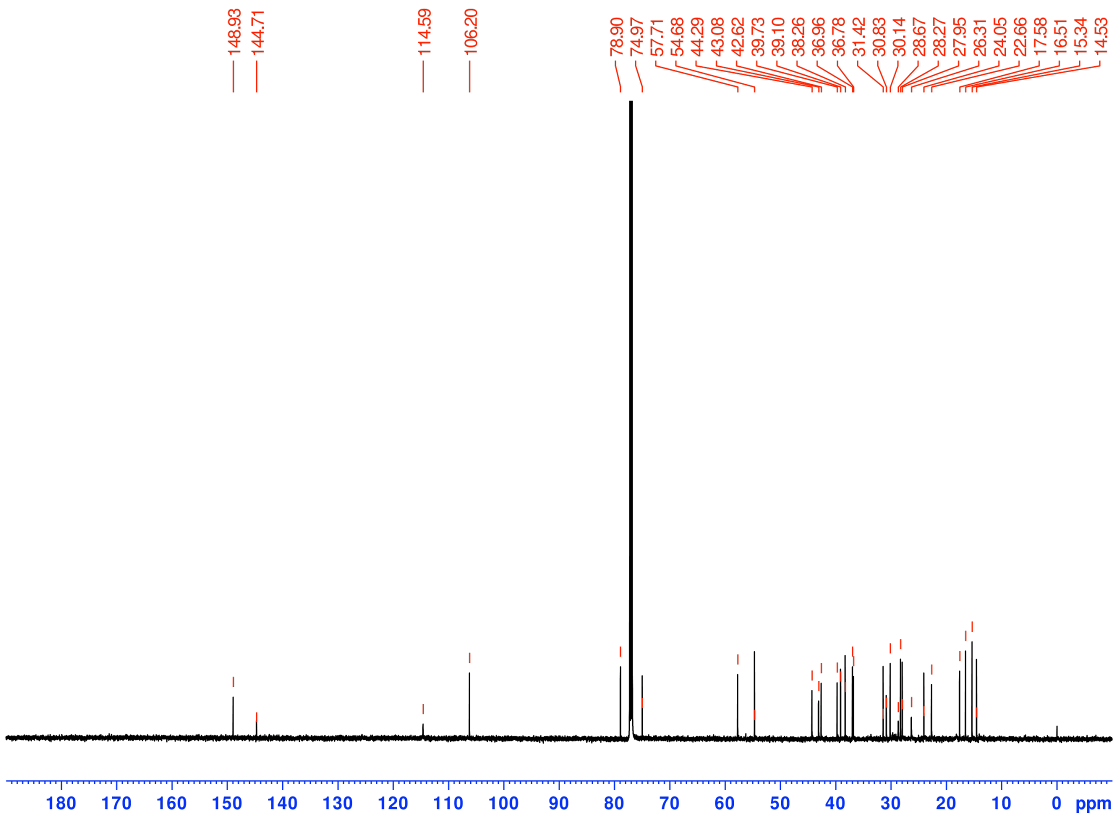

Supplementary Fig. 36. ^{13}C NMR spectrum of **6** in $CDCl_3$ at 150 MHz.

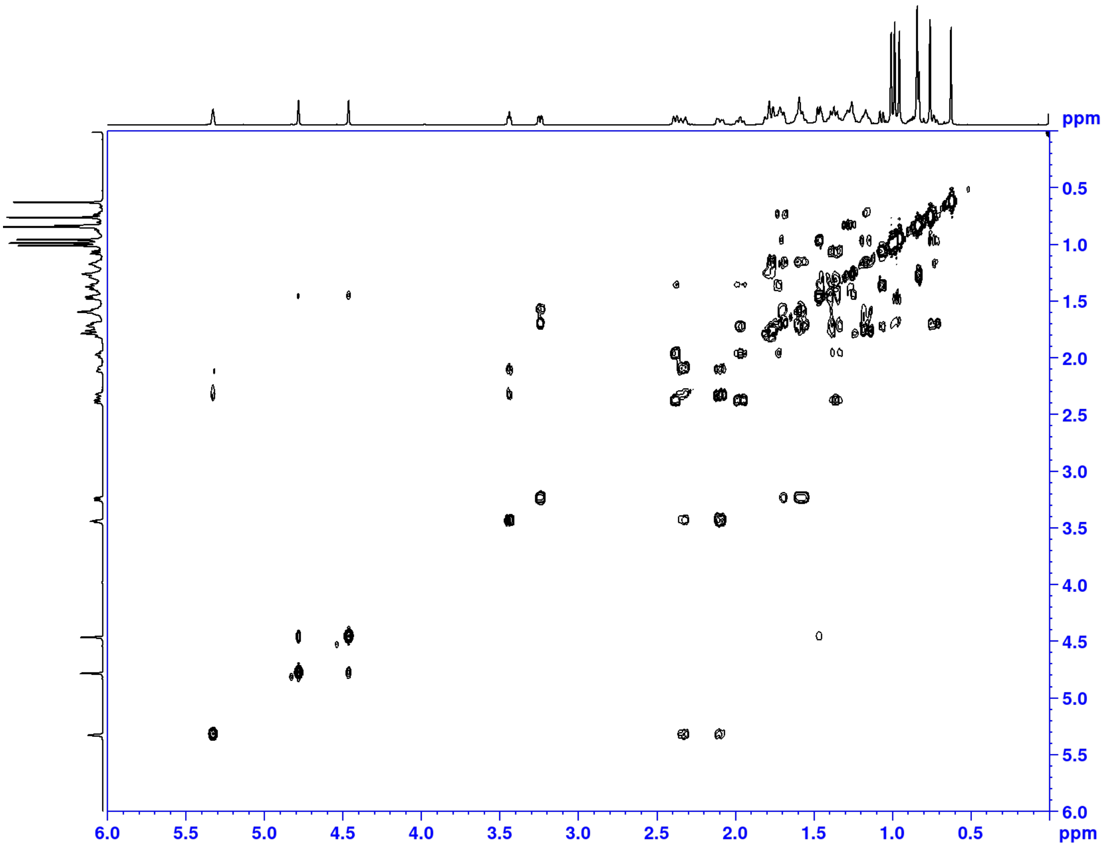

Supplementary Fig. 37. ^{1}H-^{1}H COSY spectrum of **6** in $CDCl_3$.

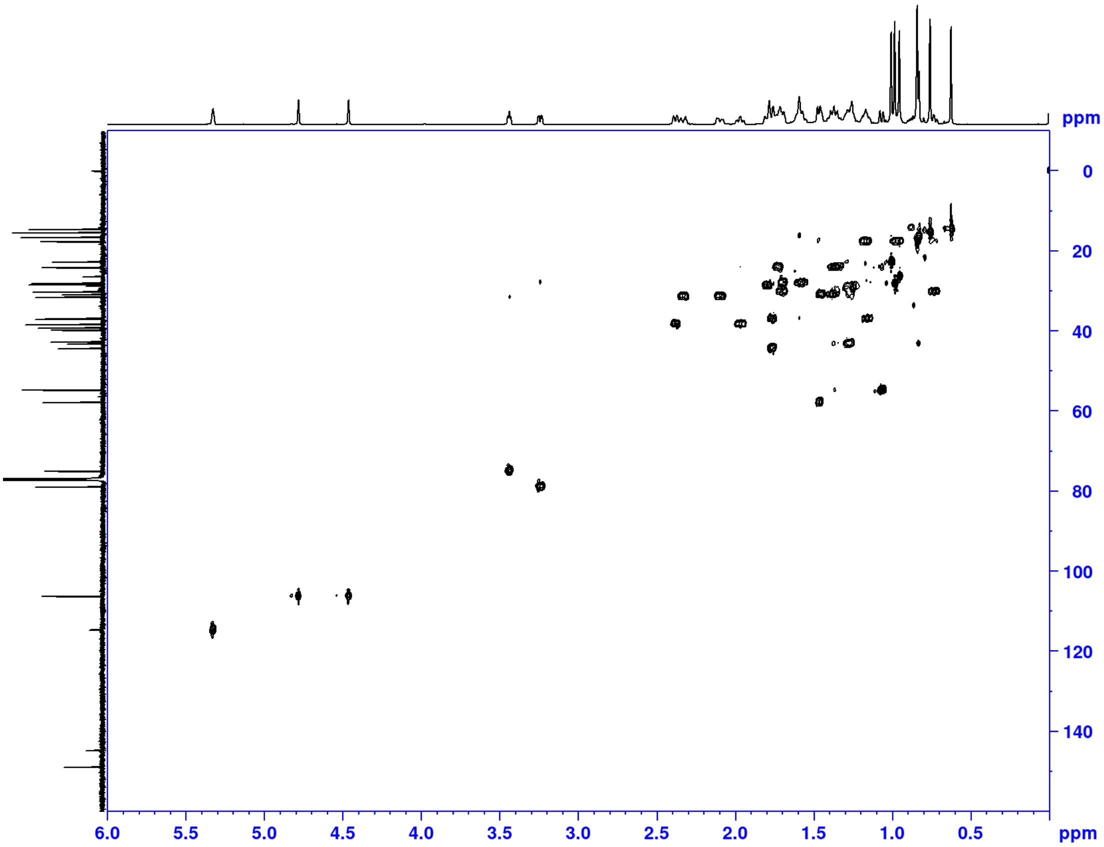

Supplementary Fig. 38. HSQC spectrum of **6** in $CDCl_3$.

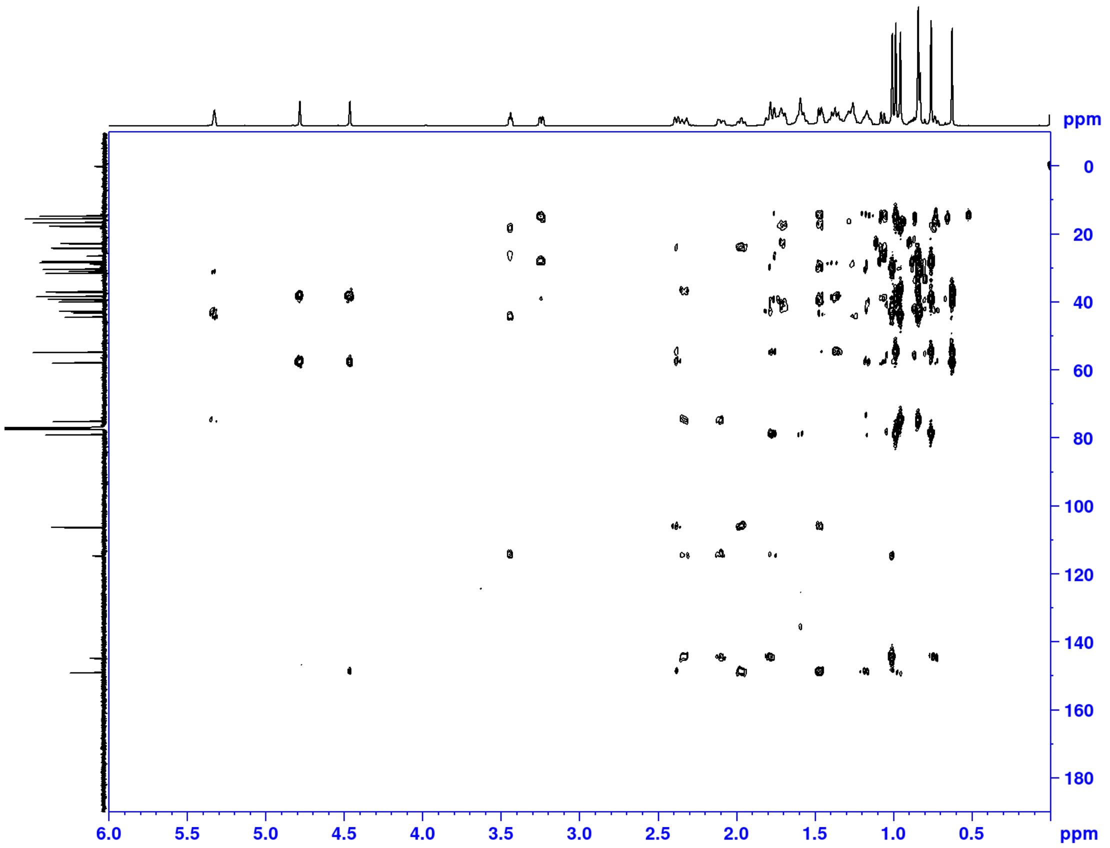

Supplementary Fig. 39. HMBC spectrum of **6** in $CDCl_3$.

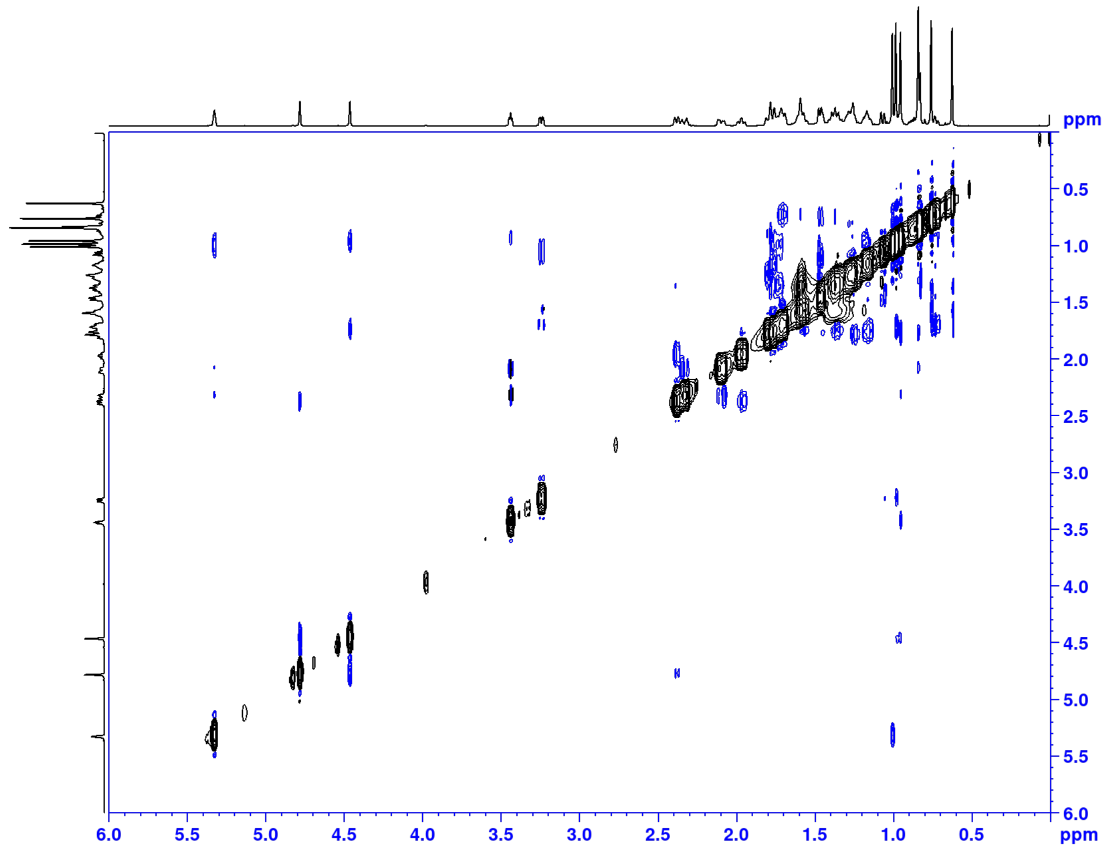

Supplementary Fig. 40. NOESY spectrum of **6** in $CDCl_3$.

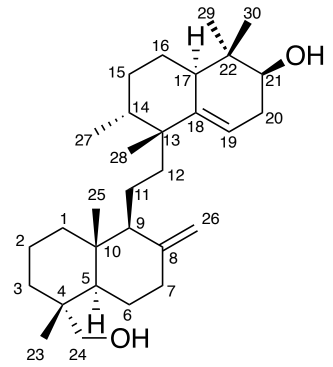

7

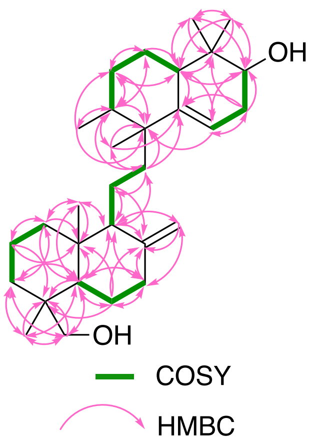

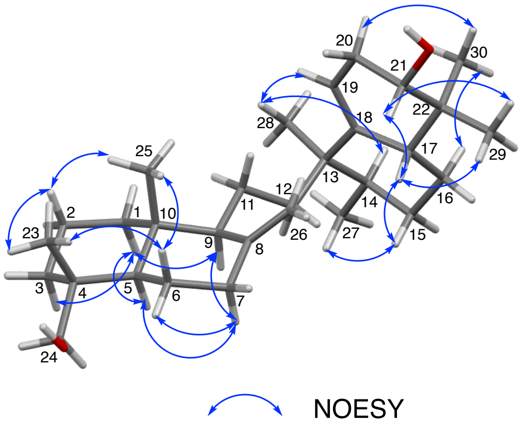

position	δC, type	δH, mult. (*J* in Hz)
1	38.5, CH_2	1.02 (α), td (13.0, 4.4)
		1.74 (β), m
2	18.7, CH_2	1.60 (α), m
		1.57 (β), m
3	35.5, CH_2	1.42 (α), m
		1.28 (β), m
4	38.0, C	
5	48.6, CH	1.41, m
6	24.3, CH_2	1.61 (α), m
		1.31 (β), qd (12.9, 4.3)
7	38.2, CH_2	2.00 (α), td (12.8, 5.1)
		2.35 (β), ddd (12.5, 4.0, 2.4)
8	149.3, C	
9	58.0, CH	1.56, m
10	39.9, C	
11	17.5, CH_2	1.20, m
		0.96, m
12	30.2, CH_2	1.70, td (13.0, 5.0)
		0.74, m
13	42.7, C	
14	43.1, CH	1.27, m
15	30.8, CH_2	1.38 (α), qd (13.0, 3.2)
		1.46 (β), m
16	28.7, CH_2	1.80 (α), m
		1.25 (β), m
17	44.3, CH	1.78, m
18	144.8, C	
19	114.5, CH	5.33, t (3.7)
20	31.4, CH_2	2.33 (α), m
		2.09 (β), m
21	75.0, CH	3.44, dd (7.2, 5.0)
22	36.8, C	
23	17.6, CH_3	0.74, s
24	72.1, CH_2	3.40, d (10.9)
		3.10, d (10.9)
25	15.0, CH_3	0.66, s
26	106.0, CH_2	4.77, brs
		4.45, brs
27	16.5, CH_3	0.83, d (7.0)
28	22.7, CH_3	1.01, s
29	26.3, CH_3	0.95, s
30	18.2, CH_3	0.84, s

^{1}H NMR: 600 MHz, ^{13}C NMR: 150 MHz (in $CDCl_3$)

Supplementary Fig. 41. NMR data of fumionoceroid C (**7**).

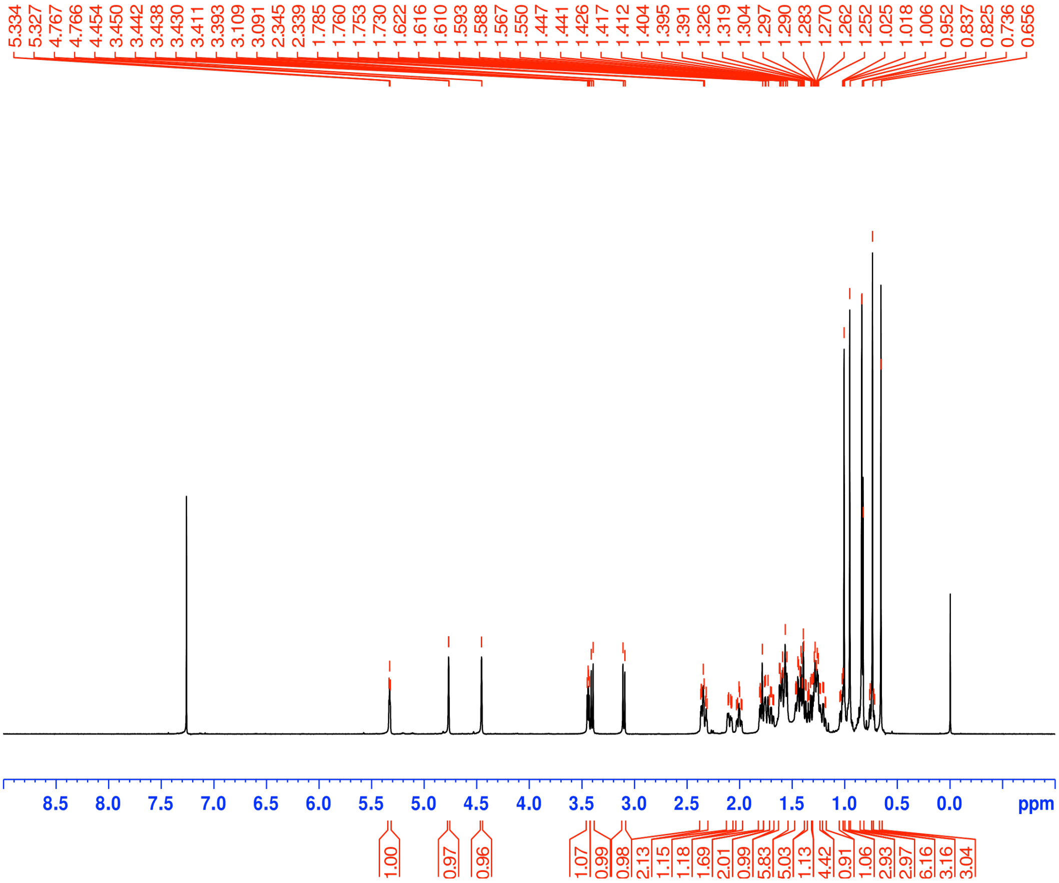

Supplementary Fig. 42. ^{1}H NMR spectrum of **7** in $CDCl_3$ at 600 MHz.

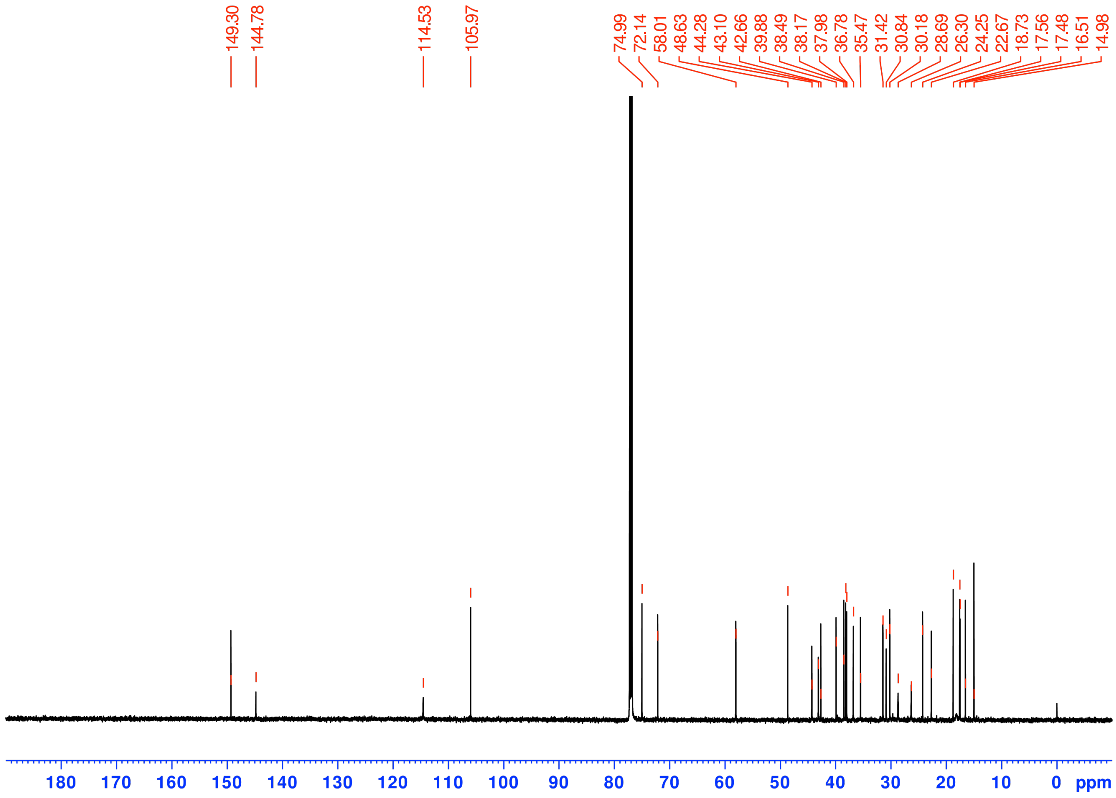

Supplementary Fig. 43. ^{13}C NMR spectrum of **7** in $CDCl_3$ at 150 MHz.

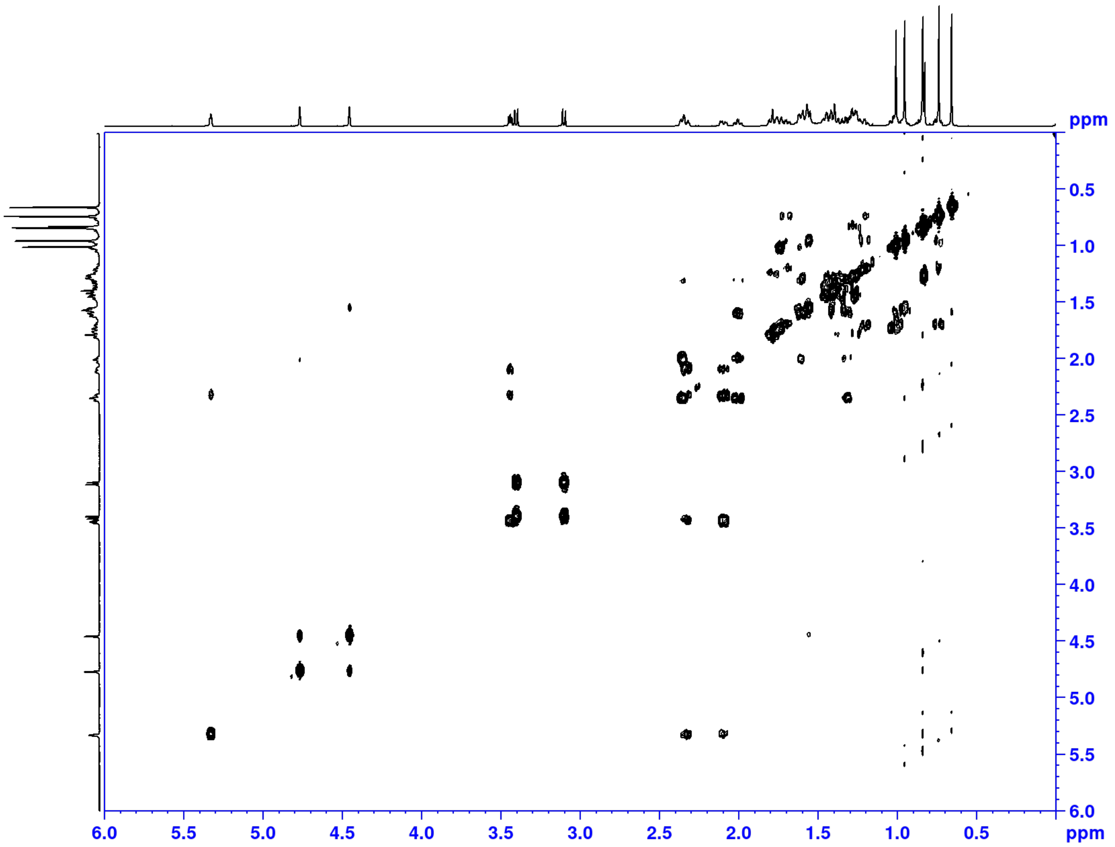

Supplementary Fig. 44. ^{1}H-^{1}H COSY spectrum of **7** in $CDCl_3$.

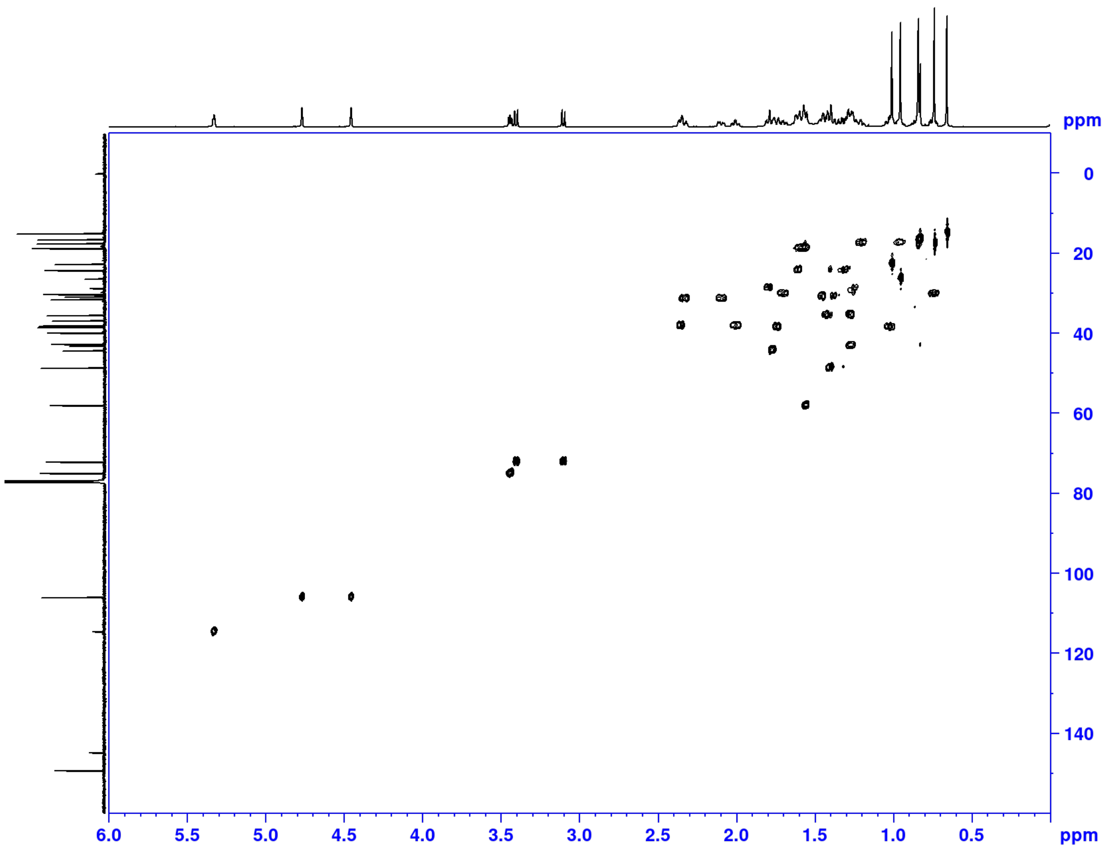

Supplementary Fig. 45. HSQC spectrum of **7** in $CDCl_3$.

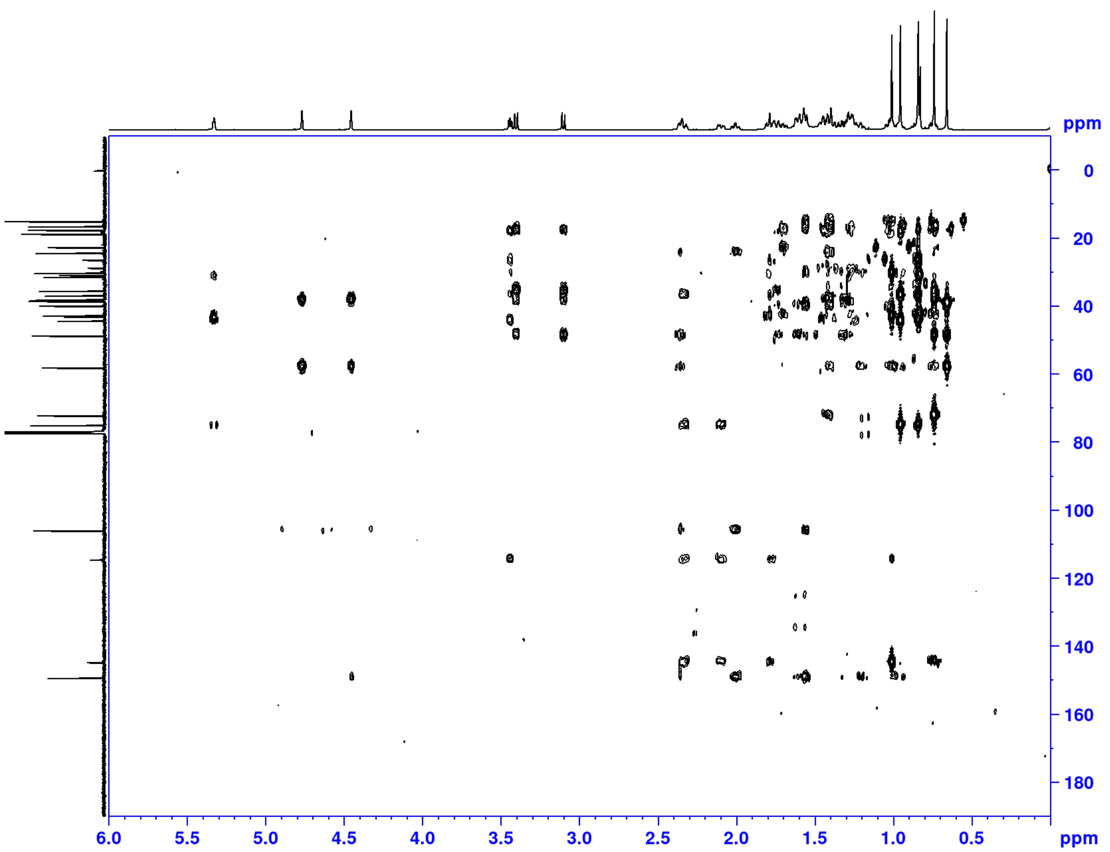

Supplementary Fig. 46. HMBC spectrum of **7** in $CDCl_3$.

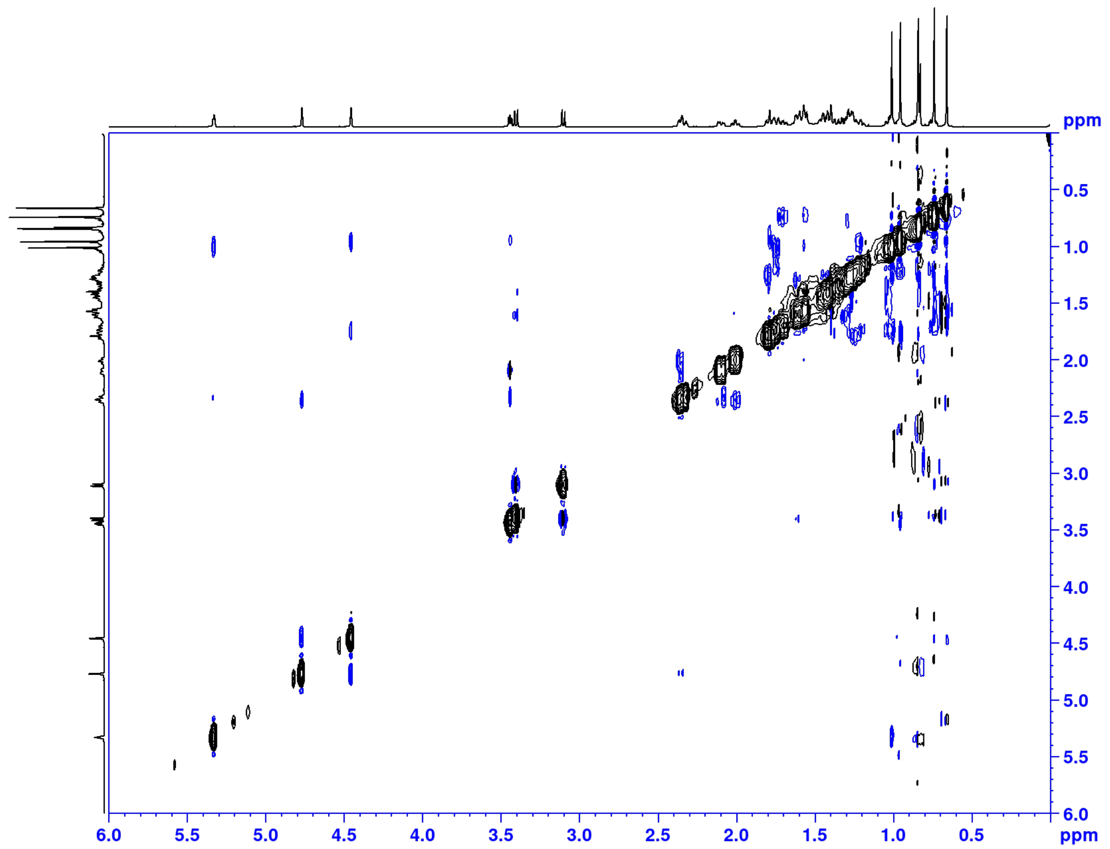

Supplementary Fig. 47. NOESY spectrum of **7** in $CDCl_3$.

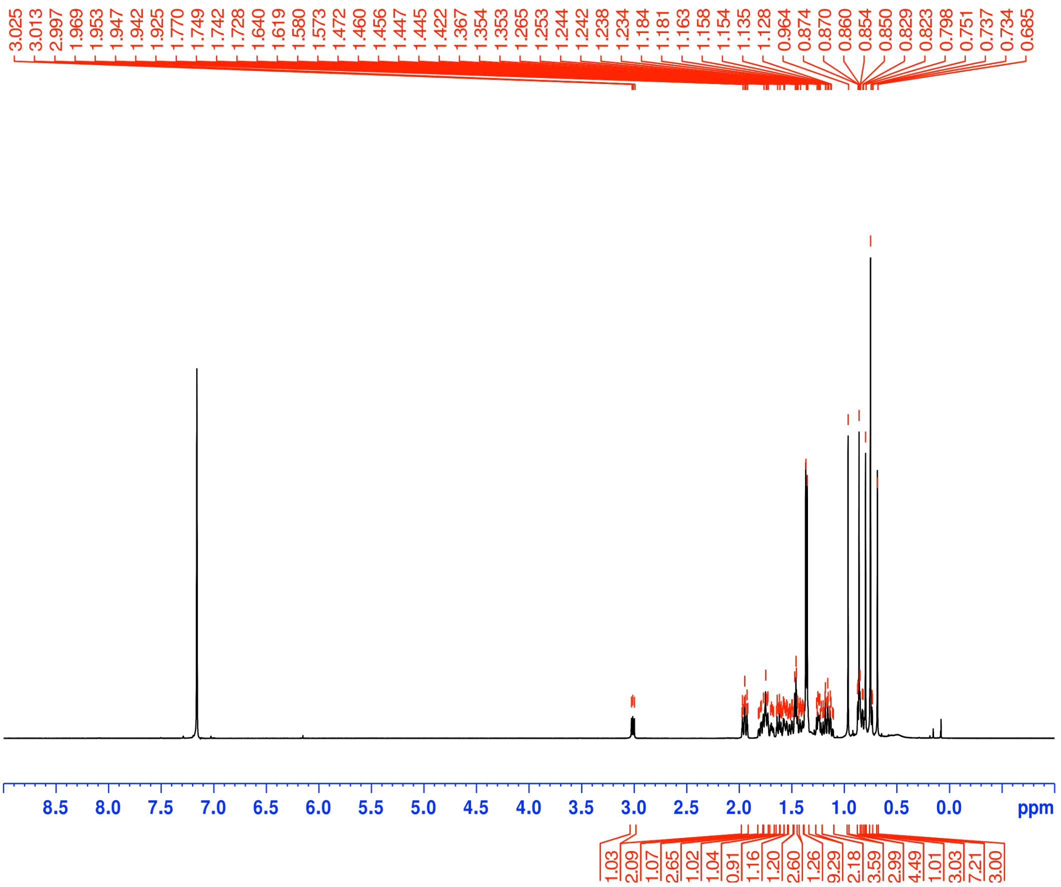

Supplementary Fig. 48. ^{1}H NMR spectrum of **8** in C_6D_6 at 600 MHz.

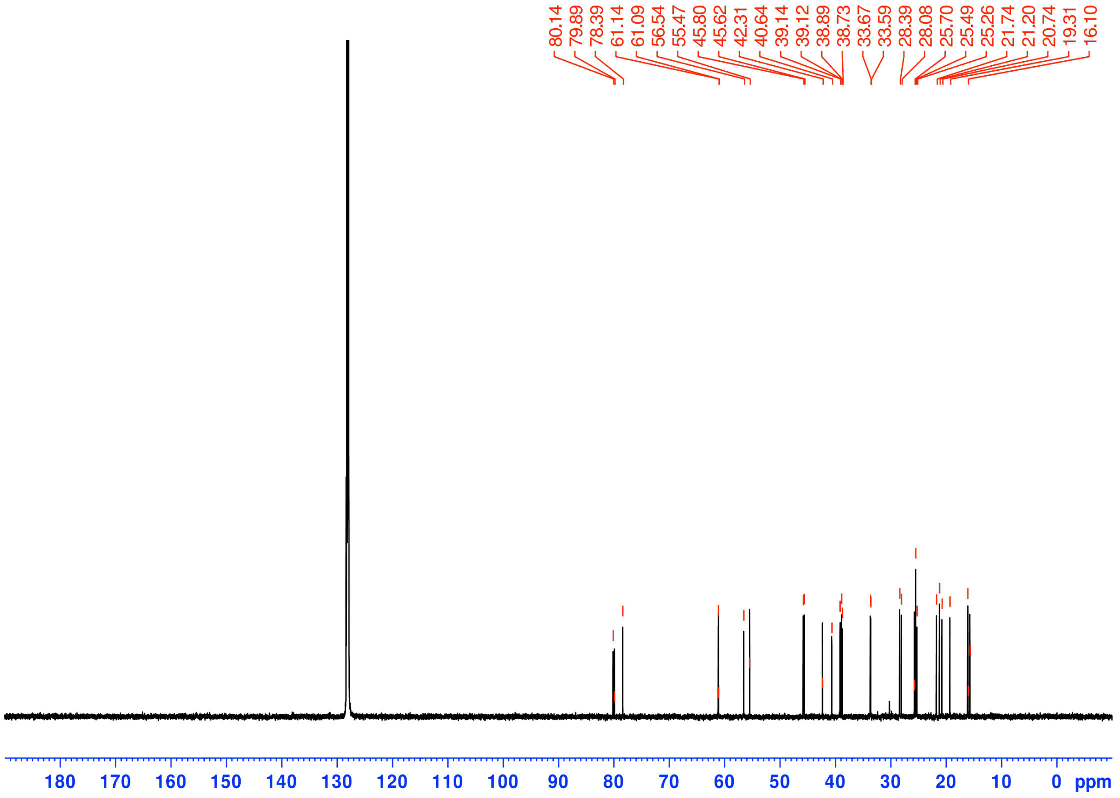

Supplementary Fig. 49. ^{13}C NMR spectrum of **8** in C_6D_6 at 150 MHz.

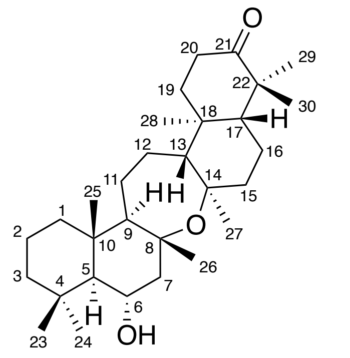

9

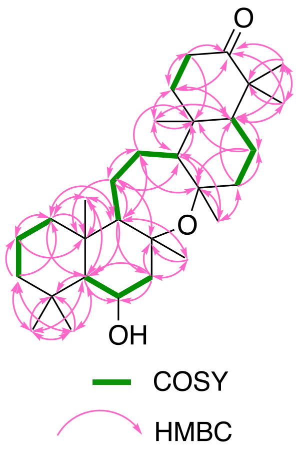

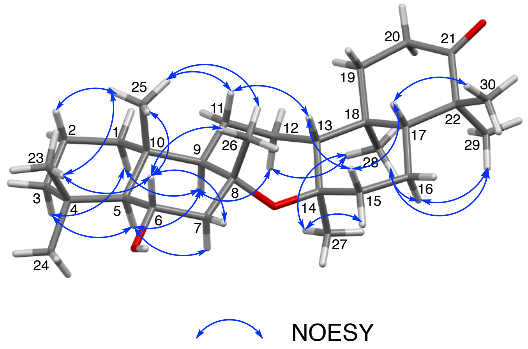

position	δ_C, type	δ_H, mult. (J in Hz)
1	40.4, CH_2	0.93 (α), td (12.5, 3.7)
		1.74 (β), m
2	18.6, CH_2	1.43 (α), m
		1.57 (β), m
3	43.3, CH_2	1.19 (α), m
		1.34 (β), m
4	33.8, C	
5	61.0, CH	0.97, brd (10.2)
6	69.4, CH	3.77, brt (10.6)
7	56.2, CH_2	1.66 (α), t (11.7)
		2.09 (β), dd (11.8, 3.0)
8	79.4, C	
9	60.0, CH	1.43, m
10	39.4, C	
11	24.8, CH_2	1.82 (α), m
		1.22 (β), m
12	25.5, CH_2	1.29 (α), m
		1.75 (β), m
13	59.6, CH	1.48, brd (11.0)
14	79.7, C	
15	44.4, CH_2	1.80 (α), m
		1.60 (β), m
16	21.7, CH_2	1.37 (α), m
		1.53 (β), m
17	54.4, CH	1.51, dd (12.6, 1.9)
18	38.4, C	
19	39.2, CH_2	1.99 (α), dt (12.8, 6.0)
		1.53 (β), m
20	33.9, CH_2	2.47, m
21	217.8, C	
22	47.2, C	
23	22.2, CH_3	0.98, s
24	36.4, CH_3	1.15, s
25	17.3, CH_3	0.78, s
26	26.7, CH_3	1.32, s
27	24.7, CH_3	1.29, s
28	15.7, CH_3	0.83, s
29	20.9, CH_3	1.00, s
30	27.1, CH_3	1.08, s

^{1}H NMR: 600 MHz, ^{13}C NMR: 150 MHz (in $CDCl_3$)

Supplementary Fig. 50. NMR data of alliaonoceroid B (**9**).

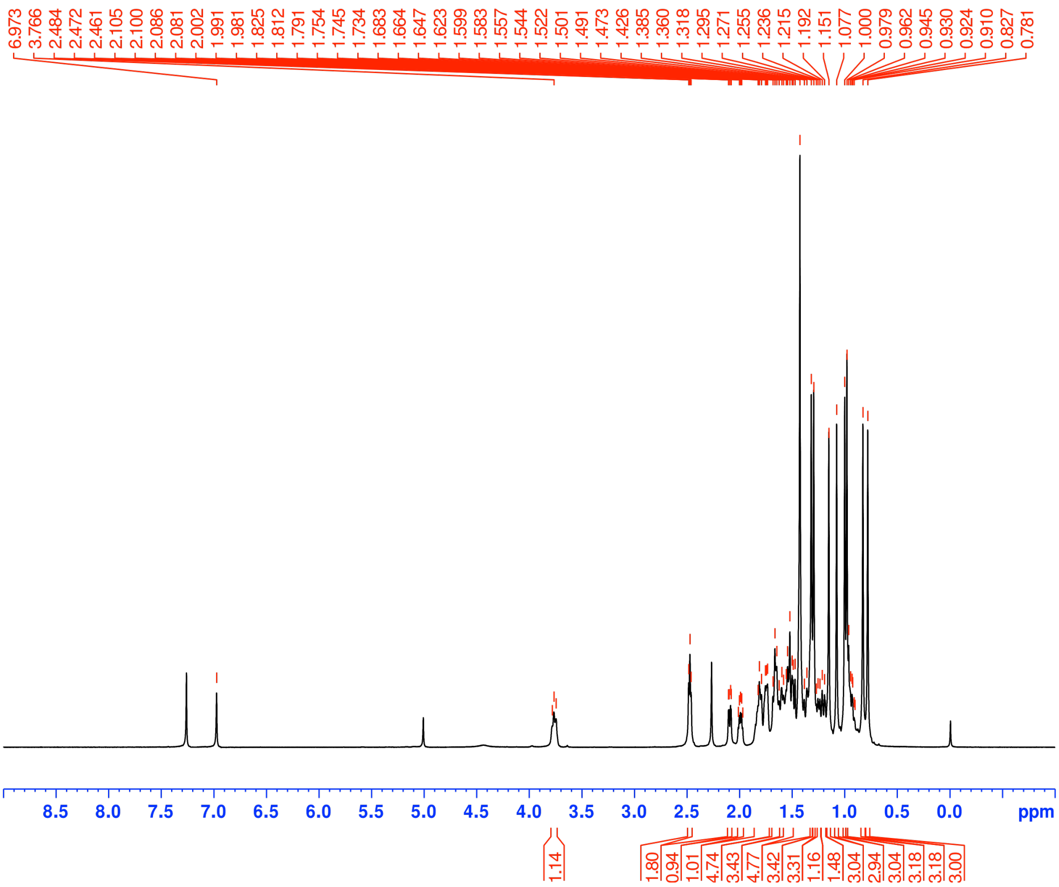

Supplementary Fig. 51. ^{1}H NMR spectrum of **9** in $CDCl_3$ at 600 MHz.

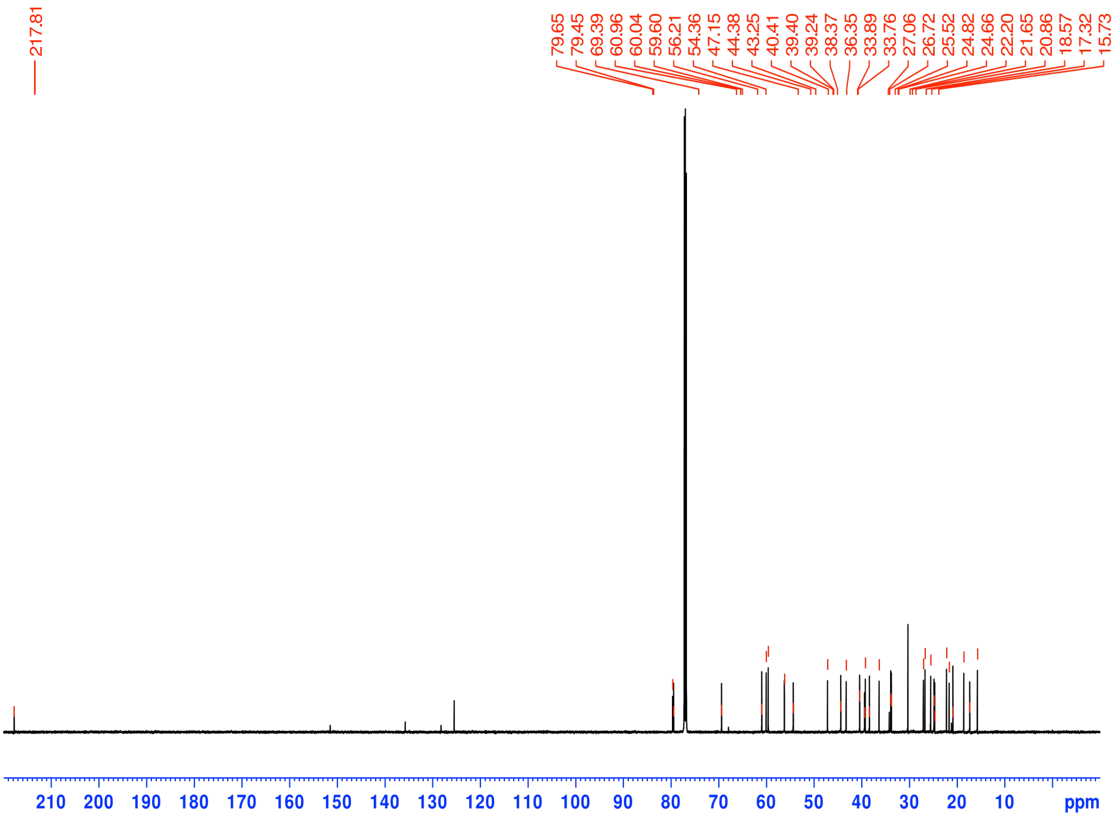

Supplementary Fig. 52. ^{13}C NMR spectrum of **9** in $CDCl_3$ at 150 MHz.

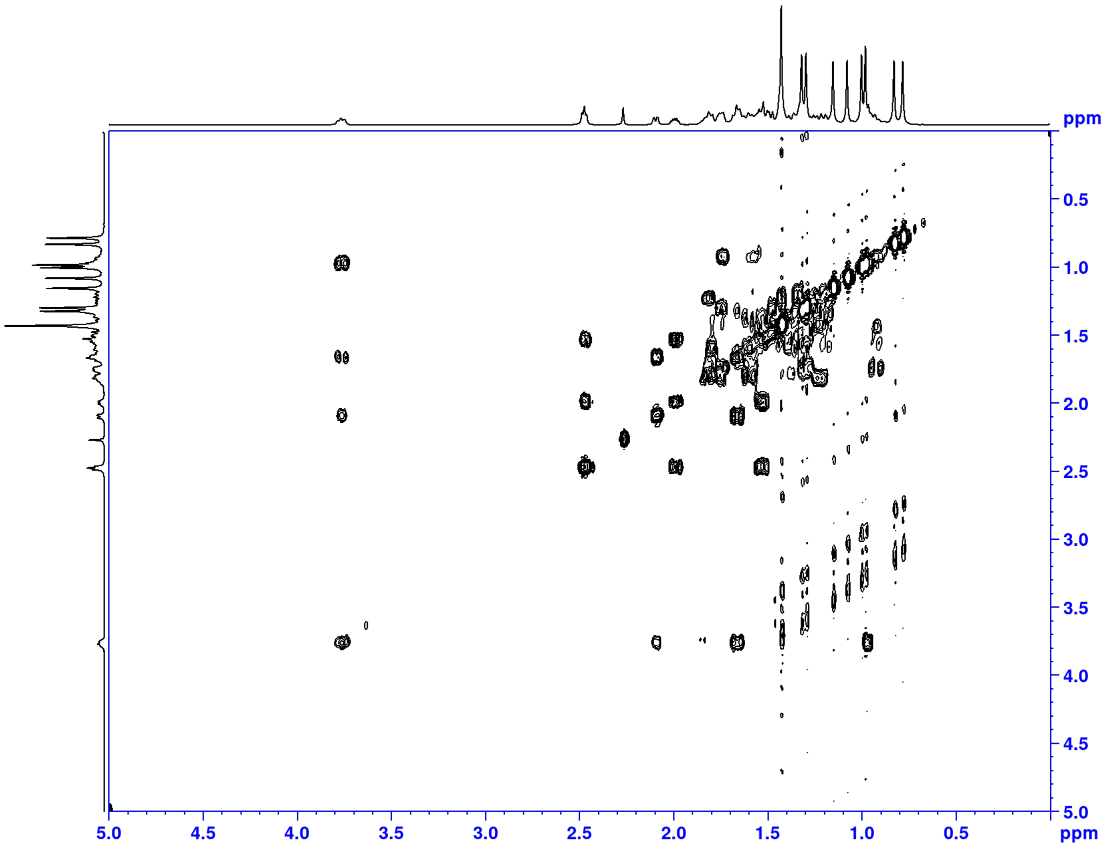

Supplementary Fig. 53. ^{1}H-^{1}H COSY spectrum of **9** in $CDCl_3$.

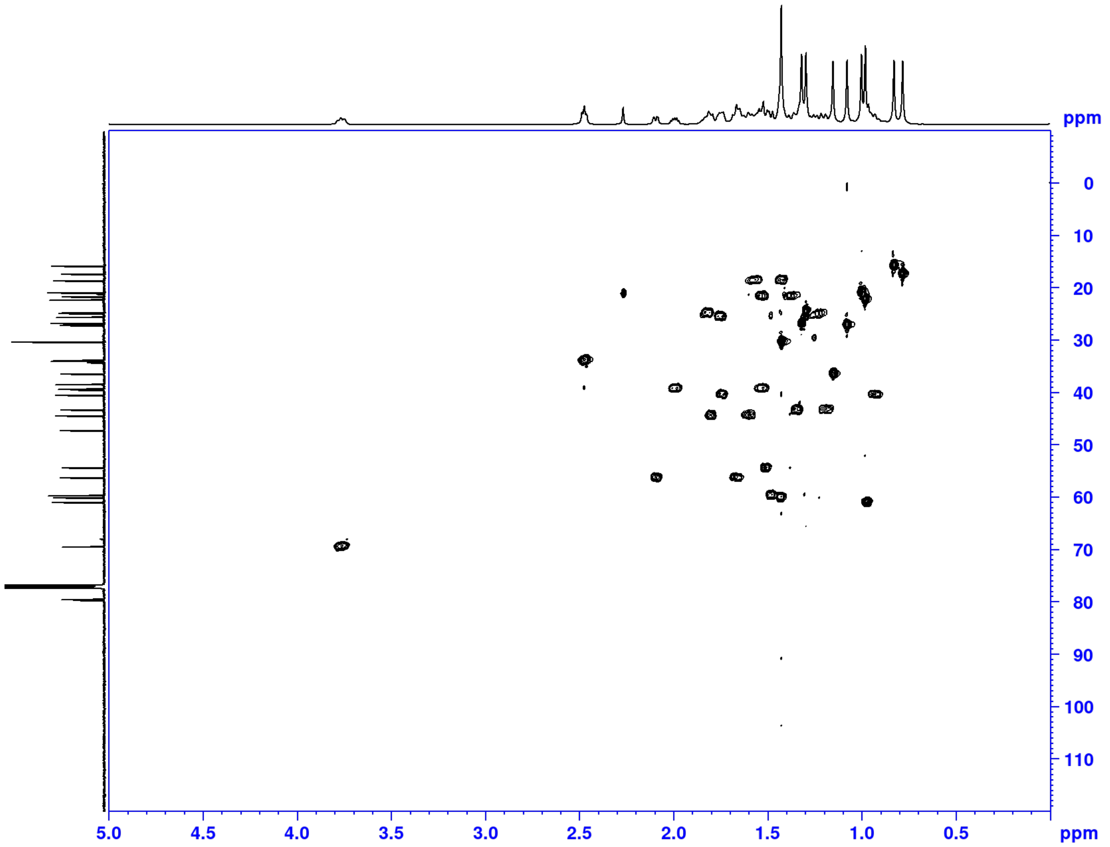

Supplementary Fig. 54. HSQC spectrum of **9** in $CDCl_3$.

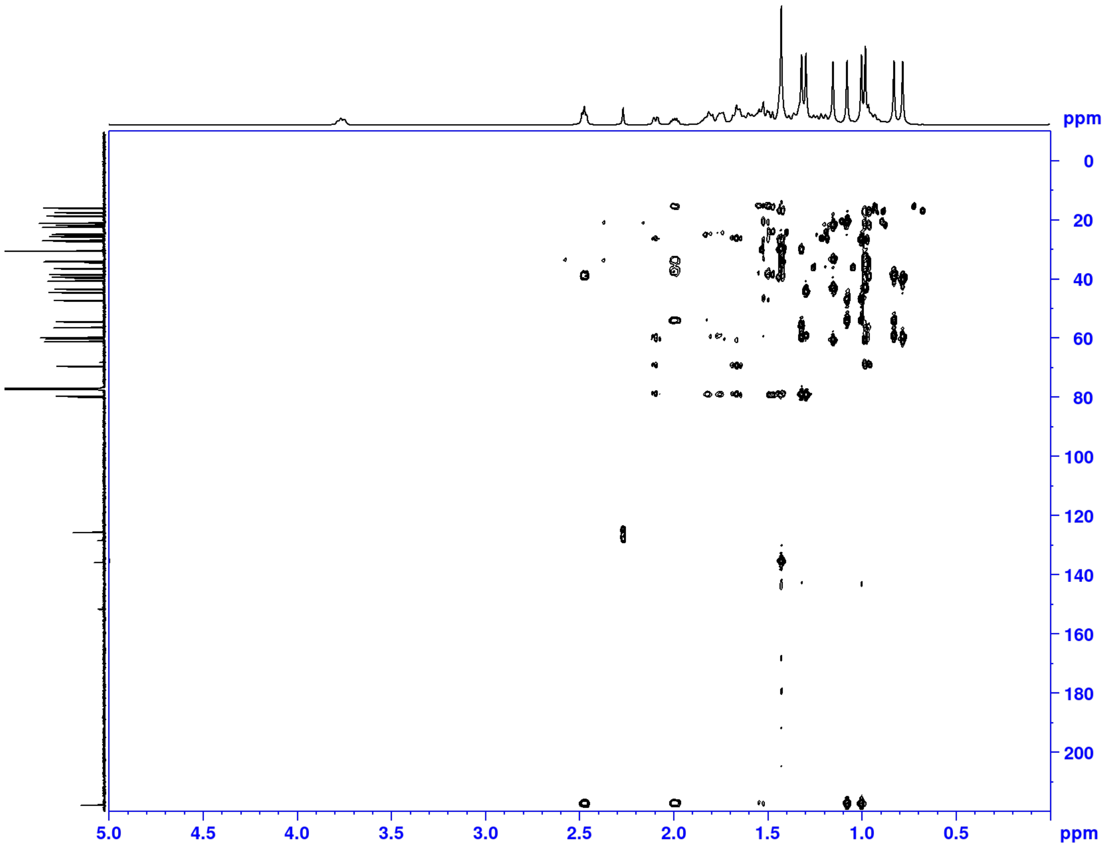

Supplementary Fig. 55. HMBC spectrum of **9** in $CDCl_3$.

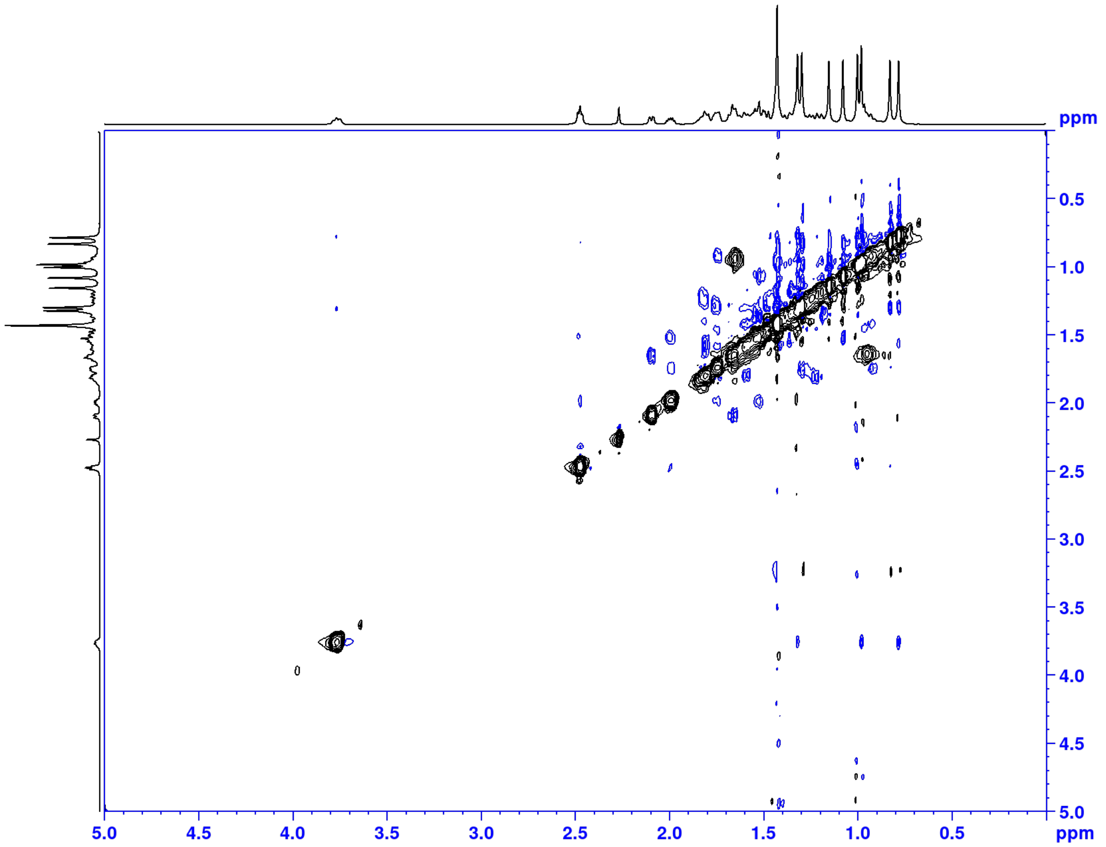

Supplementary Fig. 56. NOESY spectrum of **9** in $CDCl_3$.

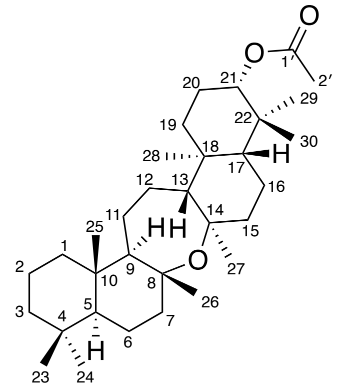

10

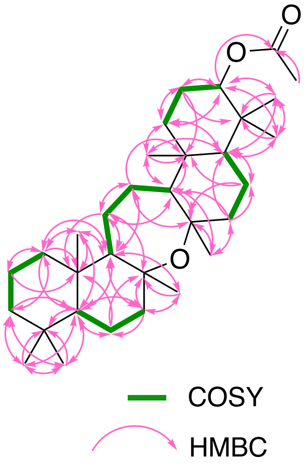

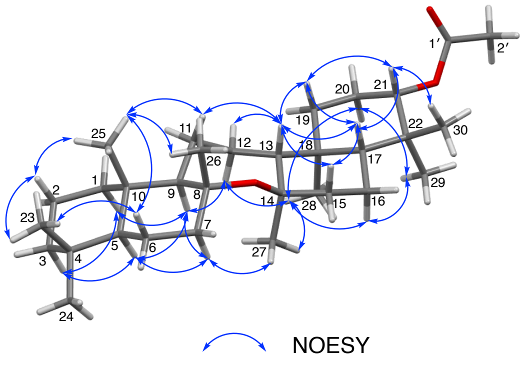

position	δ_C, type	δ_H, mult. (J in Hz)
1	40.3, CH_2	0.87 (α), m
		1.77 (β), m
2	18.9, CH_2	1.42 (α), dq (14.2, 3.5)
		1.59 (β), m
3	42.0, CH_2	1.11 (α), td (13.4, 4.0)
		1.34 (β), dt (13.7, 3.5)
4	33.4, C	
5	56.2, CH	0.87, m
6	20.7, CH_2	1.58 (α), m
		1.22 (β), m
7	45.2, CH_2	1.56 (α), m
		1.75 (β), m
8	80.1, C	
9	60.7, CH	1.38, dd (8.6, 3.5)
10	38.8, C	
11	24.8[a], CH_2	1.75 (α), m
		1.22 (β), m
12	25.0[a], CH_2	1.22 (α), m
		1.75 (β), m
13	60.4, CH	1.38, dd (8.6, 3.5)
14	79.6, C	
15	45.1, CH_2	1.75 (α), m
		1.56 (β), m
16	20.3, CH_2	1.29 (α), m
		1.58 (β), m
17	55.1, CH	0.95, dd (12.6, 1.9)
18	38.4, C	
19	38.2, CH_2	1.80 (α), dt (13.2, 3.5)
		1.11 (β), td (13.4, 4.0)
20	23.8, CH_2	1.62 (α), td (13.1, 3.9)
		1.67 (β), m
21	80.8, CH	4.46, dd (11.8, 4.8)
22	37.8, C	
23	21.5, CH_3	0.77, s
24	33.4, CH_3	0.84, s
25	15.8, CH_3	0.73, s
26	25.2, CH_3	1.25, s
27	25.2, CH_3	1.26, s
28	15.9, CH_3	0.77, s
29	16.4, CH_3	0.81, s
30	28.1, CH_3	0.84, s
1′	171.0, C	
2′	21.3, CH_3	2.04, s

^1H NMR: 600 MHz, ^{13}C NMR: 150 MHz (in $CDCl_3$)

[a]These signals are interchangeable.

Supplementary Fig. 57. NMR data of alliaonoceroid C (**10**).

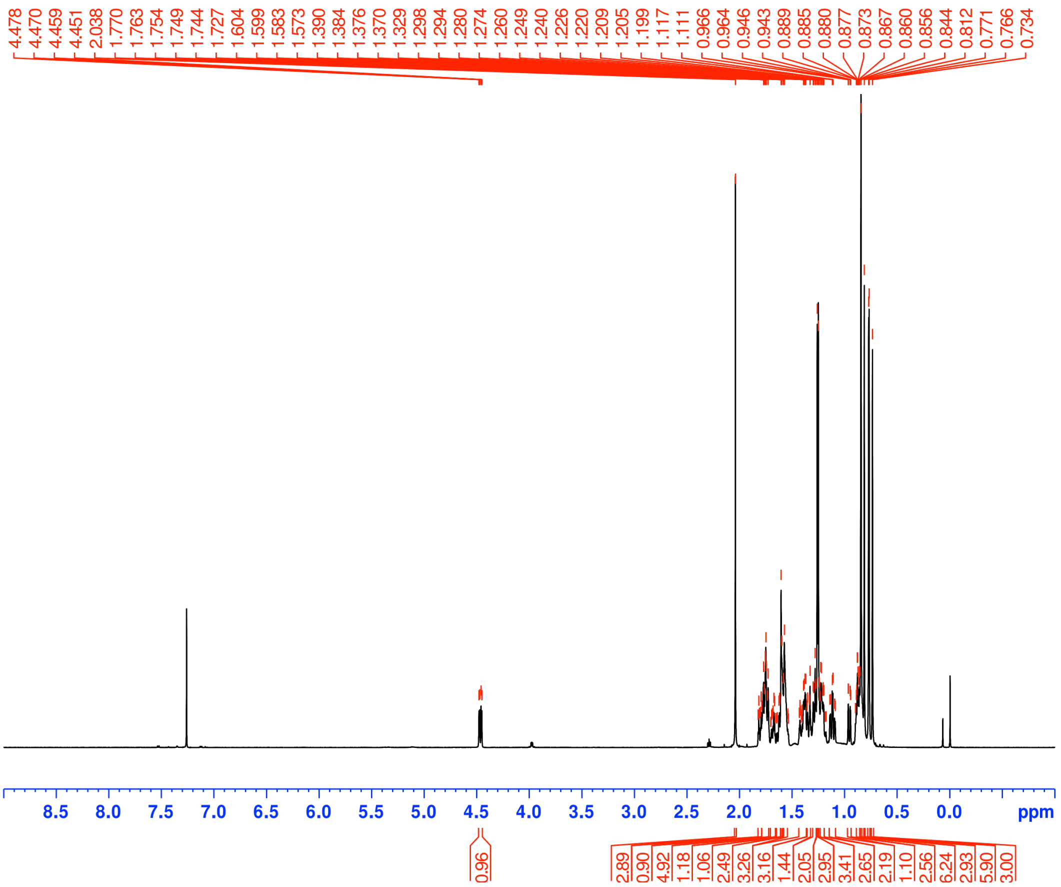

Supplementary Fig. 58. ^{1}H NMR spectrum of **10** in $CDCl_3$ at 600 MHz.

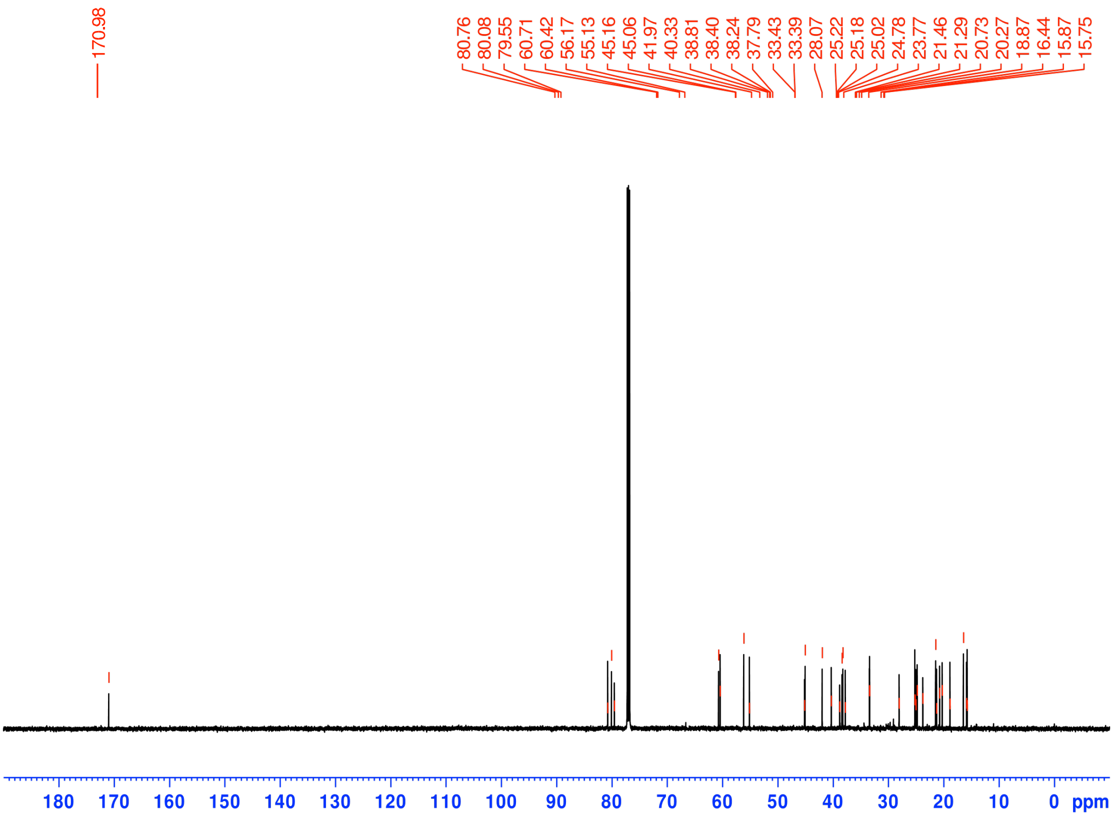

Supplementary Fig. 59. ^{13}C NMR spectrum of **10** in $CDCl_3$ at 150 MHz.

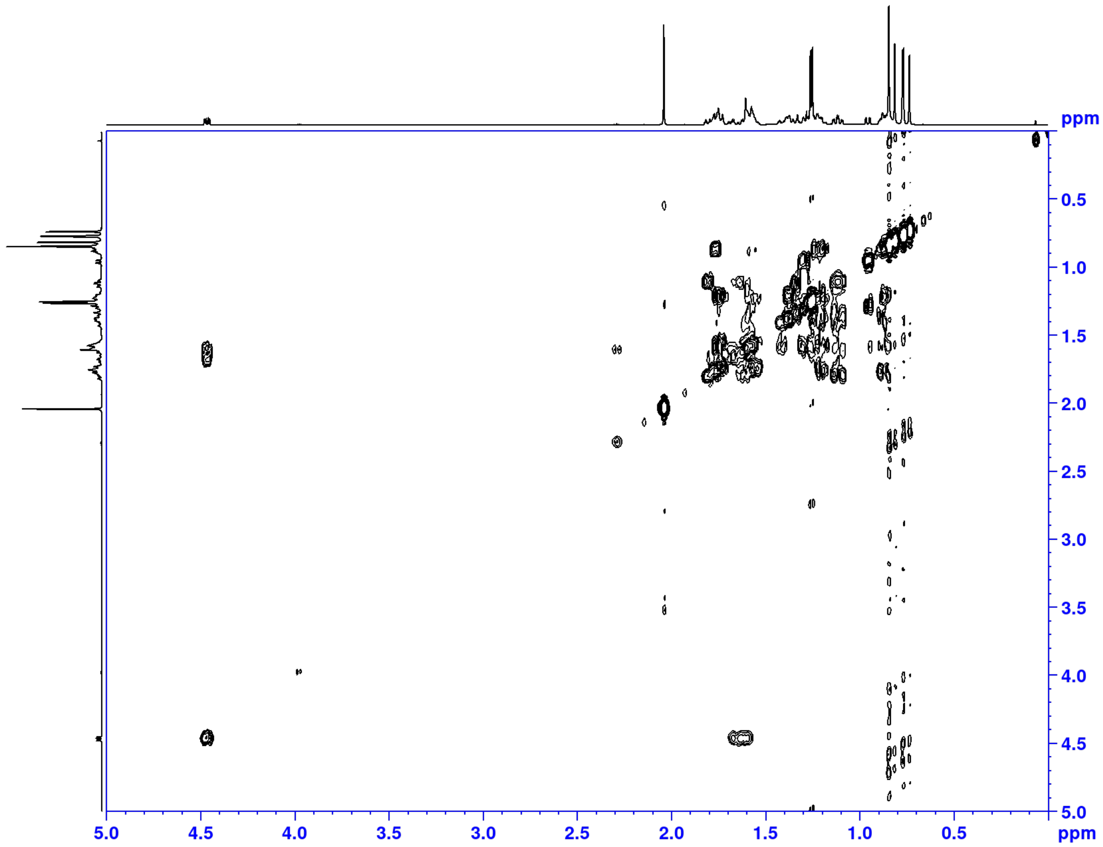

Supplementary Fig. 60. ^{1}H-^{1}H COSY spectrum of **10** in $CDCl_3$.

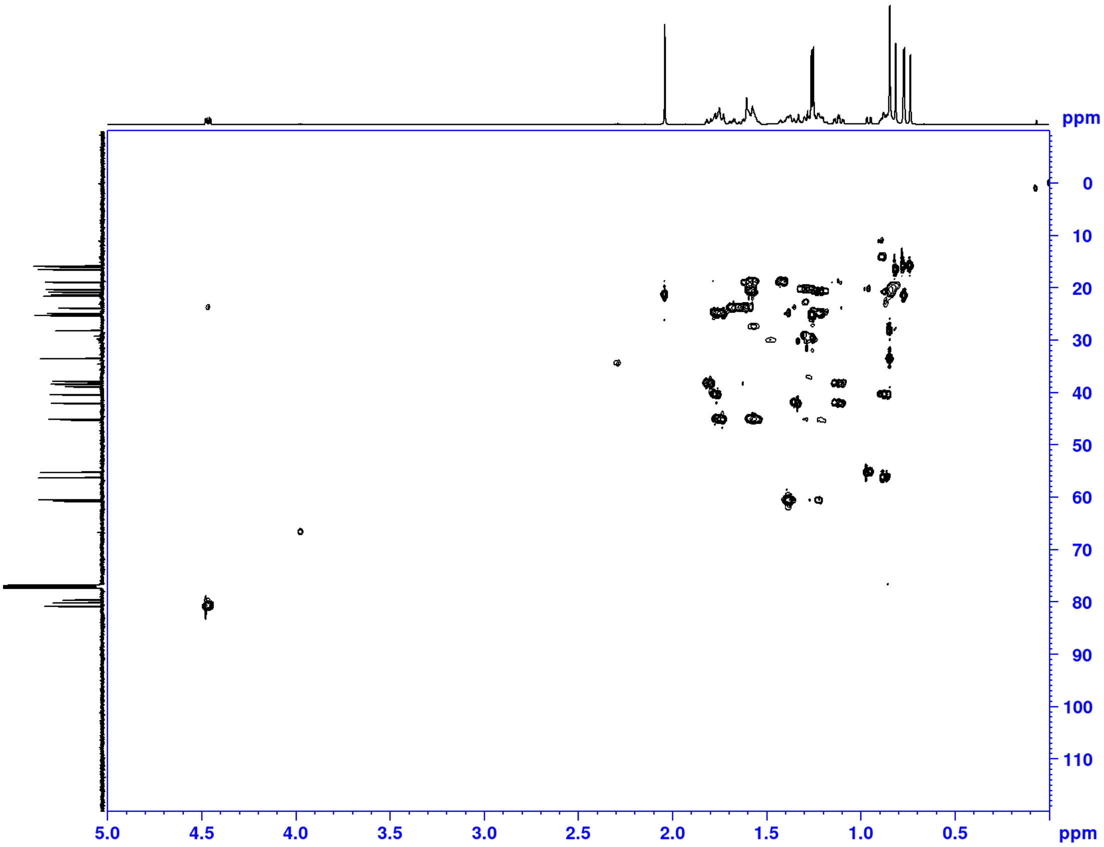

Supplementary Fig. 61. HSQC spectrum of **10** in $CDCl_3$.

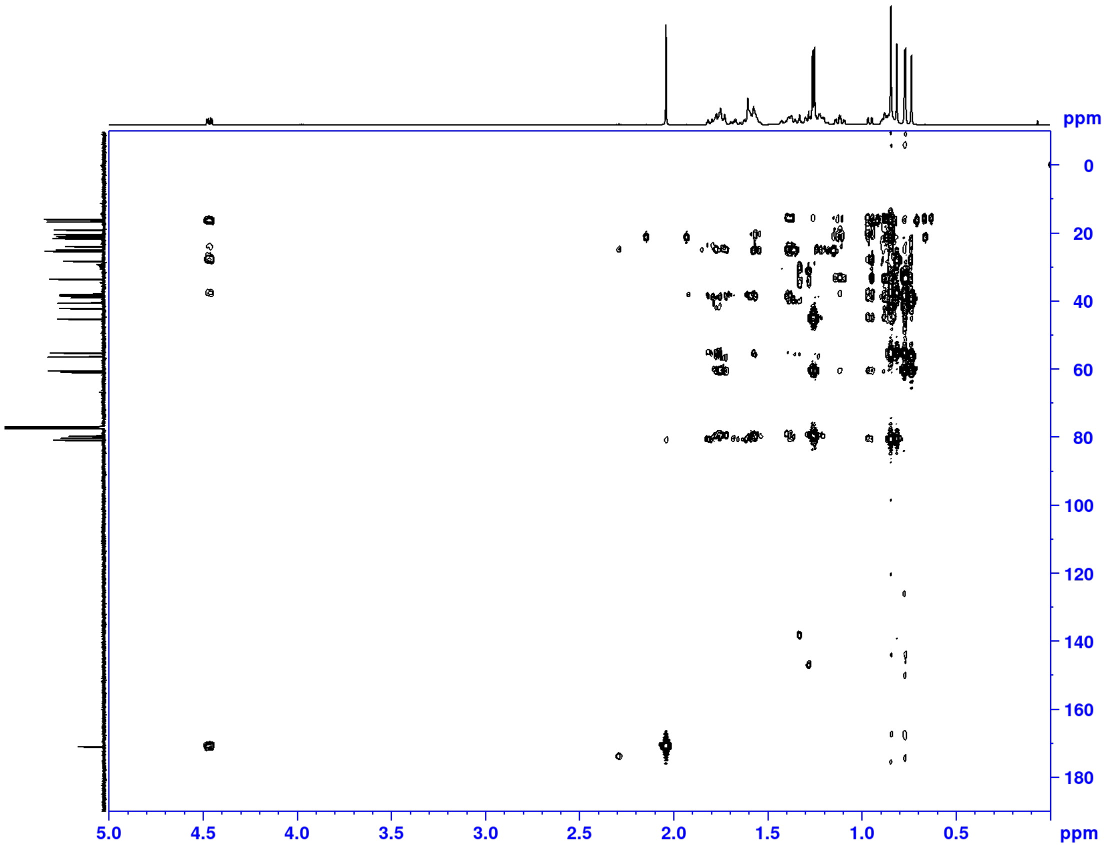

Supplementary Fig. 62. HMBC spectrum of **10** in $CDCl_3$.

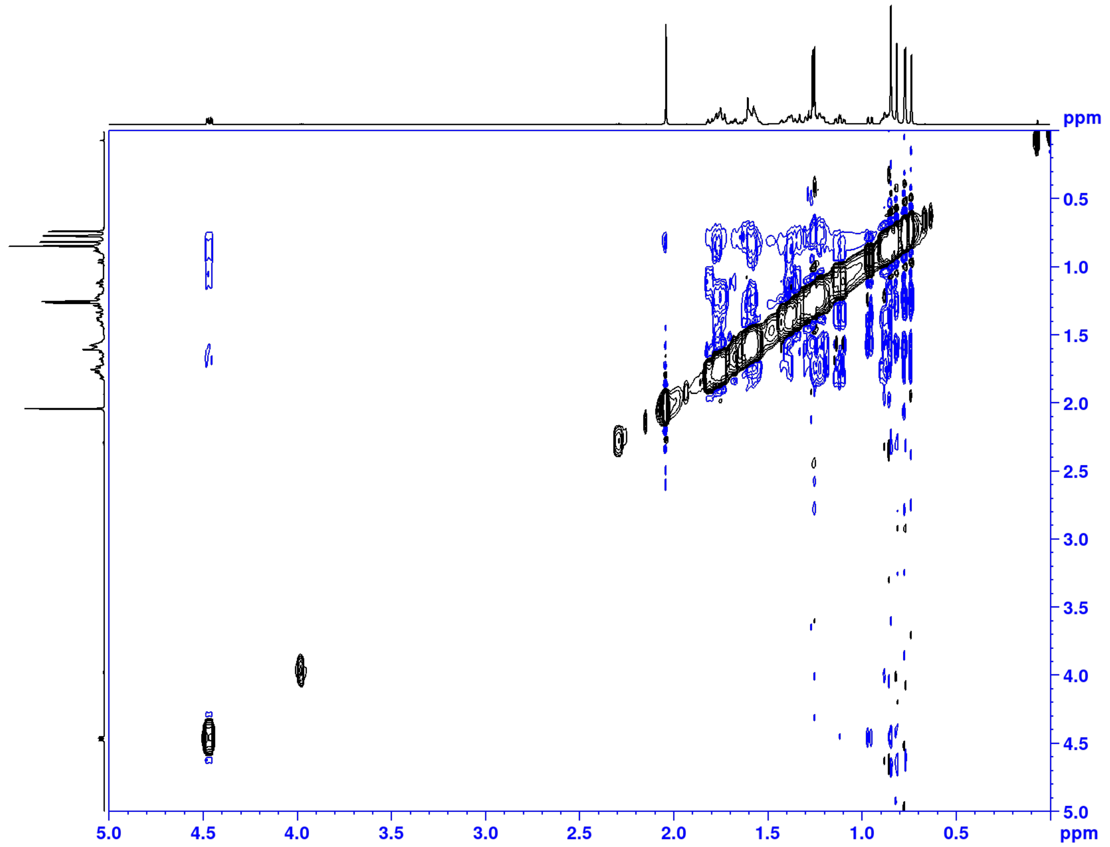

Supplementary Fig. 63. NOESY spectrum of **10** in $CDCl_3$.

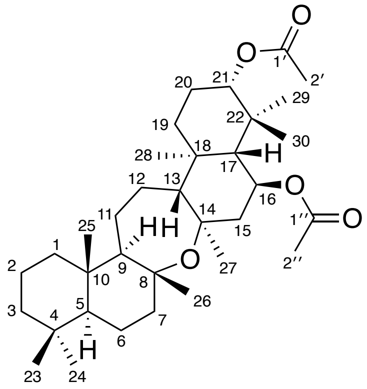

11

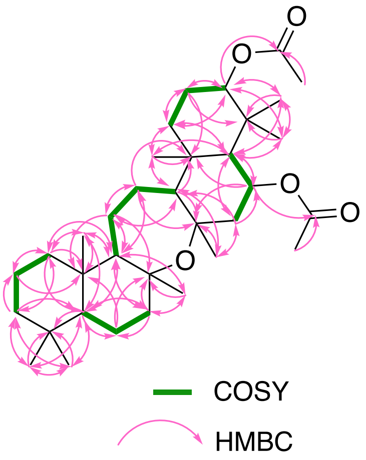

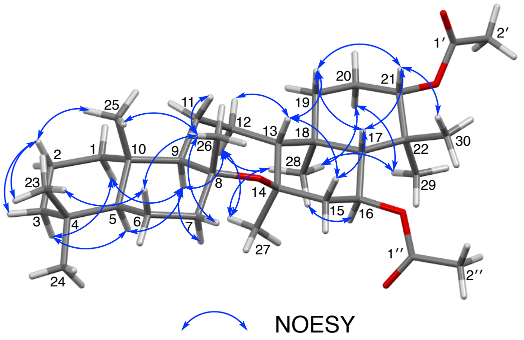

position	δ_C, type	δ_H, mult. (J in Hz)
1	40.5, CH_2	0.78 (α), m
		1.72 (β), m
2	19.3, CH_2	1.41 (α), m
		1.59 (β), qt (13.7, 3.3)
3	42.3, CH_2	1.13 (α), m
		1.36 (β), m
4	33.6, C	
5	56.5, CH	0.81, m
6	21.2, CH_2	1.54 (α), m
		1.14 (β), m
7	45.3, CH_2	1.71 (α), m
		1.90 (β), m
8	80.6, C	
9	60.3, CH	1.41, m
10	39.4, C	
11	24.9, CH_2	1.68 (α), m
		1.18 (β), m
12	25.1, CH_2	1.07 (α), m
		1.54 (β), m
13	60.5, CH	1.27, brd (10.6)
14	79.2, C	
15	52.7, CH_2	2.32 (α), dd (11.9, 3.6)
		1.90 (β), m
16	70.2, CH	5.32, td (11.4, 3.4)
17	58.2, CH	1.25, d (11.4)
18	39.2, C	
19	38.1, CH_2	1.46 (α), m
		0.76 (β), m
20	23.7, CH_2	1.53 (α), m
		1.73 (β), m
21	80.0, CH	4.65, dd (11.9, 4.5)
22	38.1, C	
23	21.7, CH_3	0.80, s
24	33.6, CH_3	0.86, s
25	16.1, CH_3	0.75, s
26	25.8, CH_3	1.33, s
27	26.4, CH_3	1.46, s
28	16.9, CH_3	0.69, s
29	17.2, CH_3	1.03, s
30	30.5, CH_3	1.19, s
1′	170.1, C	
2′	20.9, CH_3	1.77, s
1′′	169.3, C	
2′′	21.5, CH_3	1.70, s

^1H NMR: 600 MHz, ^{13}C NMR: 150 MHz (in C_6D_6)

Supplementary Fig. 64. NMR data of alliaonoceroid D (**11**).

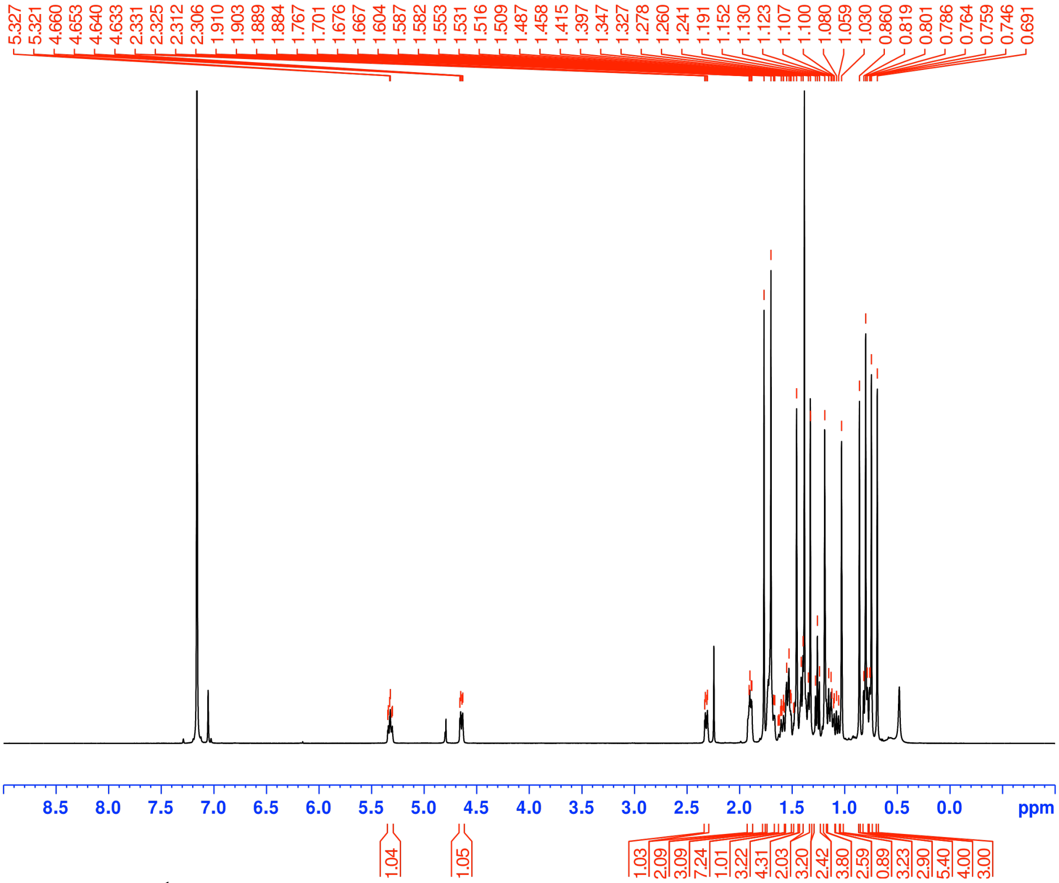

Supplementary Fig. 65. ^{1}H NMR spectrum of **11** in C_6D_6 at 600 MHz.

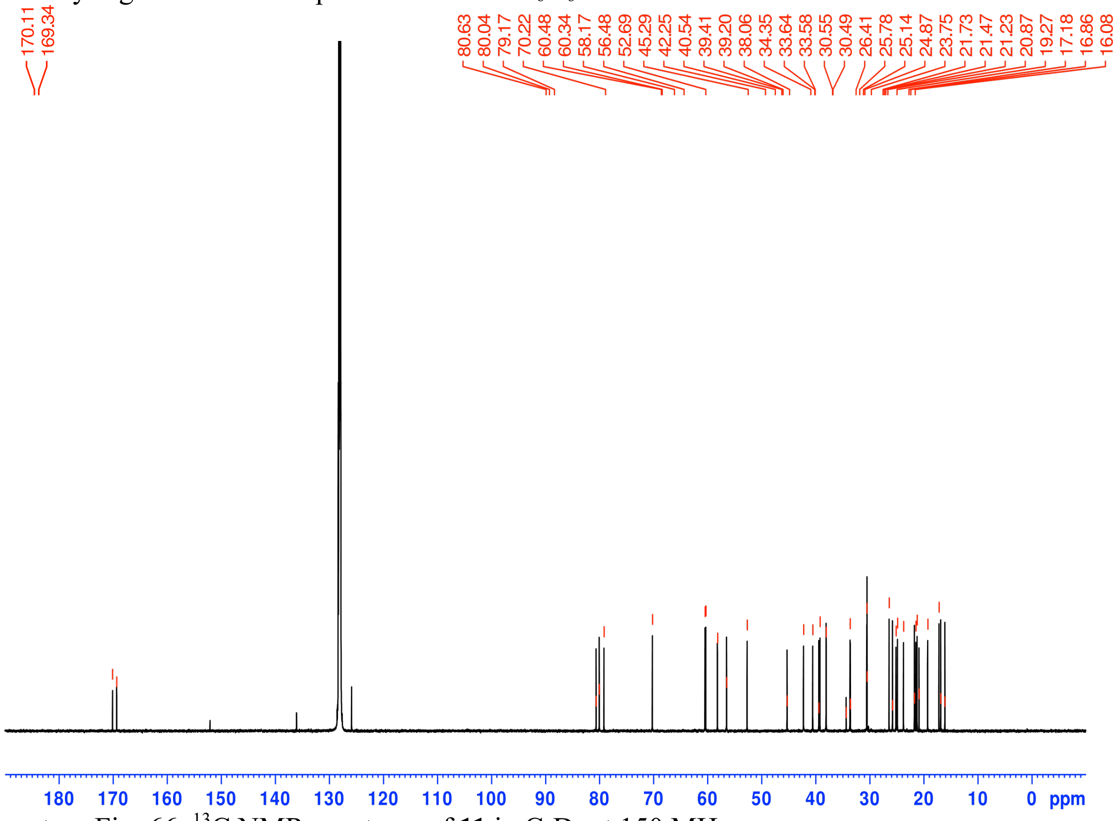

Supplementary Fig. 66. ^{13}C NMR spectrum of **11** in C_6D_6 at 150 MHz.

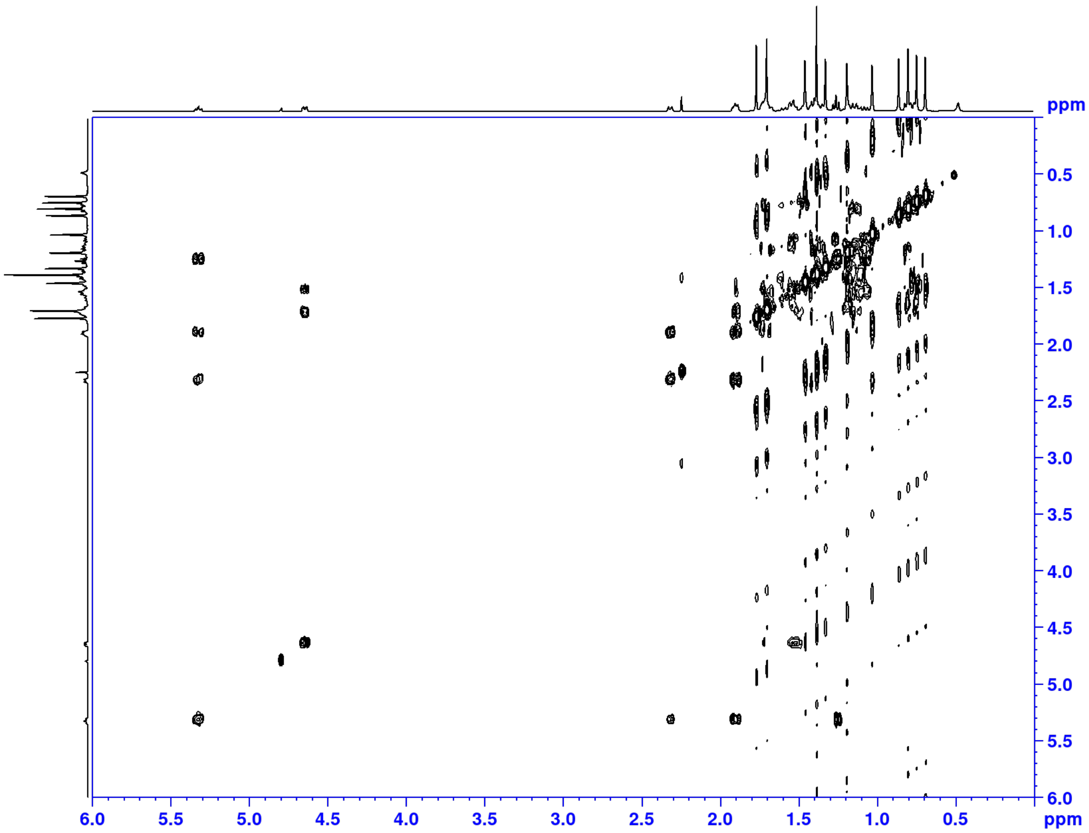

Supplementary Fig. 67. ^{1}H-^{1}H COSY spectrum of **11** in C_6D_6.

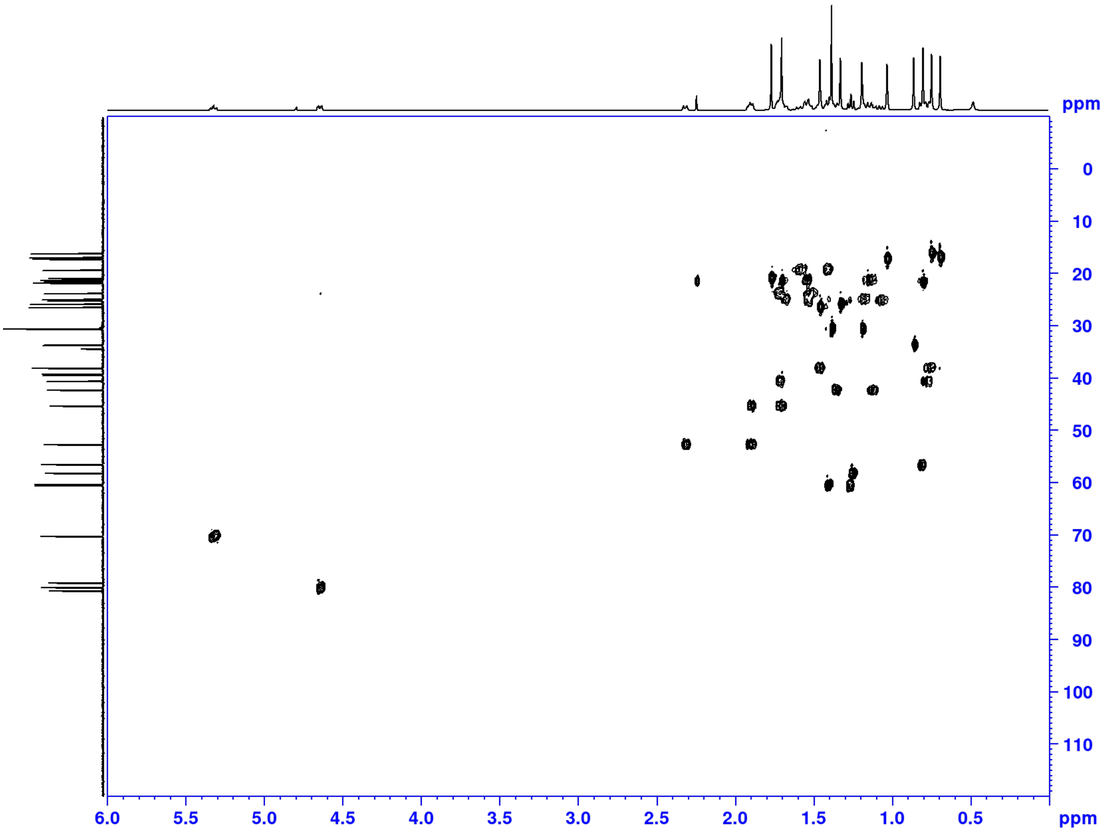

Supplementary Fig. 68. HSQC spectrum of **11** in C_6D_6.

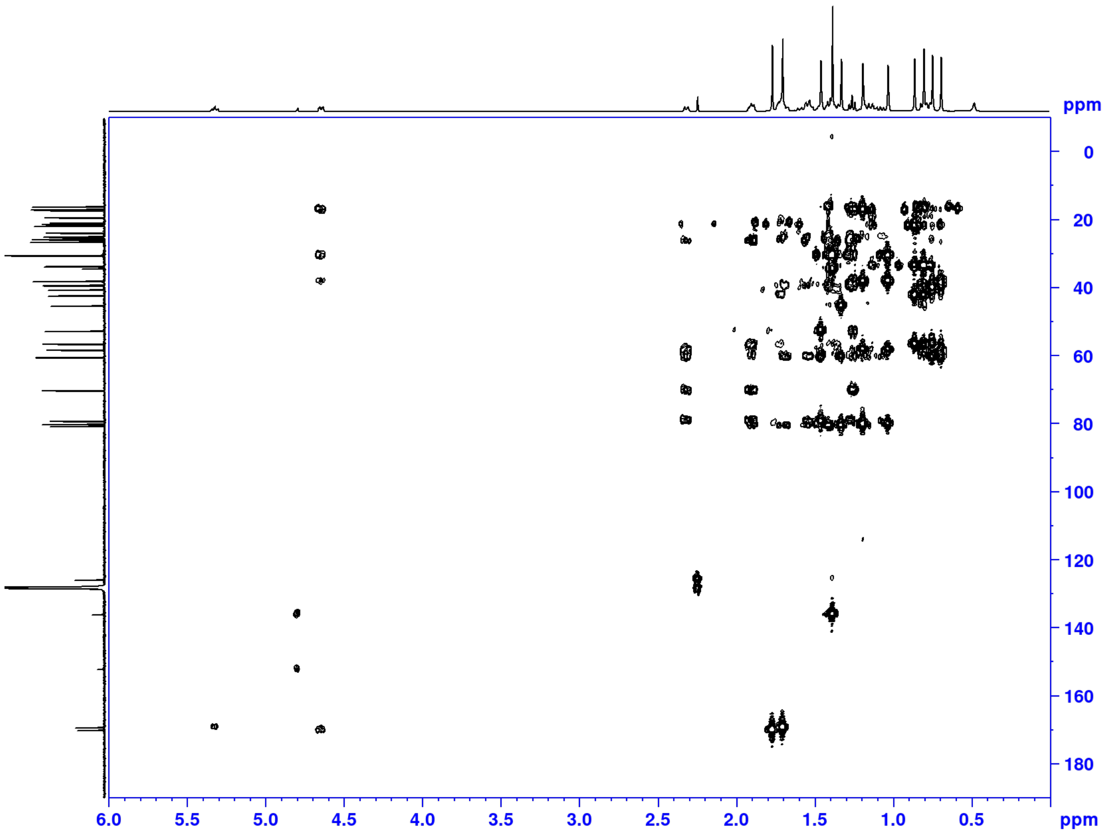

Supplementary Fig. 69. HMBC spectrum of **11** in C_6D_6.

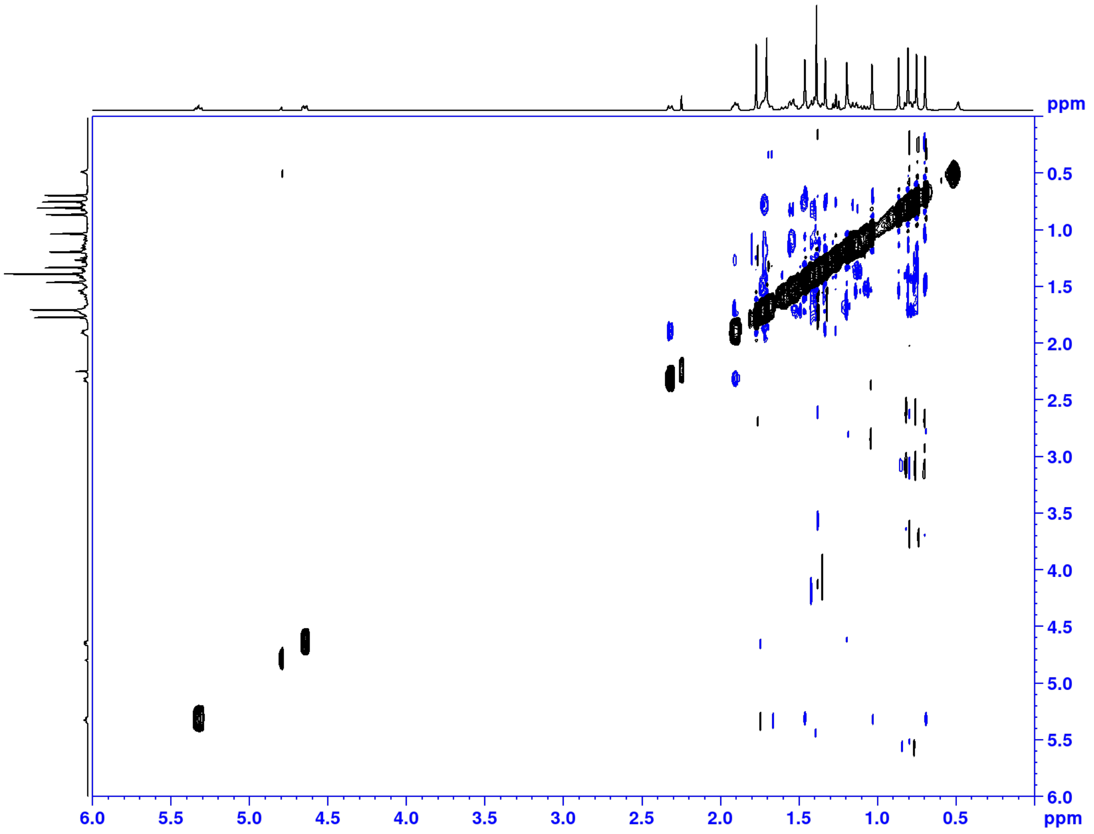

Supplementary Fig. 70. NOESY spectrum of **11** in C_6D_6.

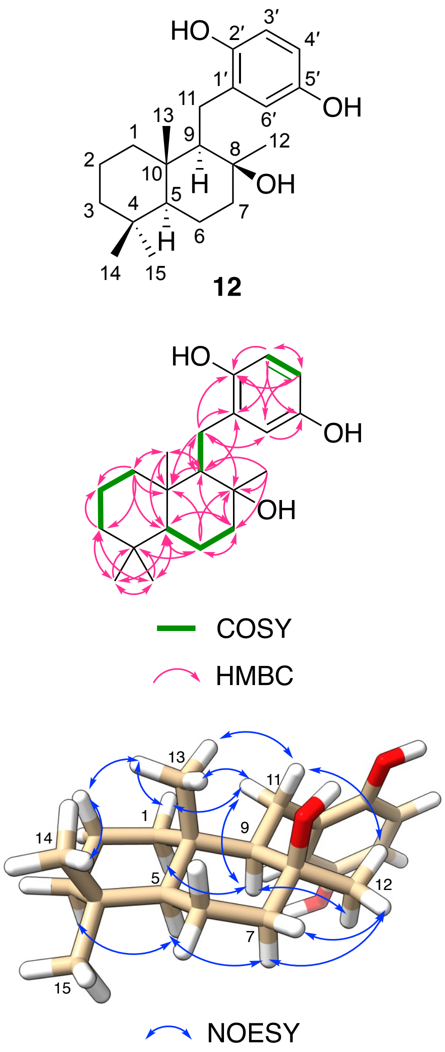

position	δ_C, type	δ_H, mult. (*J* in Hz)
1	40.2, CH_2	1.01 (α), td (13.2, 3.6)
		1.88 (β), brd (12.7)
2	18.5, CH_2	1.44 (α), m
		1.61 (β), qt (13.7, 3.4)
3	41.9, CH_2	1.17 (α), td (13.5, 4.3)
		1.42 (β), m
4	33.4, C	
5	55.8, CH	0.91, overlapped
6	18.3 CH_2	1.52, m
7	43.5, CH_2	1.54 (α), m
		1.75 (β), m
8	73.7, C	
9	58.9, CH	1.45, dd (8.8, 2.6)
10	38.8, C	
11	23.8, CH_2	2.54 (α), dd (15.6, 2.6)
		2.89 (β), dd (15.5, 8.8)
12	31.6, CH_3	0.90, s
13	15.4, CH_3	1.07, s
14	22.0, CH_3	0.87, s
15	33.6, CH_3	0.90, s
1′	131.6, C	
2′	147.5, C	
3′	116.6, CH	6.65, d (8.5)
4′	113.2, CH	6.52, dd (8.5, 3.0)
5′	149.4, C	
6′	116.3, CH	6.68, d (2.9)

^{1}H NMR: 600 MHz, ^{13}C NMR: 150 MHz (in $CDCl_3$)

Supplementary Fig. 71. NMR data of moserinol (**12**).

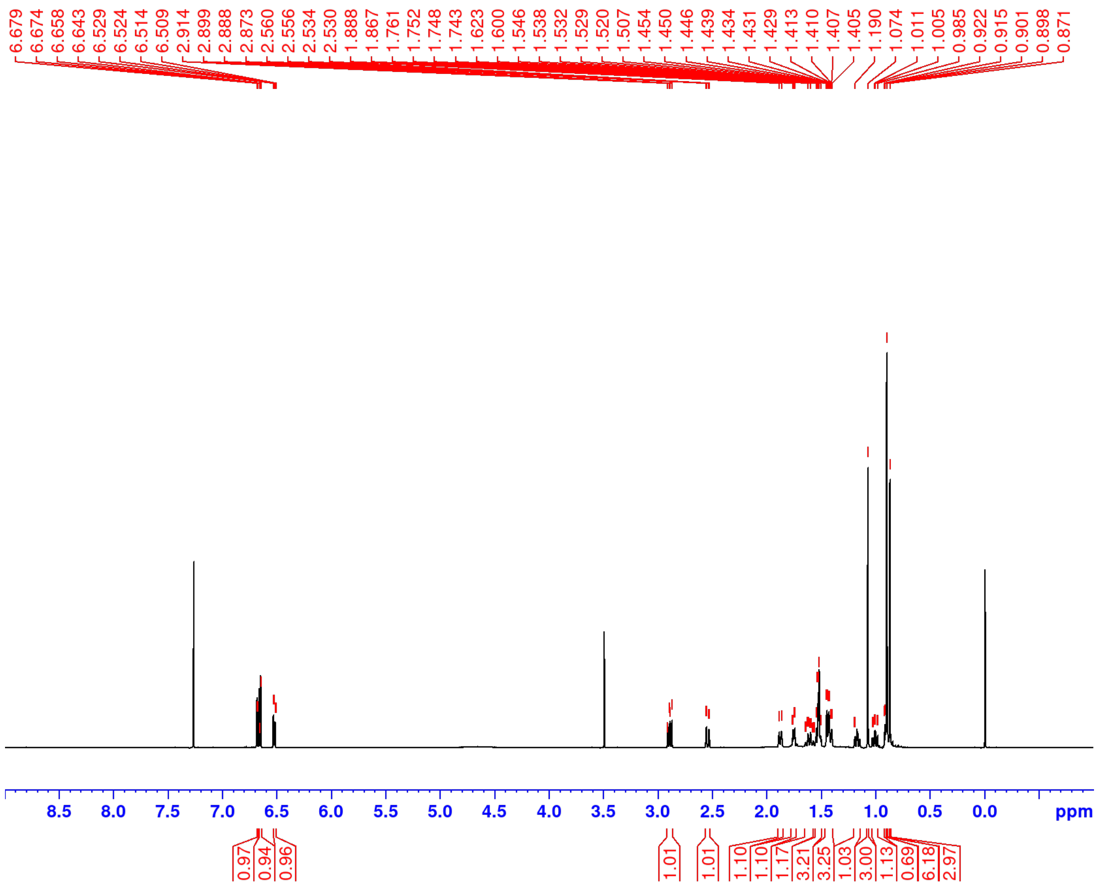

Supplementary Fig. 72. ^{1}H NMR spectrum of **12** in $CDCl_3$ at 600 MHz.

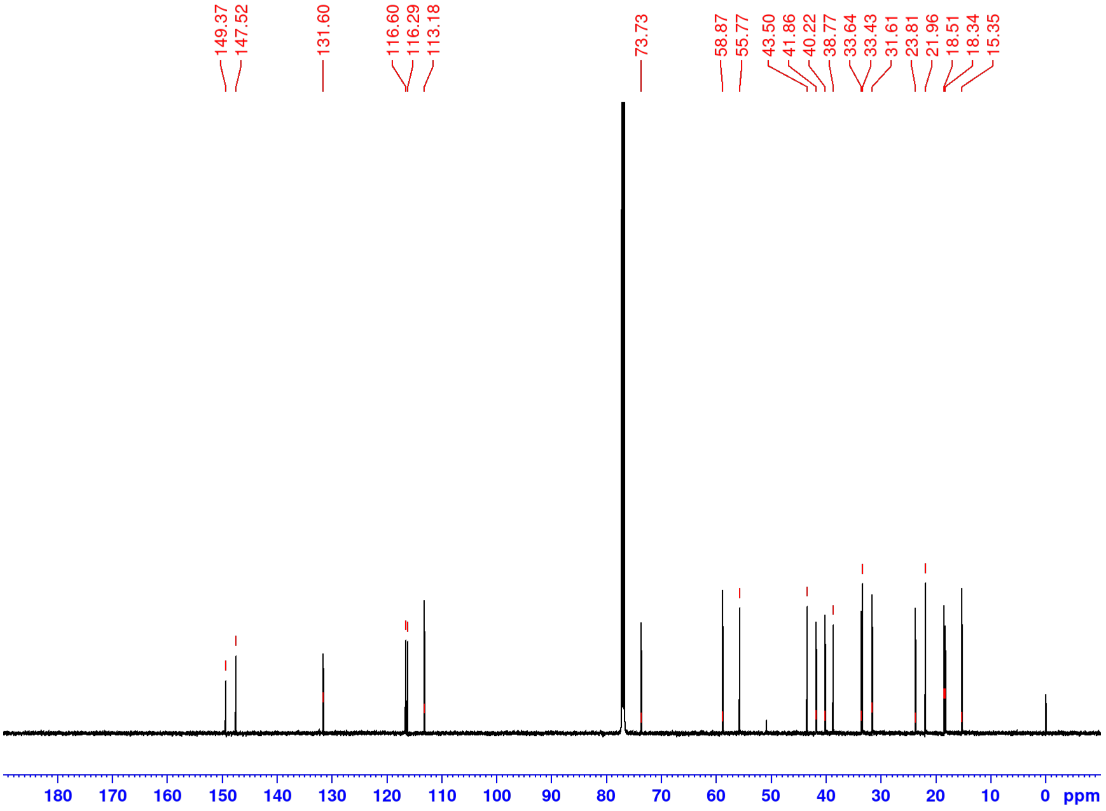

Supplementary Fig. 73. ^{13}C NMR spectrum of **12** in $CDCl_3$ at 150 MHz.

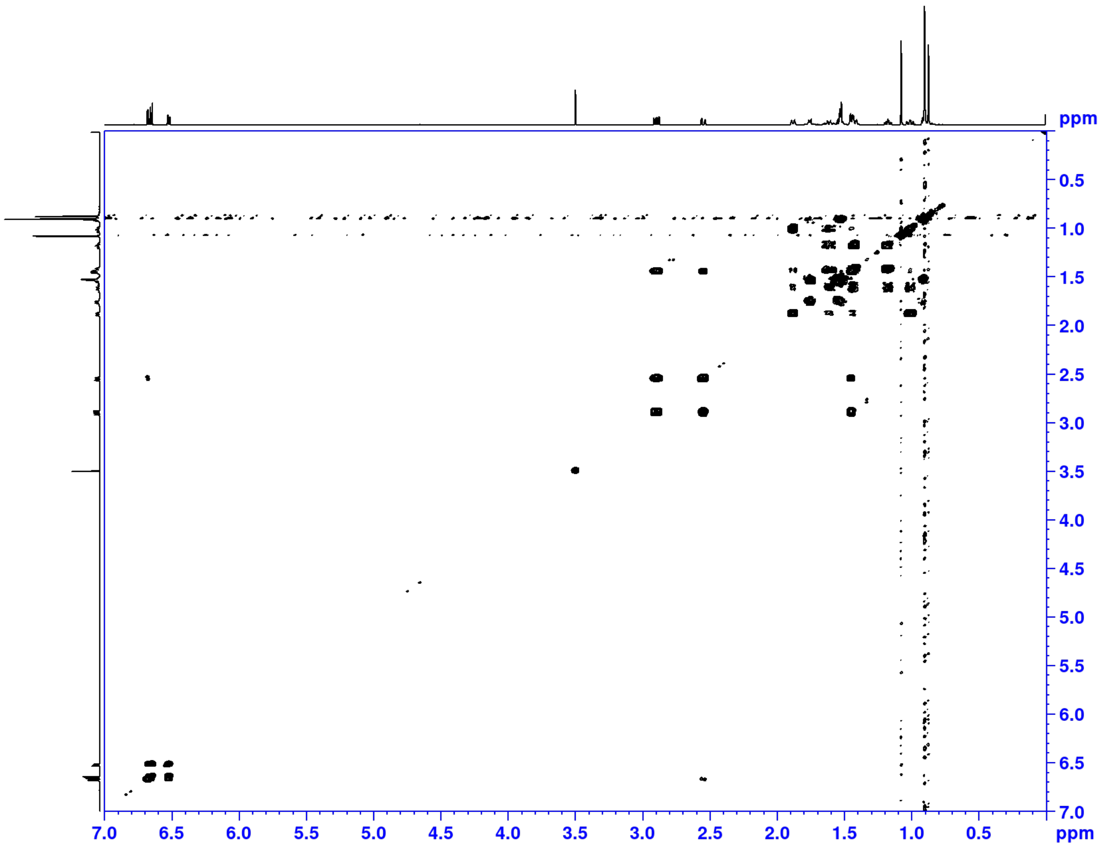

Supplementary Fig. 74. ^{1}H-^{1}H COSY spectrum of **12** in $CDCl_3$.

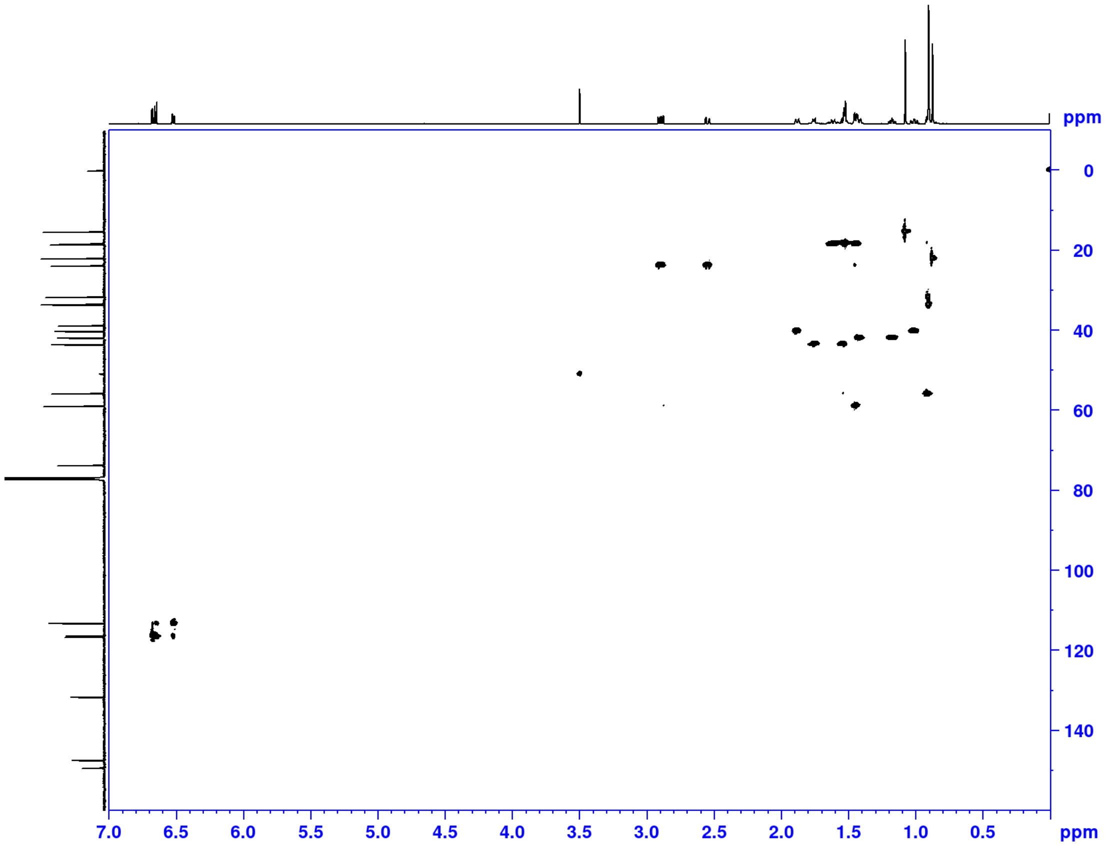

Supplementary Fig. 75. HSQC spectrum of **12** in $CDCl_3$.

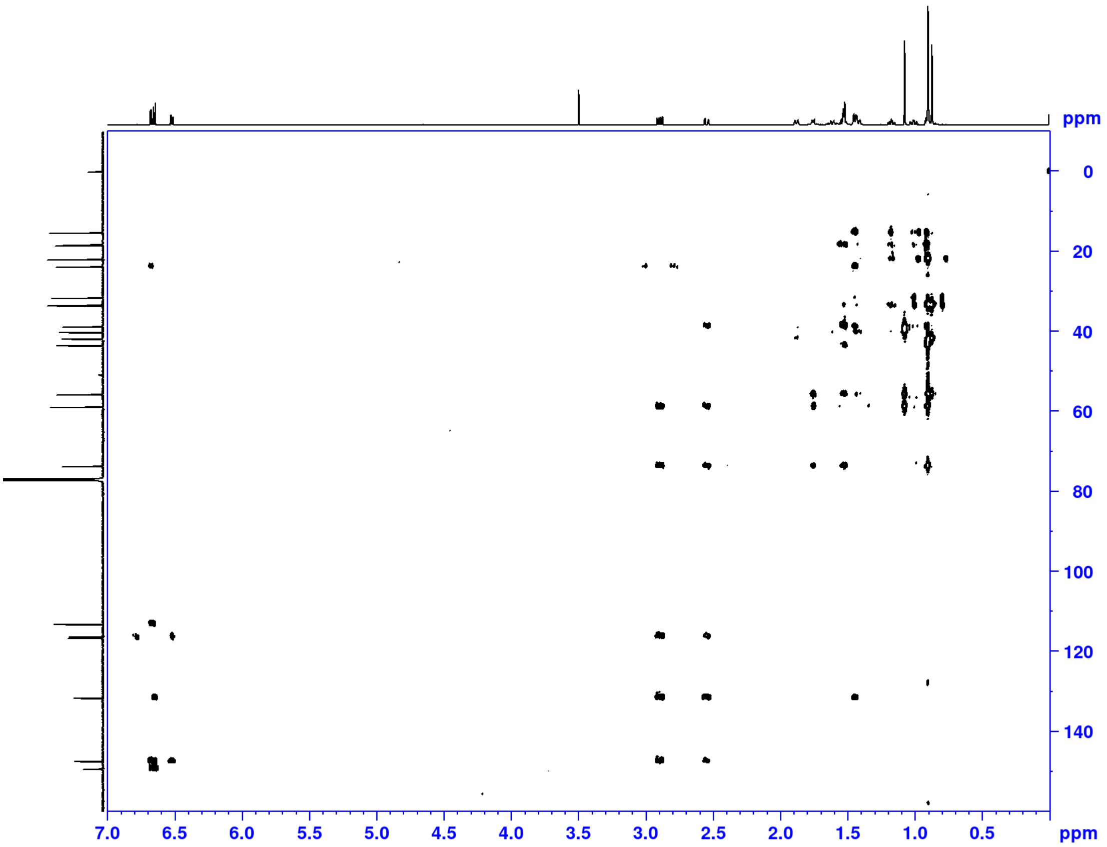

Supplementary Fig. 76. HMBC spectrum of **12** in $CDCl_3$.

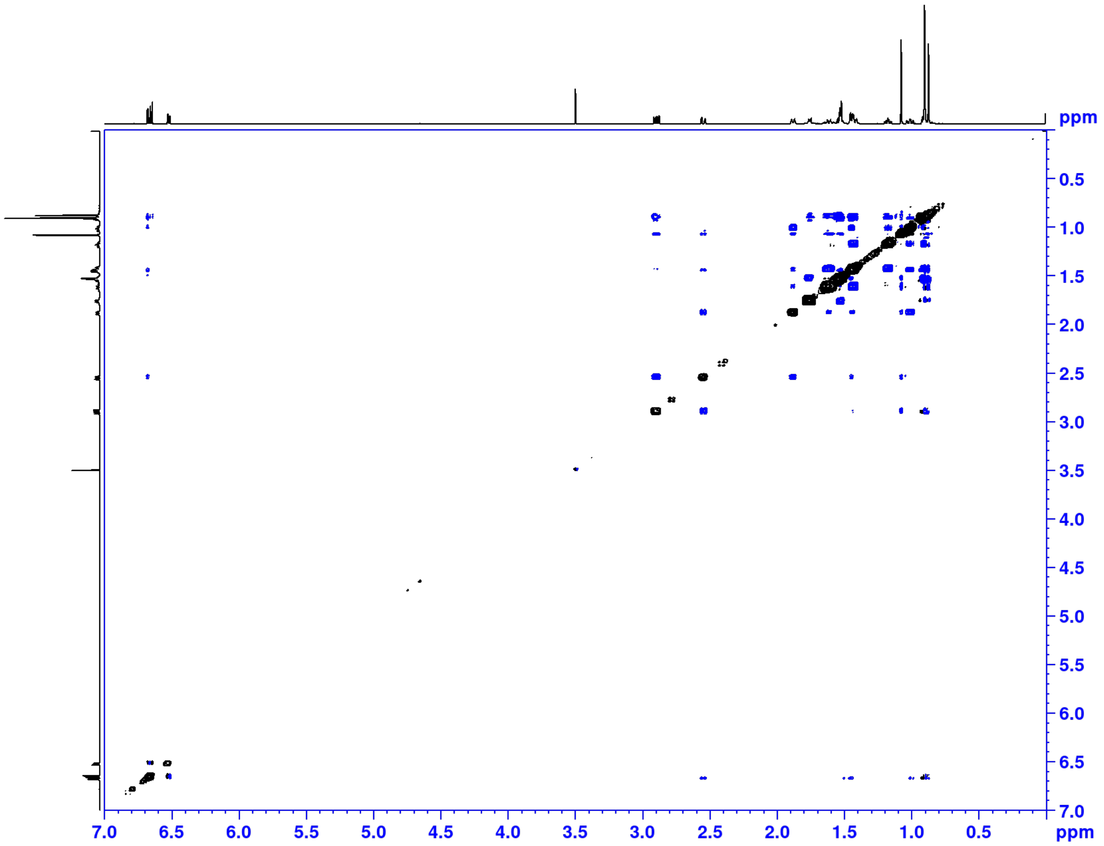

Supplementary Fig. 77. NOESY spectrum of **12** in $CDCl_3$.

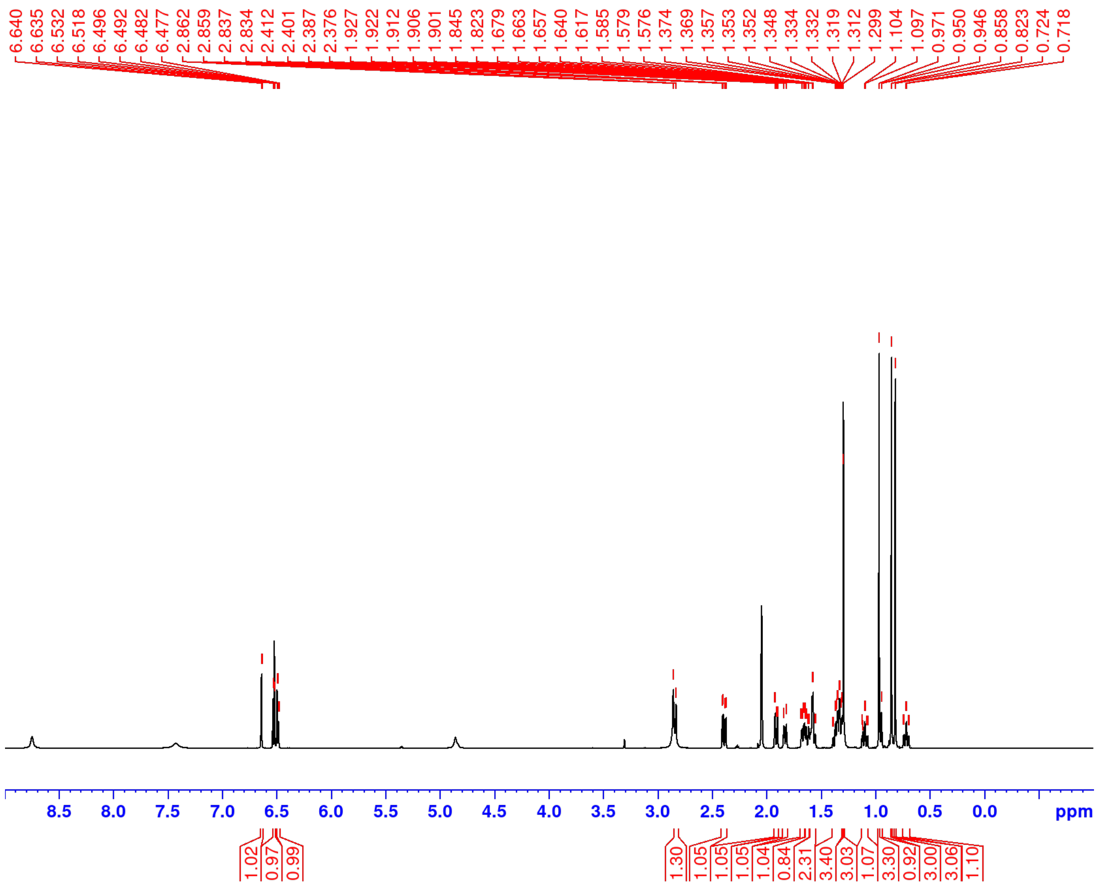

Supplementary Fig. 78. ^{1}H NMR spectrum of **13** in acetone-d_6 at 600 MHz.

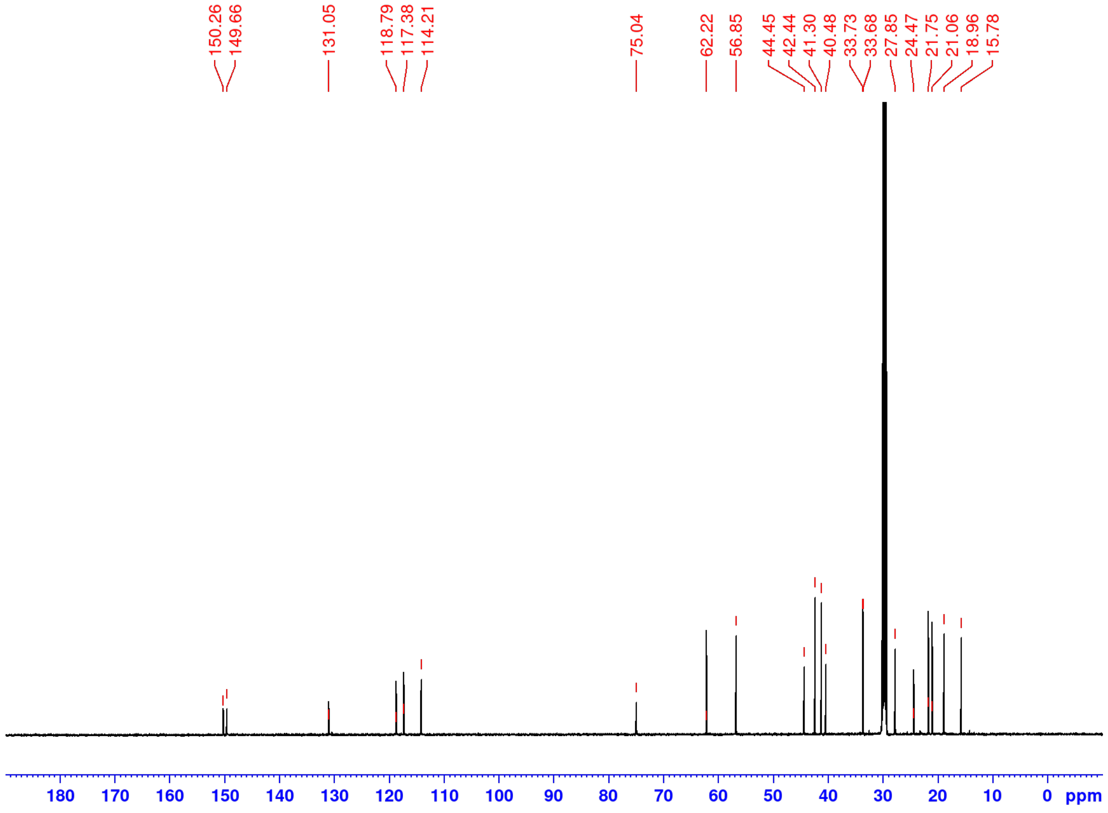

Supplementary Fig. 79. ^{13}C NMR spectrum of **13** in acetone-d_6 at 150 MHz.
