# Supplementary material for: Discovery of fungal onoceroid triterpenoids through domainless enzyme-targeted global genome mining
Source: Nat Commun. 2024 May 21;15:4312. doi: 10.1038/s41467-024-48771-7 (PMC11109268; doi:10.1038/s41467-024-48771-7)
Supplement: Supplementary file 3 — Description of Additional Supplementary Files [file 41467_2024_48771_MOESM3_ESM.pdf]

Description of Additional Supplementary Files

File Name: Supplementary Data 1
Description: Summary of the fungal BGCs manually curated in this study.

File Name: Supplementary Data 2
Description: HMM profiles used to detect fungal biosynthetic proteins.

File Name: Supplementary Data 3
Description: Information on the custom-made HMM profiles.

File Name: Supplementary Data 4
Description: List of fungal genomes used in this study.

File Name: Supplementary Data 5
Description: Summary of the BGCs extracted using FunBGCeX.

File Name: Supplementary Data 6
Description: Summary of the BGCs extracted using antiSMASH.

File Name: Supplementary Data 7
Description: Pyr4 homologues identified in this study.

File Name: Supplementary Data 8
Description: Revised and manually added DNA and protein sequences.

File Name: Supplementary Data 9
Description: Primers used in this study.
