# Supplementary material for: Discovery of fungal onoceroid triterpenoids through domainless enzyme-targeted global genome mining
Source: Nat Commun. 2024 May 21;15:4312. doi: 10.1038/s41467-024-48771-7 (PMC11109268; doi:10.1038/s41467-024-48771-7)
Supplement: Supplementary file 13 — Reporting Summary [file 41467_2024_48771_MOESM13_ESM.pdf]

nature portfolio

Corresponding author(s): Yudai Matsuda

Last updated by author(s): Apr 27, 2024

Reporting Summary

Nature Portfolio wishes to improve the reproducibility of the work that we publish. This form provides structure for consistency and transparency in reporting. For further information on Nature Portfolio policies, see our Editorial Policies and the Editorial Policy Checklist.

Statistics

For all statistical analyses, confirm that the following items are present in the figure legend, table legend, main text, or Methods section.

n/a	Confirmed	
☐	☒	The exact sample size (*n*) for each experimental group/condition, given as a discrete number and unit of measurement
☒	☐	A statement on whether measurements were taken from distinct samples or whether the same sample was measured repeatedly
☒	☐	The statistical test(s) used AND whether they are one- or two-sided<br>*Only common tests should be described solely by name; describe more complex techniques in the Methods section.*
☒	☐	A description of all covariates tested
☒	☐	A description of any assumptions or corrections, such as tests of normality and adjustment for multiple comparisons
☐	☒	A full description of the statistical parameters including central tendency (e.g. means) or other basic estimates (e.g. regression coefficient) AND variation (e.g. standard deviation) or associated estimates of uncertainty (e.g. confidence intervals)
☒	☐	For null hypothesis testing, the test statistic (e.g. *F*, *t*, *r*) with confidence intervals, effect sizes, degrees of freedom and *P* value noted<br>*Give P values as exact values whenever suitable.*
☒	☐	For Bayesian analysis, information on the choice of priors and Markov chain Monte Carlo settings
☒	☐	For hierarchical and complex designs, identification of the appropriate level for tests and full reporting of outcomes
☒	☐	Estimates of effect sizes (e.g. Cohen's *d*, Pearson's *r*), indicating how they were calculated

Our web collection on statistics for biologists contains articles on many of the points above.

Software and code

Policy information about availability of computer code

Data collection	NCBI Datasets (version 15.30.0) was used to download the fungal genome sequence data.
Data analysis	FunBGCex (version 0.0.0) and antiSMASH (version 6.1.1/7.0.0) were used to extract biosynthetic gene clusters from fungal genomes. MUSCLE (version 5.1), Gblocks (version 0.91b), FastTree (version 2.1.11), RaxML (version 8.0.0), ProtTest (version 3.4.2) were used to construct phylogenetic trees. Geneious Prime 2023.2.1 was used to visualize phylogenetic trees.<br>All original codes for FunBGCeX are deposited at Zenodo under the DOI 10.5281/zenodo.8126797 and are also available at https://github.com/ydmatsd/funbgcex.

For manuscripts utilizing custom algorithms or software that are central to the research but not yet described in published literature, software must be made available to editors and reviewers. We strongly encourage code deposition in a community repository (e.g. GitHub). See the Nature Portfolio guidelines for submitting code & software for further information.

Data

Policy information about availability of data

All manuscripts must include a data availability statement. This statement should provide the following information, where applicable:

- Accession codes, unique identifiers, or web links for publicly available datasets
- A description of any restrictions on data availability
- For clinical datasets or third party data, please ensure that the statement adheres to our policy

The manually curated fungal BGCs and custom-made HMM profiles are deposited at Zenodo under the DOI 10.5281/zenodo.8126803. The online version of the FunBGCs database can currently be accessed at http://staffweb1.cityu.edu.hk/ymatsuda/funbgcs/funbgcs.html. The corresponding genomic regions for the biosynthetic gene clusters reported in this study can be accessed at the National Center for Biotechnology Information (NCBI) database with the accession numbers NW_020291903 (the homo cluster; region: 95,123...101,061), DS499601 (the fumi cluster; region: 75,155...91,115), NW_022474693 (the alli cluster; region: 128,036...138,909), and NW_026055164 (the mos cluster; region: 259,950...270,410). The crystallographic data of compounds 5, 7, 9, 10, and 11 are deposited at the Cambridge Crystallographic Data Centre (CCDC) under deposition numbers 2279439, 2279440, 2279441, 2279442, and 2279443, respectively. The raw NMR data of compounds 1–13 are deposited at the Natural Product Magnetic Resonance Database (NP-MRD) under deposition numbers NP0332710–NP0332722.

Research involving human participants, their data, or biological material

Policy information about studies with human participants or human data. See also policy information about sex, gender (identity/presentation), and sexual orientation and race, ethnicity and racism.

Reporting on sex and gender	N/A
Reporting on race, ethnicity, or other socially relevant groupings	N/A
Population characteristics	N/A
Recruitment	N/A
Ethics oversight	N/A

Note that full information on the approval of the study protocol must also be provided in the manuscript.

Field-specific reporting

Please select the one below that is the best fit for your research. If you are not sure, read the appropriate sections before making your selection.

☒ Life sciences ☐ Behavioural & social sciences ☐ Ecological, evolutionary & environmental sciences

For a reference copy of the document with all sections, see nature.com/documents/nr-reporting-summary-flat.pdf

Life sciences study design

All studies must disclose on these points even when the disclosure is negative.

Sample size	The number of fungal genomes used in this study was determined based on those available as of April, 2023. At least three individual transformants were analyzed upon construction of Aspergillus oryzae transformants to confirm consistency among transformants.
Data exclusions	No data exclusions have been applied.
Replication	Analyses of fungal metabolites and gene expressions were performed at least twice to confirm consistency of the results. Evaluation of the biological activity was performed in triplicate. All attempts at replication were successful.
Randomization	Randomization is not relevant to this study, as no samples were allocated to experimental groups.
Blinding	Blinding is not relevant to this study, as it does not involve human subjects.

Reporting for specific materials, systems and methods

We require information from authors about some types of materials, experimental systems and methods used in many studies. Here, indicate whether each material, system or method listed is relevant to your study. If you are not sure if a list item applies to your research, read the appropriate section before selecting a response.

Materials & experimental systems

n/a	Involved in the study
☒	☐ Antibodies
☒	☐ Eukaryotic cell lines
☒	☐ Palaeontology and archaeology
☒	☐ Animals and other organisms
☒	☐ Clinical data
☒	☐ Dual use research of concern
☒	☐ Plants

Methods

n/a	Involved in the study
☒	☐ ChIP-seq
☒	☐ Flow cytometry
☒	☐ MRI-based neuroimaging

Plants

Seed stocks	N/A
Novel plant genotypes	N/A
Authentication	N/A
